# Botryane terpenoids produced by *Nemania bipapillata*, an endophytic fungus isolated from red alga *Asparagopsis taxiformis* - *Falkenbergia* stage

**DOI:** 10.1038/s41598-019-48655-7

**Published:** 2019-08-23

**Authors:** Rebeca P. Medina, Angela R. Araujo, João M. Batista, Carmen L. Cardoso, Cláudia Seidl, Adriana F. L. Vilela, Helori V. Domingos, Leticia V. Costa-Lotufo, Raymond J. Andersen, Dulce H. S. Silva

**Affiliations:** 1 Núcleo de Bioensaios, Biossíntese e Ecofisiologia de Produtos Naturais (NuBBE), Departamento de Química Orgânica, Instituto de Química, UNESP - Universidade Estadual Paulista, 14801-970 Araraquara-SP, Brazil; 2 Departamento de Química, Centro de Ciências Exatas e de Tecnologia, Universidade Federal de São Carlos - UFSCar, 13565-905 São Carlos-SP, Brazil; 3 Departamento de Ciência e Tecnologia, Universidade Federal de São Paulo –UNIFESP, 12231-280 São José dos Campos-SP, Brazil; 4 Grupo de Cromatografia de Bioafinidade e Produtos Naturais, Departamento de Química, Faculdade de Filosofia, Ciências e Letras de Ribeirão Preto, Universidade de São Paulo, 14040-901 Ribeirão Preto-SP, Brazil; 5 Instituto de Ciências Biomédicas, Universidade de São Paulo, 05508-900 São Paulo-SP, Brazil; 6 Departments of Chemistry and Earth, Ocean & Atmospheric Sciences, University of British Columbia, V6T 1Z1 Vancouver, BC Canada

**Keywords:** Natural products, Structure elucidation

## Abstract

A chemical study of the EtOAc extract of *Nemania bipapillata* (AT-05), an endophytic fungus isolated from the marine red alga *Asparagopsis taxiformis* - *Falkenbergia* stage, led to the isolation of five new botryane sesquiterpenes, including the diastereomeric pair (+)-(2*R*,4*S*,5*R*,8*S*)-(**1**) and (+)-(2*R*,4*R*,5*R*,8*S*)-4-deacetyl-5-hydroxy-botryenalol (**2**), (+)-(2*R*,4*S*,5*R*,8*R*)-4-deacetyl-botryenalol (**3**), one pair of diastereomeric botryane *nor*sesquiterpenes bearing an unprecedented degraded carbon skeleton, (+)-(2*R*,4*R*,8*R*)-(**4**) and (+)-(2*R*,4*S*,8*S*)-(**5**), which were named nemenonediol A and nemenonediol B, respectively, in addition to the known 4β-acetoxy-9β,10β,15α-trihydroxyprobotrydial (**6**). Their structures were elucidated using 1D and 2D NMR, HRESIMS and comparison with literature data of similar known compounds. The absolute configurations of **2**, **3** and **4** were deduced by comparison of experimental and calculated electronic circular dichroism (ECD) spectra, while those of **1** and **5** were assigned from vibrational circular dichroism (VCD) data. Compound **4** weakly inhibited acetylcholinesterase, whereas compound **1** inhibited both acetylcholinesterase and butyrylcholinesterase. Compounds **1**, **3**, **5** and **6** were tested against two carcinoma cell lines (MCF-7 and HCT-116), but showed no significant citotoxicity at tested concentrations (IC_50_ > 50 µM).

## Introduction

Recent studies on marine-derived endophytic microorganisms have shown their potential as a source of new bioactive natural products^1^. As part of our ongoing efforts to identify new secondary metabolites produced by endophytic fungi isolated from the red alga *Asparagopsis taxiformis* (*Falkenbergia* stage)^2^, we have investigated extracts of laboratory cultures of the fungus *Nemania bipapillata*. This chemical study led to the isolation of one known and five new botryane terpenoids, including two pairs of diastereomeric compounds.

The genus *Nemania* belongs to the family Xylariaceae, one of the largest of Ascomycota phylum^3^, and has shown interesting applications associated with its bioactive small molecules as well as in biocatalysis. *Nemania serpens* was isolated as a fungal endophyte from *Anemopsis californica*, a plant used as a traditional medicine to treat infections due to its antibacterial activity against *Staphylococcus aureus* and *Pseudomonas aeruginosa*^4^. *Nemania aenea* SF10099-1, isolated from a forest soil sample was used as a bioacatalyst in the regio- and stereoselective synthesis of (−)-β-caryophyllene oxide using β-caryophyllene as substrate in a liquid–liquid interface bioreactor^5^.

Acetylcholinesterase (EC 3.1.1.7) (AChE) and butyrylcholinesterase (EC 3.1.1.8) (BChE) have gained attention due to their roles in Alzheimer disease (AD) and other Central Nervous System (CNS) conditions, such as delirium and traumatic brain injuries, associated with deficient levels of acetylcholine (ACh)^6^. Galanthamine, a natural alkaloid first isolated from the plant *Galanthus* spp. (Amaryllidaceae), is one of the few drugs currently available for the treatment of AD, which inhibits the AChE activity, increasing the levels of ACh in the brain and prolonging cholinergic functions^7^. Diterpenoids have been shown to be cholinesterase inhibitors^8–11^, which stimulated our interest in investigating the potential anticholinesterase activity of the new terpenoids isolated from *Nemania bipapillata*.

**Figure 1:**
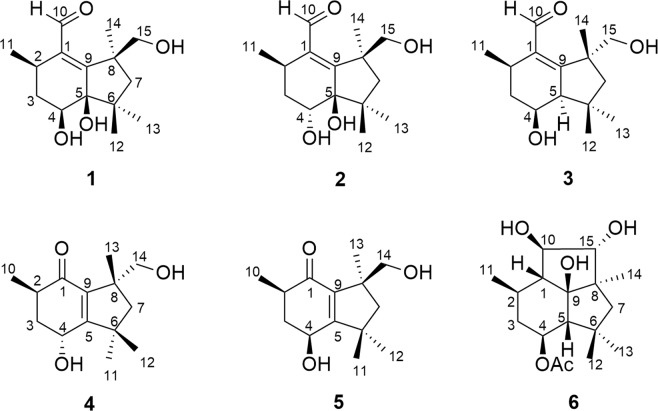
Botryane terpenoids isolated from EtOAc extract of *Nemania bipapillata* cultures.

The search for new cancer chemotherapeutic agents has increased in the last years due to the drug resistance in cancer treatment. Natural sesquiterpenes have demonstrated cytotoxic activity^12^, including botrydial, a botryane sesquiterpene, and its derivatives^13^. In this paper, we describe the isolation and structure elucidation of three new botryane sesquiterpenes (**1**–**3**) and two *nor*sesquiterpenes with a new degraded carbon skeleton (**4** and **5**) (Fig. 1), the determination of their absolute configurations, and their activities as cholinesterase inhibitors and cytotoxic agents.

For the determination of the absolute configuration of compounds **1**–**5**, electronic and vibrational circular dichroism spectroscopies (ECD and VCD, respectively) were used with the aid of quantum chemical calculations. ECD and VCD methods are based on the differential absorption of left- and right-circularly polarized UV or IR radiation, respectively, by a chiral molecule during electronic or vibrational transitions. Both chiroptical methods have been successfully used over the years for absolute configuration assignments of a large array of chiral molecules including natural products^14,15^.

## Results and Discussion

The EtOAc extract of *Nemania bipapillata* (AT-05) cultures was fractionated using a reversed-phase flash chromatography column to give 4 fractions (AT-05-F1 - AT-05-F4). Further purification of fraction AT-05-F2 using reversed-phase HPLC yielded five new compounds **1–5**, in addition to the known compound **6** (Fig. 1). Their structures were elucidated via detailed analysis of NMR and mass spectrometry data, in addition to ECD and VCD analyses to determine their absolute configurations.

**Figure 2:**
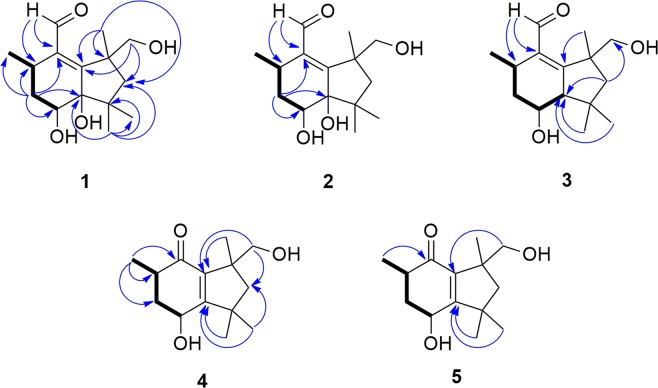
COSY (bold bonds) and key HMBC (blue arrows) correlations of compounds **1**–**5**.

**Figure 3:**
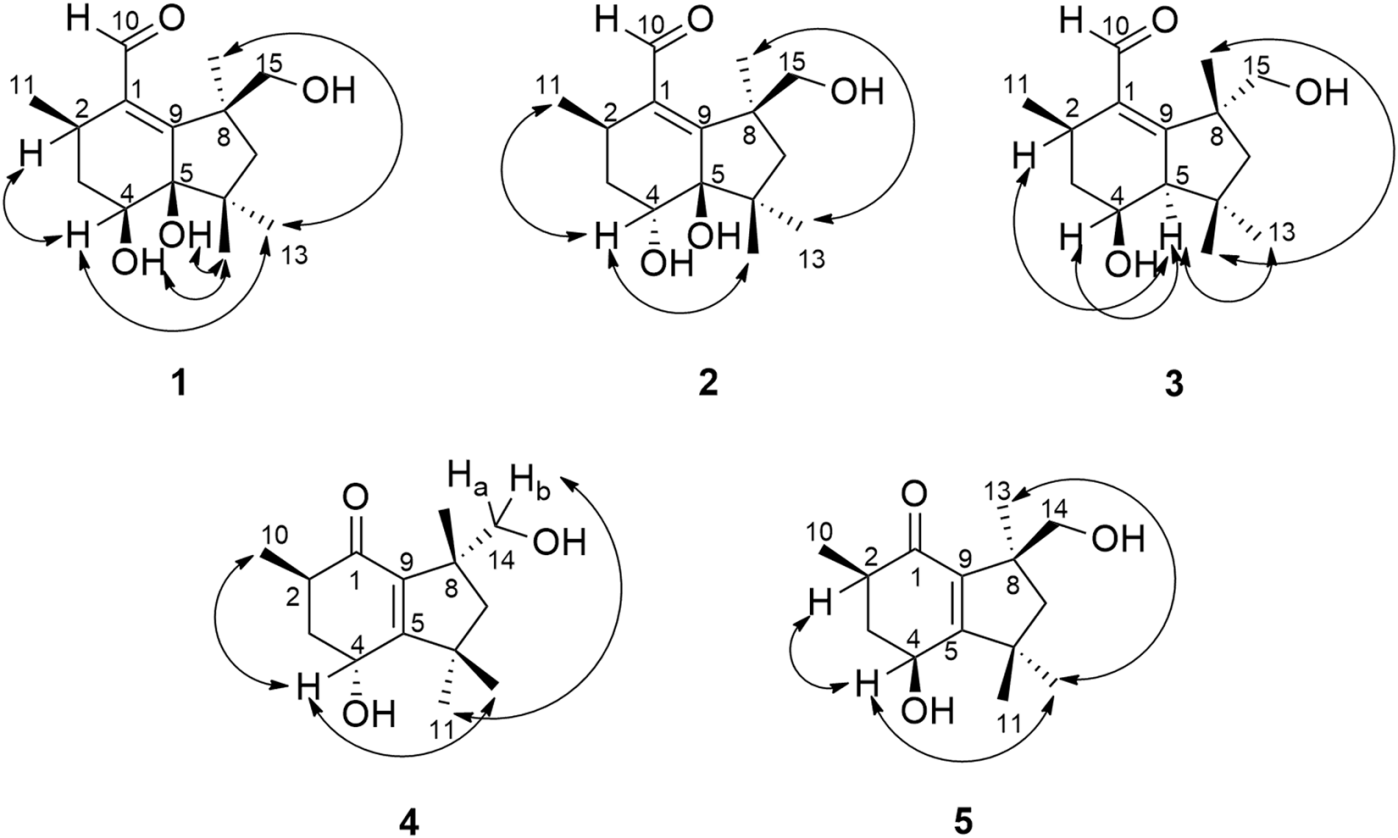
ROESY correlations for compound **1** and NOESY correlations for compounds **2**–**5**.

Compound **1** gave a [M + Na]^+^ ion at *m/z* 291.1576 in the ESITOFHRMS appropriate for a molecular formula of C_15_H_24_O_4_ (calcd for C_15_H_24_O_4_Na 291.1572) requiring 4 sites of unsaturation. The ^1^H NMR (Table 1) and HSQC spectra revealed the presence of four methyl groups, associated with three singlets (δ 1.20/H_3_-12, 1.00/H_3_-13 and 1.40/H_3_-14) and one doublet (δ 1.03, *J*_11-2_ 6.6 Hz, H_3_-11), one hydroxymethine proton at δ 3.73 (dd, *J*_4-3a_ 4.2, *J*_4-3b_ 12.0 Hz, H-4) and a set of nonequivalent oxymethylene protons at δ 3.71 (d, *J*_15a-15b_ 10.2 Hz, H_a_-15)/3.57 (d, *J*_15b-15a_ 10.2 Hz, H_b_-15), in addition to one aldehyde proton (δ 10.28, s, H-10). The ^1^H‒^1^H COSY spectrum (Supplementary Fig. S4) showed correlations of a methine proton at δ 2.73 (m, H-2) to methylene hydrogens at δ 1.81 (m, H_a_-3, 1H) and δ 1.67 (m, H_b_-3, 1H) and to H_3_-11 (Fig. 2). The ^13^C NMR data (Table 2) exhibited one carbonyl carbon at δ 197.1 (C-10), which correlated with the proton at δ 10.28, as shown in the HSQC spectrum (Supplementary Fig. S5), and two quaternary sp^2^ carbons (δ 141.8/C-1, 164.7/C-9), suggesting the presence of an α,β-unsaturated carbonyl group, in addition to three quaternary sp^3^ (δ 44.6/C-6, 47.9/C-8, 83.1/C-5) hybridized carbons. The HMBC spectrum (Supplementary Fig. S6) evidenced correlations of H-10 to C-1 and C-2 (δ 31.5), of H_a_-3 to C-1, C-4 (δ 69.9) and C-5, of H_b_-3 to C-2, C-4 (δ 69.9) and C-11 (δ 20.9), of H_3_-12 to C-5, C-6, C-7 (δ 53.4) and C-13 (δ 26.6), of H_3_-14 to C-7, C-8 and C-9, as well as correlations of H_a_-15 and H_b_-15 to C-7 and C-9, respectively (Fig. 2). The ^1^H NMR spectrum of compound **1** obtained in DMSO-*d*_6_ displayed signals for the hydroxyl protons at δ 5.40 (t, *J*_15-0H-15_ 4.8 Hz, 15-OH, 1H), 4.33 (s, 5-OH, 1H), and 4.23 (d, *J*_4-OH-4_ 7.2 Hz, 4-OH, 1H) (Supplementary Table S1 and Fig. S7). ROESY (DMSO-*d*_6_) correlations revealed the relative configuration of **1** (Supplementary Fig. S9). The 4-OH and 5-OH signals showed ROESY correlations to H_3_-12 (δ 1.09 s, 3H), whereas the H-4 signal (δ 3.53, ddd, *J*_4-3a_ 4.5, *J*_4-4-OH_ 7.5, *J*_4-3b_ 12.3 Hz, 1H) showed ROESY correlations to H-2 (δ 2.60, m, 1H) and H_3_-13 (δ 0.90, s, 3H). Finally, a ROESY correlation observed between H_3_-13 and H_3_-14 (δ 1.33, s, 3H) (Fig. 3) established the relative configuration at C-8, completing the assignment of the constitution and relative configuration of **1**.

**Table 1:** ^1^H NMR data (δ in ppm, mult., *J* in Hz) of **1–5** (600 MHz; CD_3_OD).

Position	1	2	3	4	5
2	2.73 (m)	2.91 (m)	2.79 (m)	2.78 (m)	2.43 (m)
3	1.81 (m)	1.80 (m)	2.02 (ddd, 3.9, 6.5, 12.8)	2.09 (m)	2.27 (m)
	1.67 (m)		1.29 (m)	1.95 (m)	1.75 (m)
4	3.73 (dd, 4.2, 12.0)	4.10 (dd, 3.6, 6.9)	3.62 (ddd, 3.9, 8.7, 11.1)	4.43 (t, 3.0)	4.69 (dd, 4.8, 10.2)
5			2.33 (dd, 3.3, 8.7)		
7	2.23 (d, 13.0)	2.26 (d, 12.6)	1.88 (d, 13.2)	1.96 (d, 13.2)	1.93 (d, 13.8)
	1.24 (d, 13.0)	1.19 (d, 12.6)	1.40 (d, 13.2)	1.53 (d, 13.2)	1.53 (d, 13.8)
10	10.28 (s)	10.26 (s)	10.21 (s)	1.10 (d, 7,2)	1.10 (d, 6.6)
11	1.03 (d, 6.6)	1.05 (d, 7.2)	1.06 (d; 7.2)	1.32 (s)	1.38 (s)
12	1.20 (s)	1.15 (s)	0.98 (s)	1.20 (s)	1.31 (s)
13	1.00 (s)	1.16 (s)	1.25 (s)	1.19 (s)	1.13 (s)
14	1.40 (s)	1.48 (s)	1.44 (s)	3.63 (d, 10.8)	3.57 (d, 10.8)
				3.42 (d, 10.8)	3.54 (d, 10.8)
15	3.71 (d, 10.2)	3.71 (d, 10.2)	3.52 (d, 10.8)		
	3.57 (d, 10.2)	3.57 (d, 10.2)	3.58 (d, 10.8)

**Table 2:** ^13^C NMR data (δ in ppm) of **1–5** (150 MHz; CD_3_OD).

Position	1	2	3	4	5
1	141.8	141.9	139.6	203.9	203.4
2	31.5	27.4	31.4	38.3	43.5
3	36.5	37.9	42.5	42.1	43.6
4	69.9	71.9	69.0	62.4	68.1
5	83.1	83.9	62.7	170.3	174.6
6	44.6	45.1	40.2	46.0	47.3
7	53.4	54.2	55.9	51.8	52.8
8	47.9	47.6	49.1	50.2	50.0
9	164.7	166.1	169.4	140.5	140.2
10	197.1	195.1	195.7	15.1	15.3
11	20.9	20.0	21.4	30.1	30.7
12	23.6	22.7	24.1	28.5	29.1
13	26.6	27.5	30.4	23.7	23.7
14	30.5	30.5	30.3	69.7	70.0
15	71.7	71.8	72.0

Compound **2** was assigned the same molecular formula as **1** (C_15_H_24_O_4_) on the basis of HRMS (ESI-Q-TOF, *m/z*: [M + Na]^+^ calcd for C_15_H_24_O_4_Na 291.1572; found 291.1563) data. Its ^1^H and ^13^C NMR data (Tables 1 and 2) were similar to those of **1**, except for the chemical shifts of C-2 (δ_H/C_ 2.91/27.4) and C-4 (δ_H/C_ 4.10/71.9). Furthermore, the H-4 signal exhibited different coupling constants (*J*_4-3a_ 3.6, *J*_4-3b_ 6.9 Hz, 1H) from those observed for compound **1**, suggesting that **2** was a stereoisomer of **1** with a configurational difference at C-4, which was confirmed from 2D NMR data analyses. The HMBC spectrum of **2** (Supplementary Fig. S16) showed correlations of H-10 (δ 10.26, s, 1H) to C-1 (141.9) and C-2, of H-3 (δ 1.80, m, 2 H) to C-1, C-4 and C-5 (δ 83.9) (Fig. 2), as observed in compound **1**. The relative configuration of **2** was established via 1D NOE experiments (Supplementary Fig. S17). Irradiation of H-4 gave NOEs in H_3_-11 (δ 1.05, *J*_11-2_ 7.2 Hz, 3H) and H_3_-12 (δ 1.15, s, 3H) consistent with a *cis* configuration between H-4 and H_3_-11, which was different from the *cis* configuration between H-4 and H-2 in **1**. Irradiation of H_3_-14 gave an NOE in H_3_-13 confirming that C-8 had the same relative configuration in both **1** and **2** (Fig. 3).

The molecular formula of compound **3** was deduced as C_15_H_24_O_3_ from HRMS data (ESI-Q-TOF, *m/z*: [M + Na]^+^ calcd for C_15_H_24_O_3_Na 275.1623; found 275.1613). Comparison of the NMR data obtained for **3** (Tables 1 and 2) with those of **1** and **2** revealed that compound **3** bears a similar structure. However, its ^1^H NMR spectrum had a double doublet at δ 2.33 (*J*_5-2_ 3.3, *J*_5-4_ 8.7 Hz, 1H), which was assigned to H-5 after observation of a ^1^H‒^1^H COSY correlation to H-4 (δ 3.62, ddd, *J*_4-3a_ 3.9, *J*_4-5_ 8.7, *J*_4-3b_ 11.1 Hz, 1H) (Fig. 2) and a HSQC correlation to the signal at δ 62.7 (C-5) (Supplementary Fig. S22). HOMODEC spectrum evidenced its additional coupling to H-2 (δ 2.79, m, 1H) and confirmed the coupling constant (*J*_5-2_ 3.3 Hz), upon irradiation of H-2 (Supplementary Fig. S23). The HMBC spectrum (Supplementary Figs S24 and S25) exhibited correlations of H_b_-7 (δ 1.40, d, *J*_7b-7a_ 13.2 Hz, 1H), H_3_-12 (δ 0.98, s, 3H) and H_3_-13 (δ 1.25, s, 3H) to C-5 (Fig. 2), coherent with the structure proposed for compound **3**. NOE experiments disclosed the relative configuration of **3** (Supplementary Fig. S26), evidencing the spatial correlation of H-4, H-5, H-2 and H_3_-13 upon irradiation of H-5. The spatial correlation between H_3_-12 and H_3_-14 (δ 1.44, s, 3H) was also observed upon irradiation of H_3_-14, which indicated the *cis* stereochemistry between such methyl groups in compound **3** (Fig. 3).

The molecular formula of compound **4** was determined as C_14_H_22_O_3_ by HRMS (ESI-TOF, *m/z*: [M + Na]^+^ calcd for C_14_H_22_O_3_Na 261.1467; found 261.1466). Its ^1^H NMR spectrum (Table 1) revealed the absence of an aldehyde proton, which differs from compounds **1**, **2** and **3**. On the other hand, its ^13^C NMR spectrum (Table 2) presented signals at δ 203.9 (C-1), 170.3 (C-5) and 140.5 (C-9), which indicated the presence of an α,β-unsaturated carbonyl group. Further similarities with compounds **1**–**3** included the presence of four methyl groups in the ^1^H NMR (Table 1) and HSQC (Supplementary Fig. S31) spectra, associated to one doublet (δ 1.10, *J*_10-2_ 7.2 Hz, H_3_-10) and three singlets (δ 1.19/H_3_-13, 1.20/H_3_-12 and 1.32/H_3_-11), in addition to the signals for one oxymethine hydrogen at δ 4.43 (t, *J*_4-3_ 3.0 Hz, H-4), and two doublets for one hydroxymethylene group at δ 3.63 (d, *J*_14a-14b_ 10.8 Hz, 1H, H_a_-14) and 3.42 (d, *J*_14b-14a_ 10.8 Hz, 1H, H_b_-14). The position of the α,β-unsaturated carbonyl group was deduced from HMBC correlations of H_3_-10 to C-1, of H_3_-11 and H_3_-12 to C-5 and of H_b_-14 to C-9 (Fig. 2), which were consistent with the structure of an unprecedented botryane *nor*sesquiterpene carbon skeleton for compound **4**. Its relative configuration was established from NOE experiments (Supplementary Fig. S33). Irradiation of H_3_-10 induced an NOE in H-4, whereas irradiation of H-4 induced an NOE in H_3_-12, indicating a *cis* relative configuration among H_3_-10, H-4 and H_3_-12 (Fig. 3). Conversely, the spatial correlation between H_b_-14 and H_3_-11 was observed upon irradiation of H_b_-14.

Compound **5** was established as a stereoisomer of **4**, based on the same molecular formula, C_14_H_22_O_3_, determined by HRMS (ESI-Q-TOF, *m/z*: [M + H]^+^ calcd for C_14_H_23_O_3_ 239.1642; found 239.1648) in addition to key similarities on their ^1^H and ^13^C NMR (Tables 1 and 2) spectra, except for the resonances assigned to C-2 (δ_H/C_ 2.43/43.5) and C-4 (δ_H/C_ 4.69/68.1), and coupling constants for the H-4 signal (*J*_4-3a_ 4.8, *J*_4-3b_ 10.2). The HMBC spectrum (Supplementary Fig. S39) showed correlations of H_3_-10 (δ 1.10, d, *J*_10-2_ 6.6, 3H) to C-1 (δ 203.4), of H_3_-11 (δ 1.38, s, 3H) and H_3_-12 (δ 1.31, s, 3H) to C-5 (δ 174.6), and of H_b_-14 (δ 3.54, d, *J*_14b-14a_ 10.8, 1H) to C-9 (δ 140.2) (Fig. 2), which are associated with an α,β-unsaturated carbonyl moiety as in compound **4**. Similar results from NOE experiments corroborated the proposed relative configuration for **5** (Supplementary Fig. S40), including the observation of NOEs among H-4, H_3_-12 and H_3_-13 (δ 1.13, s, 3H) upon irradiation of H_3_-12, as well as between H-4 and H-2 (δ 2.43, m), upon irradiation of H-4, which is consistent with a *cis* relative configuration among H-4, H-2, H_3_-12 and H_3_-13. Such data differentiate compound **5** from **4**, which presented a *cis* configuration between H-4 and H_3_-10 (Fig. 3).

It is worth noting the high chemical shift of C-9 for compounds **1**–**3** and for C-5 in case of compounds **4** and **5**, which are consistent with the β-carbon shift of α,β-unsaturated carbonyl moieties^16,17^.

Compound **6** was identified as 4β-Acetoxy-9β,10β,15α-trihydroxyprobotrydial, based on NMR and HRMS data, and comparison with those reported in the literature^18^.

**Figure 4:**
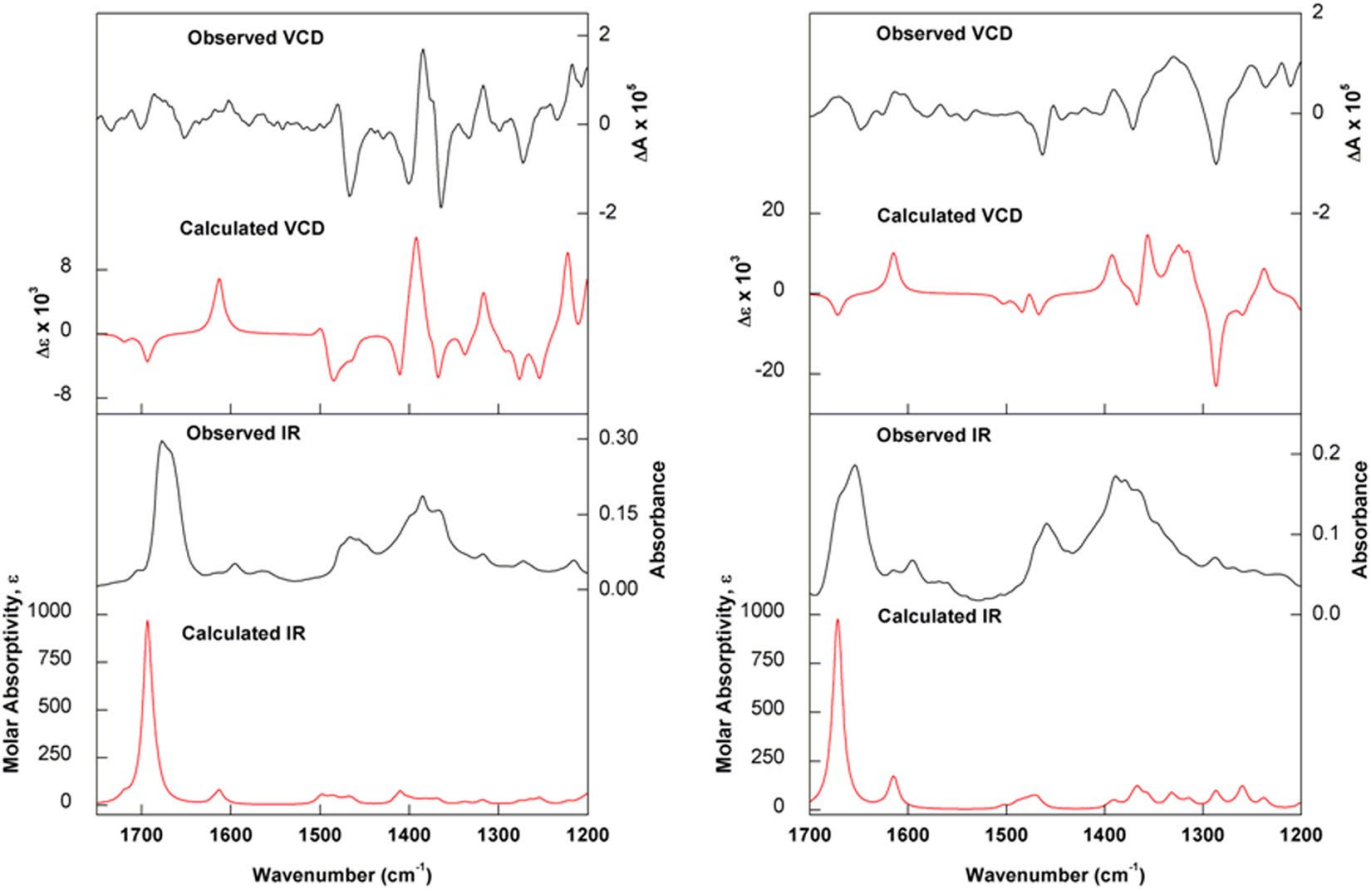
(Left) Experimental and calculated [2*R*,4*S*,5*R*,8*S*; B3LYP/PCM(MeOH)/6-31G(d)] IR and VCD spectra of (+)-**1**. (Right) Experimental and calculated [2*R*,4*S*,8*S*; B3LYP/PCM(MeOH)/6-31G(d)] IR and VCD spectra of (+)-**5**. For structures of the lowest-energy conformers, please see Supplementary Information.

**Figure 5:**
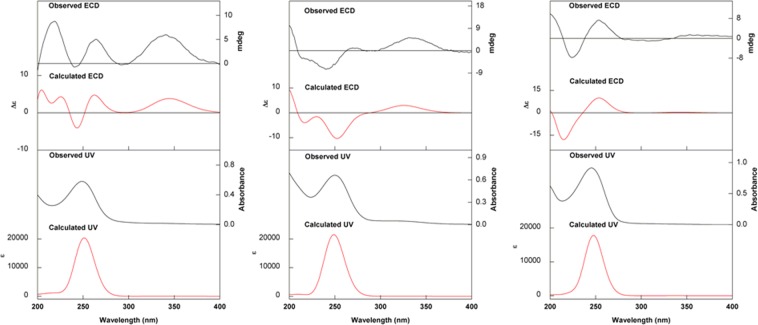
(Left) Experimental and calculated [2*R*,4*R*,5*R*,8*S*; CAM-B3LYP/PCM(MeOH)/TZVP//B3LYP/PCM(MeOH)/6-31G(d)] UV and ECD spectra of (+)-**2** (Center) Experimental and calculated [2*R*,4*S*,5*R*,8*R*; CAM-B3LYP/PCM(MeOH)/TZVP//B3LYP/PCM(MeOH)/6-31G(d)] UV and ECD spectra of (+)-**3** (Right) Experimental and calculated [2*R*,4*R*,8*R*; CAM-B3LYP/PCM(MeOH)/TZVP//B3LYP/PCM(MeOH)/6-31G(d)] UV and ECD spectra of (+)-**4**. For structures of the lowest-energy conformers, please see Supplementary Information.

Due to the amount of sample available, VCD was used for the absolute configuration assignments of compounds **1** and **5** only, while ECD was carried out for compounds **2**–**4**. Regarding compounds **1** and **5**, the similarity between observed and density functional theory (DFT) predicted IR/VCD spectra, at the B3LYP/PCM(MeOH)/6-31G(d) level, led to the unambiguous assignment of their absolute configuration as (+)-(2*R*,4*S*,5*R*,8*S*)-**1** and (+)-(2*R*,4*S*,8*S*)-**5** (Fig. 4). In line with previous results from our group^19^, the agreement between theoretical and observed data in methanol-*d*_4_ solution was greatly improved by considering deuterated hydroxyl groups for the calculations. As for compounds **2**–**4**, the correlation of experimental and time-dependent DFT (TD-DFT) simulated ECD spectra, at the CAM-B3LYP/PCM(MeOH)/TZVP level, allowed the determination of their absolute configuration as (+)-(2*R*,4*R*,5*R*,8*S*)-**2**, (+)-(2*R*,4*S*,5*R*,8*R*)-**3**, and (+)-(2*R*,4*R*,8*R*)-**4** (Fig. 5).

In the specific case of compound **2**, DFT and TD-DFT calculations were carried out for both configurations possible at C-5, as its relative configuration were not readily available from NMR data. As a result, the best agreement between experimental and calculated UV/ECD data was observed for 2*R*,4*R*,5*R*,8*S*-**2** (Fig. 5 and Supplementary Figs S46 and S47).

Compounds **1**–**3** are structurally related to botryenalol, previously isolated from *Botrytis cinerea*, a phytopathogenic ascomycete found in grapes in a Domecq vineyard, Jerez de La Frontera, Cádiz^16^. However, these compounds have different substituents, especially compounds **1** and **2**, which bear a hydroxy group on carbon 5, a structural feature not described yet for botryane terpenoids.

**Figure 6:**
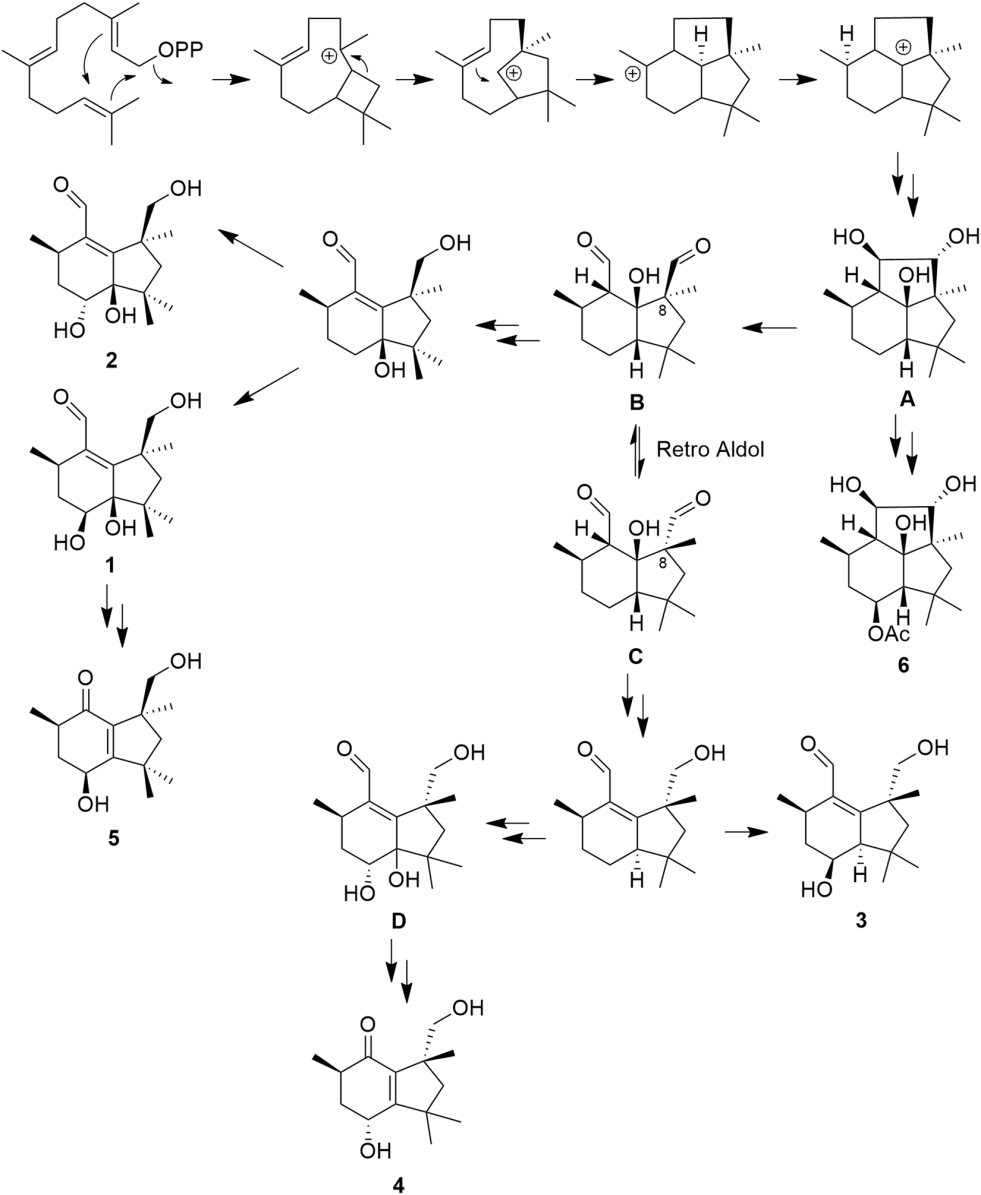
Biogenetic proposal for compounds **1**–**6**.

Biogenetically, botryane derivatives have farnesyldiphosphate (FPP) as a precursor, and the key intermediate **A** is generated after cyclization, rearrangement, and hydroxylation. Subsequent hydroxylation and acetylation reactions could transform **A** to 4β-acetoxy-9β,10β,15α-trihydroxyprobotrydial (**6**), an intermediate in the biosynthesis of botrydial and its derivatives^20,21^. Further steps in the biogenetic proposal include cleavage of the vicinal diol in **A** to give the dialdehyde **B** followed by reduction, dehydration, and hydroxylation to generate **1** and **2**. As suggested by Collado *et al*., a retro Aldol reaction might epimerize C-8 to give intermediate **C**^21^. Reduction, dehydration, and hydroxylation can convert intermediate **C** into **3** and intermediate **D**. Allylic rearrangement and oxidative decarboxylation would convert **1** into **5** and **D** into **4**, the two new *N. bipapillata* metabolites with unprecedented rearranged and degraded terpenoid carbon skeletons (Fig. 6).

Some bothryane derivatives showed phytotoxic effects^18^, as well as antibacterial and cytotoxic activities^13,21,22^. Botryane metabolites were also previously described from marine microorganisms, including a fungal strain of *Geniculosporium* sp. isolated from the red alga *Polysiphonia* sp., which afforded eleven new botryane terpenoids with algicidal, antibacterial and fungicidal activity against *Chlorella fusca*, *Bacillus megaterium* and *Microbotryum violaceum*, respectively^17^. Furthermore, the first botrydiol-coumarin hybrid, hypocrolide A^23^, and novel heterodimeric botryane ethers^24^ were isolated from *Hypocrea* sp., an insect-associated fungus, isolated from a *Septobasidium-*infected insect, *Serrataspis* sp.

The isolated metabolites **1**–**6** were tested for their ability to inhibit cholinesterase (ChEIs) (Table 3). The inhibition assays were carried out using an immobilized capillary enzyme reactor (ICER) based on acetylcholinesterase human recombinant and/or butyrylcholinesterase from human serum, respectively *hu*AChE-ICER and *hu*BChE-ICER (in accordance with published procedure)^25–27^. Compounds **2–6** were more active towards *hu*AChE than *hu*BChE, indicating a selective cholinesterase inhibition, and **4** was the most active compound, with 27.7% (100 μM) inhibition against *hu*AChE. Compound **1** was considered a non-selective inhibitor, as it showed similar inhibitory potentials against both *hu*AChE and *hu*BChE (19.9 and 14.1%, respectively), while its stereoisomer (compound **2**) inhibited only *hu*AChE (18.3%). The results represent only modest inhibition of *hu*AChE or *hu*BChE, but they suggest that botryane terpenoids could act as lead compounds for synthetic development of more potent selective cholinesterase ligands.

**Table 3:** Inhibition of *hu*AChE-ICER and *hu*BChE-ICER activities by galanthamine (positive control; 100 μM) and isolated compounds (100 μM) from *Nemania bipapillata*.

Samples	% inhibition *hu*AChE-ICER ± SEM^a^	% inhibition *hu*BChE-ICER ± SEM^a^
Galanthamine	90.7 ± 0.0	82.0 ± 0.2
**1**	19.9 ± 1.7	14.1 ± 1.7
**2**	18.3 ± 1.8	6.7 ± 0.7
**3**	21.1 ± 0.1	5.5 ± 1.5
**4**	27.7 ± 1.3	7.3 ± 1.5
**5**	22.8 ± 0.8	5.1 ± 0.0
**6**	19.6 ± 2.7	3.2 ± 1.5

^a^Standard error of the mean.

Compounds **1**, **3**, **5** and **6** were tested against colorectal carcinoma HCT-116 and breast adenocarcinoma MCF-7 cell lines using the 3-(4,5-dimethylthiazol-2-yl)-2,5-diphenyltetrazolium bromide tetrazolium reduction (MTT) assay, and presented no significant toxicixity at tested concentrations (IC_50_ > 50 µM) against both cell lines. Previous studies have shown that the presence of the 1,5-dialdehyde moiety, as in botrydial, is an important structural feature for cytotoxicity, including the *S* configuration at C-1^13,21^. Our results corroborate previous findings as compounds **1**, **3**, **5** and **6** do not bear the 1,5-dialdehyde unit, which possibly resulted in the lack of cytotoxic activity. In addition, C-1 in compounds **1**, **3** and **5** is an sp^2^ carbon as part of α,β-unsaturated carbonyl system and therefore the *S* configuration at C-1 requirement is also not met.

In summary, this study expands the known chemodiversity of marine microorganisms, since it is the first to describe the isolation of metabolites from a fungal strain of *Nemania bipapillata*, as well as the occurrence of botryane terpenoids in this genus. Compounds **1**–**5** are new members of the botryane family, highlighting compounds **4** and **5**, which bear a new *nor*sesquiterpene skeleton and were named in accordance with structural functions and the genus *Nemania*. ECD and VCD quantum chemical calculations were essential techniques to determine the absolute configuration of this class of compounds since there were no studies of similar compounds in the literature to be used as models. The cholinesterase inhibitory activities of compounds **1** and **2**, although relatively modest in potency, suggest that they may be lead structures for developing more potent analogs.

## Methods

### General experimental procedure

Optical rotations were determined with a Polartronic H (Schmidt + Haensch) spectrometer. UV spectra were recorded with a Perkin Elmer Lambda 1050 spectrophotometer. ECD and UV spectra were recorded in methanol using a JASCO J-815 spectrometer. Parameters were set as follows: band width 1 nm; response 1 sec; scanning speed 100 nm min^−1^; 3 accumulations; room temperature; 0.1 cm path lentgh cell; concentration 0.3–0.5 mg mL^−1^. IR and VCD spectra of compounds **1** and **5** were measured simultaneously with a Chiral*IR*-2X FT-VCD spectrometer (BioTools) equipped with a single photoelastic modulator (PEM) at a resolution of 4 cm^−1^ for 8 h. The optimum retardation of the ZnSe PEM was set at 1400 cm^−1^. Baselines were corrected by subtracting the VCD spectrum of compounds **1** and **5** from that obtained for the solvent. Vibrational chiroptical spectra were measured in a BaF_2_ cell with 100 μm path length in methanol-*d*_4_. Samples concentration: compound **1**, 4 mg/120 μL of CD_3_OD, compound **5**, 3 mg/120 μL of CD_3_OD. 1D and 2D NMR experiments were recorded on Bruker Avance III HD 600 and Bruker Avance 600 (14,1 T) spectrometers with a 5 mm TCI cryoprobe. Chemical shifts were reported in δ (ppm) and were referenced to the residual DMSO-*d*_6_ and CD_3_OD (δ 2.49 and 3.31, respectively, for ^1^H chemical shifts; δ 39.5 and 49.0 for ^13^C chemical shifts). High resolution ESI-TOF and ESI-Q-TOF-MS were recorded with Waters/Micromass LCT and Bruker Maxix Impact spectrometers, respectively. Semi-preparative HPLC was performed on a Shimadzu equipment containing two pumps LC-6AD, a SPD-M20A photodiode array detector and a system controller CBM-20A, and using a C-18 (2) Phenomenex Luna (250 × 10 mm; 5 μm; 100 Å) column and HPLC grade solvents. Chromatography analyses were carried out using reversed phase silica column (C-18, 40–75 μm, 60 Å; Sorbent Technologies). Silica gel plates on aluminum (silica gel 60 F_254_ -Merck) were used for analytical thin layer chromatography (TLC).

### Collection of alga

*Asparagopsis taxiformis* (*Falkenbergia* stage) specimens were collected at the rocky shore of Fortaleza Beach (Ubatuba, SP, Brazil, GPS: 23°21′38″S, 44°49′63″W), in June 2013. A voucher specimen (SP 428533) was deposited in the herbarium of the Institute of Botany (SMA/SP). After washing carefully with seawater, the algal specimens were kept in previously prepared flasks containing sterile seawater and antibiotic chloramphenicol (200 mg L^−1^).

### Fungal material

Algal specimens were immersed in a 1% NaOCl solution (v/v) during 6 seconds and 70% ethanol solution (v/v) during 2 seconds, followed by seawater rinsing as a surface sterilization procedure. Then, each alga was fragmented and small pieces were spread on PDA (potato, dextrose and agar) plates prepared with sterilized seawater containing the antibiotic chloramphenicol (200 mg L^−1^). A fungal strain codified as AT-05 was isolated after successive replication in PDA plates. Total DNA was extracted using fresh mycelium with an extraction kit (ZR Fungal/Bacterial DNA MicroPrep) following the manufacturer’s recommendations. Molecular identification was carried out by sequencing of ITS (primers ITS1 and ITS4)^28^. The amplicons were enzymatically purified (Agencourt AMPure XP, Beckman Coulter Inc.) and sequenced by the Sanger method (BigDye Terminator v.3.1 Cycle Sequencing). The obtained sequences were uploaded to Genbank database^29^ (accession number MH025760) and the comparison with sequences from the GenBank database was carried out using the nBLAST tool. The fungal strain was identified as *Nemania bipapillata*^30^. Voucher specimen was deposited at the Endophytic Fungi Collection in our laboratory.

### Fermentation, extraction and isolation

Small agar slices bearing mycelia of the AT-05 strain were transferred to 15 Erlenmeyer flasks (500 mL) containing 300 mL of PDB (potato, dextrose broth) medium prepared with deionized water and incubated for 28 days at 25 °C. Thereafter, the medium was separated from the mycelium by filtration and extracted with EtOAc (3 × 50% of each medium volume). Evaporation of EtOAc yielded 1.74 g of AT-05 crude extract.

An aliquot of AT-05 crude extract (1.51 g) was fractionated on a C-18 derivatized silica column (150 g, Φ = 3.5 cm) using MeOH:H_2_O gradient (1:1, 60:40, 75:25, 100:0) as eluent and yielded 4 fractions (360 mL each; AT-05-F1 - AT-05-F4). Fraction AT-05-F2 (200 mg) was chromatographed by semi-preparative HPLC using a linear gradient (30 to 80% MeOH:H_2_O) for 40 minutes and a flow rate of 4.5 mL min^−1^, which afforded compounds **1** (t_R_ 23.5 min; 5.8 mg), **2** (t_R_ 21.1 min; 1.0 mg), **3** (t_R_ 25.0 min; 3.0 mg), **4** (t_R_ 21.9 min; 1.4 mg), **5** (t_R_ 27.3 min; 3.6 mg) and **6** (t_R_ 26.4 min; 13.8 mg).

(+)-(2*R*,4*S*,5*R*,8*S*)-4-deacetyl-5-hydroxy-botryenalol (**1**): colorless oil; [α]_D_^23^ + 55.9 (*c* 0.2, MeOH); UV (MeOH) λ_max_ (log ε) 248 nm (3.80); CD (MeOH, 1.86 mmol L^−1^) λ_ext_ (Δε) 337 (+2.11), 246 (−2.87), 206 nm (+4.59); ^1^H NMR data see Table 1; ^13^C NMR data see Table 2; HRMS (ESI-TOF) *m/z*: [M + Na]^+^ calcd for C_15_H_24_O_4_Na 291.1572; found 291.1576.

(+)-(2*R*,4*R*,5*R*,8*S*)-4-deacetyl-5-hydroxy-botryenalol (**2**): colorless oil; [α]_D_^23^ + 33.7 (*c* 0.13, MeOH); UV (MeOH) λ_max_ (log ε) 249 nm (3.01); CD (MeOH, 1.86 mmol L^−1^) λ_ext_ (Δε) 341 (+0.97), 264 (+0.82), 220 nm (+1.43); ^1^H NMR data: see Table 1; ^13^C NMR data: see Table 2; HRMS (ESI-Q-TOF) *m/z*: [M + Na]^+^ calcd for C_15_H_24_O_4_Na 291.1572; found 291.1563.

(+)-(2*R*,4*S*,5*R*,8*R*)-4-deacetyl-botryenalol (**3**): colorless oil; [α]_D_^23^ + 22.2 (*c* 0.2, MeOH); UV (MeOH) λ_max_ (log ε) 250 nm (3.49); CD (MeOH, 1.98 mmol L^−1^) λ_ext_ (Δε) 330 (+0.91), 270 (+0.29), 237 nm (–0.93); ^1^H NMR data see Table 1; ^13^C NMR data see Table 2; HRMS (ESI-Q-TOF) *m/z*: [M + Na]^+^ calcd for C_15_H_24_O_3_Na 275.1623; found 275.1613.

Nemenonediol A [(+)-(2*R*,4*R*,8*R*)-**4**]: colorless oil; [α]_D_^23^ + 6.86 (*c* 0.14, MeOH); UV (MeOH) λ_max_ (log ε) 246 nm (3.77); CD (MeOH, 1.26 mmol L^−1^) λ_ext_ (Δε) 309 (– 0.40), 253 (+1.67), 224 (– 1.93); ^1^H NMR data see Table 1; ^13^C NMR data see Table 2; HRMS (ESI-TOF) *m/z*: [M + Na]^+^ calcd for C_14_H_22_O_3_Na 261.1467; found 261.1466. IUPAC nomenclature: (3*R*,5*R*,7*R*)-7-hydroxy-3-(hydroxymethyl)-1,1,3,5-tetramethyl-2,3,6,7-tetrahydro-1H-inden-4(5H)-one.

Nemenonediol B [(+)-(2*R*,4*S*,8*S*)-**5**]: colorless oil; [α]_D_^23^ + 8.6 (*c* 0.2, MeOH); UV (MeOH) λ_max_ (log ε) 247 nm (3.65); CD (MeOH, 2.10 mmol L^−1^) λ_ext_ (Δε) 251 (– 4.06), 215 nm (+6.09); ^1^H NMR data see Table 1; ^13^C NMR data see Table 2; HRMS (ESI-Q-TOF) *m/z*: [M + H]^+^ calcd for C_14_H_23_O_3_ 239.1642; found 239.1648. IUPAC nomenclature: (3*S*,5*R*,7*S*)-7-hydroxy-3-(hydroxymethyl)-1,1,3,5-tetramethyl-2,3,6,7-tetrahydro-1H-inden-4(5H)-one.

4β-Acetoxy-9β,10β,15α-trihydroxyprobotrydial (**6**): colorless oil; ^1^H NMR and ^13^C NMR data were consistent with those previously reported^18^; HRMS (ESI-Q-TOF) *m/z*: [M + Na]^+^ calcd for C_17_H_28_O_5_Na 335.1834; found 335.1828.

### Calculations

This section was adapted from previously published methods^19,31^. All DFT simulations were carried using Gaussian 09 software^32^. Solvation in methanol was treated implicitly using the polarizable continuum model (PCM) in its integral equation formalism version (IEFPCM). For the calculations, the following configurations were arbitrarily chosen: (2*R*,4*S*,5*R*,8*S*)-**1**; (2*R*,4*R*,5*S*,8*S*)- and (2*R*,4*R*,5*R*,8*S*)-**2**; (2*R*,4*S*,5*R*,8*R*)-**3**; (2*R*,4*R*,8*R*)-**4**; and (2*R*,4*S*,8*S*)-**5**. Conformational searches were performed using molecular mechanics with the Monte Carlo algorithm and MM + force field available in HyperChem 8.0.10 software package. For (2*R*,4*S*,5*R*,8*S*)-**1**, 10 conformers with relative energy (rel E.) within 6 kcal mol^−1^ were selected and had their geometry optimized at the B3LYP/PCM(MeOH)/6-31G* level. The five conformers with rel E. <2.0 kcal mol^−1^ (>98% of the total Boltzmann distribution) were selected for IR and VCD spectral calculations. Regarding (2*R*,4*R*,5*S*,8*S*)- and (2*R*,4*R*,5*R*,8*S*)-**2**, 2 conformers each, with rel E. within 6 kcal mol^−1^ were selected and had their geometry optimized at the B3LYP/PCM(MeOH)/6-31 G* level. One and two conformers, respectively (rel E. <2.0 kcal mol^−1^) were used for UV and ECD spectral calculations. For (2*R*,4*S*,5*R*,8*R*)-**3**, 8 conformers (rel E. within 6 kcal mol^−1^) were selected and had their geometry optimized at the B3LYP/PCM(MeOH)/6-31G* level. Two conformers with rel E. <2.0 kcal mol^−1^ were used during UV and ECD spectral calculations. In the case of (2*R*,4*R*,8*R*)-**4**, 2 conformers (rel E. within 6 kcal mol^−1^) were selected and had their geometry optimized at the B3LYP/PCM(MeOH)/6-31G* level. These two conformations (rel E. <2.0 kcal mol^−1^) were selected for UV and ECD spectral calculations. For (2*R*,4*S*,8*S*)-**5**, 2 conformations (rel E. within 6 kcal mol^−1^) were selected and had their geometry optimized at the B3LYP/PCM(MeOH)/6-31G* level. These same conformers were used for IR and VCD calculations. Replacement of some hydrogen atoms with deuterium atoms was performed using GaussView 5.0.9 software for the same conformer population identified for each configuration. Dipole and rotational strengths were used to create IR and VCD spectra, in M^−1^ cm^−1^ units, These quantities were calculated at the same level of theory used during geometry optimization. Spectra were plotted as a sum of Lorentzian bands with HWHM of 6 cm^−1^. The calculated wavenumbers were multiplied with a scaling factor of 0.975. The final spectra were generated as Boltzmann averages of the lowest-energy conformers identified and plotted using Origin8 software. No imaginary frequencies were obtained after vibrational analysis at the B3LYP/PCM(MeOH)/6-31G* level thus confirming the considered conformers as real minima. Subsequently, TD-DFT was used to calculate excitation energies (in nm) and rotatory strengths (*R*) in dipole velocity form (*R*_vel_ in cgs units: 10^−40^ esu^2^ cm^2^), at the CAM-B3LYP/PCM(MeOH)/TZVP level. The calculated rotatory strengths from the first 30 singlet → singlet electronic transitions were simulated into an ECD curve using Gaussian bands with bandwidth σ 0.25 eV. Calculated wavelength transitions were multiplied with a scaling factor of 1.04, which was determined based on the agreement between experimental and calculated UV data. The final spectra were generated as simple averages of the lowest-energy conformers and plotted using Origin8 software.

### Cholinesterase inhibition screening assays

Test samples were submitted to a punctual cholinesterase inhibition screening assay. *hu*AChE and *hu*BChE were immobilized independently onto fused silica capillary (0.1 mm I.D × 0.375 mm × 30 cm) using glutaraldehyde as spacer (*hu*AChE-ICER and *hu*BChE-ICER)^26,27^.

*hu*AChE-ICER and *hu*BChE-ICER were interfaced to the LC–IT-MS/MS as alow affinity and high selectivity biochromatography column. The LC system (Nexera, Shimadzu) consisted of two LC-20AD pumps, a SIL20A autosampler with a 50 μL loop, a DGU-20A5 degasser and a CBM-20A interface. The LC system was coupled to an Amazon Speed Ion Trap (IT) mass spectrometer (Bruker Daltonics) equipped with an ESI source, operating in a positive mode (scan 50–250 *m/z*). Data acquisition was carried out using the Bruker Data Analysis Software (version 4.3). All analyses were performed at room temperature (21 °C). The enzymatic reaction was monitored by direct quantification of acetylcholine hydrolysis product, Ch (*m/z*: [M + H]^+^ 104.17)^26,27^.

2 U mL^−1^ of *hu*AChE and 0.5 U mL^−1^ of *hu*BChE were immobilized and the apparent kinetic constant (K_*Mapp*_ = 70 μM) was obtained independently using acetylcholine (ACh) as substrate by varying the substrate concentration while measuring the Ch [M + H]^+^ 104.17 *m/z* formation. GraphPad Prism 5 software was used to obtain Michaelis–Menten plots by nonlinear regression analysis^27^.

Ammonium acetate solution (15 mM, pH 8.0) was used as running buffer at a flow rate of 50 μL min^−1^and a second pump delivered methanol after the *hu*BChE-ICER or *hu*AChE-ICER and before the IT-MS throughout a “T” shaped connection, at the same flow rate as the running buffer. Total analysis time was 3 min. IT-MS parameters were: 4.000 V capillary voltage, 500 V end plate voltage, 8.0 Lmin^−1^ drying gas, 275 °C drying temperature and 35 psi nebulizer. Isolation width was set at ±*m/z* 0.5. Fragmentation amplitude was set to 120%. Nitrogen was used as sheath gas and helium as collision gas^27^.

Galanthamine was used as standard cholinesterase inhibitor. Methanol stock solutions (1 mM) were prepared for each tested compound. Reaction mixtures (100 μL) were prepared by mixing 100 μM of tested compound with ACh 70 μM. Final volume was completed with ammonium acetate solution (15 mM, pH 8.0). Each reaction mixture (10 μL) was injected into the LC-MS system and the percentage of inhibition was calculated in accordance with Eq. 1.

1 $$ \% \,{\rm{inhibition}}=[{\rm{1}}-\frac{{\rm{Pi}}}{{P}_{0}}]\times {\rm{100}}$$

where, P is the attained peak area of Ch produced: (i) in the presence of the tested compound; (0) in the absence of the tested compound^25–27^.

Any possible interference at the 104 *m/z* range from the tested sample was evaluated injecting a reaction mixture without the substrate.

### Cytotoxic activity

The cytotoxicity of compounds **1**, **3**, **5** and **6** was measured through the MTT (3-(4,5-dimethyl-2-thiazolyl)-2,5-diphenyl-2H-tetrazolium bromide) assay^33^, that is based in the conversion of the tetrazolium salt to a formazan product by viable cells. Two cell lines obtained from American Type Culture Collection (Manassas, Virginia, EUA) were used: HCT-116 (colorectal carcinoma, ATCC^®^ CCL-247™) and MCF-7 (breast adenocarcinoma, ATCC^®^ HTB-22). The assay was conducted essentially as described by Monteiro *et al*.^34^. The HCT-116 and MCF-7 cells (1 × 10^4^ cells well^−1^) were seeded in 96-well plates and, after 24 h, different concentrations (5 µM and 50 µM) of the compounds were added and incubated for 72 h. Doxorubicin (0.003 to 10 µM) and DMSO (0.5%) were used as a positive and negative controls, respectively. At the end of incubation, the supernatant was replaced with fresh medium containing MTT (0.5 mg/mL) for 3 h. The supernatant was, then, removed, and the MTT formazan product was dissolved in 150 μL DMSO. The absorbance was measured using a multiplate reader (Multiskan™ FC, Thermo Scientific, Finland) at 570 nm. All experiments were performed in duplicate. IC_50_ values, along with 95% confidence intervals, were calculated by non-linear regression using GraphPad Prism 5.0 (Intuitive Software for Science, La Jolla, CA, USA).

## Supplementary information

Supplementary Information,

## Data Availability

The authors declare availability to all data, materials or information contained in this manuscript.
