# Supplementary material for: Botryane terpenoids produced by Nemania bipapillata, an endophytic fungus isolated from red alga Asparagopsis taxiformis - Falkenbergia stage
Source: Sci Rep. 2019 Aug 23;9:12318. doi: 10.1038/s41598-019-48655-7 (PMC6707159; doi:10.1038/s41598-019-48655-7)
Supplement: Supplementary file 1 — Supplementary Information, [file 41598_2019_48655_MOESM1_ESM.pdf]

Botryane terpenoids produced by *Nemania bipapillata*, an endophytic fungus isolated from red alga *Asparagopsis taxiformis - Falkenbergia* stage.

Rebeca P. Medina[1], Angela R. Araújo[1], João M. Batista Jr[2,3], Carmen L. Cardoso[4], Cláudia Seidl[4], Adriana F. L. Vilela[4], Helori V. Domingos[5], Leticia V. Costa-Lotufo[5], Raymond J. Andersen[6], Dulce H. S. Silva[1*]

[1]Núcleo de Bioensaios, Biossíntese e Ecofisiologia de Produtos Naturais (NuBBE), Departamento de Química Orgânica, Instituto de Química, UNESP - Universidade Estadual Paulista, 14801-970, Araraquara-SP, Brazil.
[2]Departamento de Química, Centro de Ciências Exatas e de Tecnologia, Universidade Federal de São Carlos - UFSCar, 13565-905, São Carlos-SP, Brazil.
[3]Departamento de Ciência e Tecnologia, Universidade Federal de São Paulo – UNIFESP, 12231-280, São José dos Campos-SP, Brazil
[4]Grupo de Cromatografia de Bioafinidade e Produtos Naturais, Departamento de Química, Faculdade de Filosofia, Ciências e Letras de Ribeirão Preto, Universidade de São Paulo, 14040-901, Ribeirão Preto-SP, Brazil.
[5]Instituto de Ciências Biomédicas, Universidade de São Paulo, 05508-900, São Paulo-SP, Brazil.
[6]Departments of Chemistry and Earth, Ocean & Atmospheric Sciences, University of British Columbia, V6T 1Z1, Vancouver, BC, Canada.

**Corresponding author: dulce.silva@unesp.br*

TABLE OF CONTENTS

Figure S1 - High resolution ESI-TOF-MS spectrum of compound **1** (Waters/Micromass LCT)........ **6**
Figure S2 - ^{1}H NMR spectrum of compound **1** (CD_3OD; 600 MHz)........ **7**
Figura S3 - ^{13}C NMR spectrum of compound **1** (CD_3OD; 150 MHz)........ **8**
Figure S4 - ^{1}H–^{1}H COSY spectrum of compound **1** (CD_3OD; 600 MHz)........ **9**
Figure S5 - HSQC spectrum of compound **1** (CD_3OD; 600 and 150 MHz)........ **10**
Figure S6 - HMBC spectrum of compound **1** (CD_3OD; 600 and 150 MHz)........ **11**
Table S1. ^{1}H and ^{13}C NMR data for Compound **1** in DMSO - d_6........ **12**
Figure S7 - ^{1}H NMR spectrum of compound **1** (DMSO-d_6; 600 MHz)........ **13**
Figura S8 - ^{13}C NMR spectrum of compound **1** (DMSO-d_6; 150 MHz)........ **14**
Figure S9 - ROESY spectrum of compound **1** (DMSO-d_6; 600 MHz)........ **15**
Figure S10 - CD spectrum of compound **1**........ **16**
Figure S11 - High resolution ESI-Q-TOF-MS spectrum of compound **2** (Bruker - MaXix Impact)........ **17**
Figure S12 - ^{1}H NMR spectrum of compound **2** (CD_3OD; 600 MHz)........ **18**
Figura S13 - ^{13}C NMR spectrum of compound **2** (CD_3OD; 150 MHz)........ **19**
Figure S14 - ^{1}H–^{1}H COSY spectrum of compound **2** (CD_3OD; 600 MHz)........ **20**
Figure S15 - HSQC spectrum of compound **2** (CD_3OD; 600 and 150 MHz)........ **21**
Figure S16 - HMBC spectrum of compound **2** (CD_3OD; 600 and 150 MHz)........ **22**
Figure S17 - ^{1}H NMR (blue) and NOESY 1D (green - selected signal: δ_H 4.10; red - selected signal: δ_H 1.48) spectra of compound **2** - (CD_3OD; 600 MHz)........ **23**
Figure S18 - High resolution ESI-Q-TOF-MS spectrum of compound **3** (Bruker - MaXix Impact)........ **24**
Figure S19 - ^{1}H NMR spectrum of compound **3** (CD_3OD; 600 MHz)........ **25**
Figura S20 - ^{13}C NMR spectrum of compound **3** (CD_3OD; 150 MHz)........ **26**
Figure S21 - ^{1}H–^{1}H COSY spectrum of compound **3** (CD_3OD; 600 MHz)........ **27**
Figure S22 - HSQC spectrum of compound **3** (CD_3OD; 600 and 150 MHz)........ **28**
Figure S23 - HOMODEC experiment upon irradiation of signal δ_H 2.79 (H-2) of compound **3** - (CD_3OD; 600 MHz)........ **29**
Figure S24 - HMBC spectrum of compound **3** (CD_3OD; 600 and 150 MHz)........ **30**

Figure S25 - HMBC spectrum expansion of compound **3** (CD_3OD; 600 and 150 MHz)........ **31**

Figure S26 - ^1H NMR (blue) and NOESY 1D (green - selected signal: δ_H 2.33; red - selected signal: δ_H 1.44) spectra of compound **3** - (CD_3OD; 600 MHz)............ **32**

Figure S27 - High resolution ESI-TOF-MS spectrum of compound **4** (Waters/Micromass LCT)........ **33**

Figure S28 - ^1H NMR spectrum of compound **4** (CD_3OD; 600 MHz)...................... **34**

Figure S29 - ^{13}C NMR spectrum of compound **4** (CD_3OD; 150 MHz).................... **35**

Figure S30 - ^1H–^1H COSY spectrum of compound **4** (CD_3OD; 600 MHz).............. **36**

Figure S31 - HSQC spectrum of compound **4** (CD_3OD; 600 and 150 MHz)........... **37**

Figure S32 - HMBC spectrum of compound **4** (CD_3OD; 600 and 150 MHz).......... **38**

Figure S33- ^1H NMR (purple) and NOESY 1D (light blue - selected signal: δ_H 1.10; green - selected signal: δ_H 4.43; red - selected signal: δ_H 3.42) spectra of compound **4** - (CD_3OD; 600 MHz)........ **39**

Figure S34 - High resolution ESI-Q-TOF-MS spectrum of compound **5** (Bruker - MaXix Impact)........ **40**

Figure S35 - ^1H NMR spectrum of compound **5** (CD_3OD; 600 MHz)...................... **41**

Figura S36 - ^{13}C NMR spectrum of compound **5** (CD_3OD; 150 MHz).................... **42**

Figure S37 - ^1H–^1H COSY spectrum of compound **5** (CD_3OD; 600 MHz).............. **43**

Figure S38 - HSQC spectrum of compound **5** (CD_3OD; 600 and 150 MHz)........... **44**

Figure S39 - HMBC spectrum of compound **5** (CD_3OD; 600 and 150 MHz).......... **45**

Figure S40 - ^1H NMR (blue) and NOESY 1D (green - selected signal: δ_H1.31; red - selected signal: δ_H 4.69) spectra of compound **5** - (CD_3OD; 600 MHz)................. **46**

Figure S41 - CD spectrum of compound **5**........ **47**

Figure S42 - High resolution ESI-Q-TOF-MS spectrum of compound **6** (Bruker - MaXix Impact)........ **48**

Figure S43 - ^1H NMR spectrum of compound **6** (CD_3OD; 600 MHz)...................... **49**

Figure S44 - ^{13}C NMR spectrum of compound **6** (CD_3OD; 600 MHz).................... **50**

Figure S45 - Structures, relative energies and Boltzmann factors of the lowest-energy conformers identified for (2*R*,4*S*,5*R*,8*S*)-**1** at the B3LYP/PCM(MeOH)/6-31G(d) level........ **51**

Figure S46 - Structures of the lowest-energy conformers identified for (2*R*,4*R*,5*R*,8*S*)-**2** at the B3LYP/PCM(MeOH)/6-31G(d) level and used as simple average.. **52**
Figure S47 - Structure of the lowest-energy conformer identified for (2*R*,4*R*,5*S*,8*S*)-**2** at the B3LYP/PCM(MeOH)/6-31G(d) and its UV and ECD spectra calculated at the CAM-B3LYP/PCM(MeOH)/TZVP level.......................... **53**
Figure S48 - Structures of the lowest-energy conformers identified for (2*R*,4*S*,5*R*,8*R*)-**3** at the B3LYP/PCM(MeOH)/6-31G(d) level and used as simple average.. **54**
Figure S49 - Structures of the lowest-energy conformers identified for (2*R*,4*R*,8*R*)-**4** at the B3LYP/PCM(MeOH)/6-31G(d) level and used as simple average.. **54**
Figure S50 - Structures, relative energies and Boltzmann factors of the lowest-energy conformers identified for (2*R*,4*S*,8*S*)-**5** at the B3LYP/PCM(MeOH)/6-31G(d) level.. **55**
Table S2 - Cartesian coordinates of lowest-energy conformers (compound **1** - conformer 1).. **56**
Table S3 - Cartesian coordinates of lowest-energy conformers (compound **1** - conformer 2).. **57**
Table S4 - Cartesian coordinates of lowest-energy conformers (compound **1** - conformer 3).. **58**
Table S5 - Cartesian coordinates of lowest-energy conformers (compound **1** - conformer 4).. **59**
Table S6 - Cartesian coordinates of lowest-energy conformers (compound **1** - conformer 5).. **60**
Table S7 - Cartesian coordinates of lowest-energy conformers (compound **2** - conformer 1).. **61**
Table S8 - Cartesian coordinates of lowest-energy conformers (compound **2** - conformer 2).. **62**
Table S9 - Cartesian coordinates of lowest-energy conformers (compound **3** - conformer 1).. **63**
Table S10 - Cartesian coordinates of lowest-energy conformers (compound **3** - conformer 2).. **64**

Table S11 - Cartesian coordinates of lowest-energy conformers (compound **4** - conformer 1).. **65**
Table S12 - Cartesian coordinates of lowest-energy conformers (compound **4** - conformer 2).. **66**
Table S13 - Cartesian coordinates of lowest-energy conformers (compound **5** - conformer 1).. **67**
Table S14 - Cartesian coordinates of lowest-energy conformers (compound **5** - conformer 2).. **68**

Elemental Composition Report

Page 1

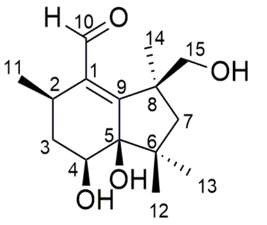

1

Single Mass Analysis

Tolerance = 5.0 PPM / DBE: min = -3.0, max = 100.0
Element prediction: Off
Number of isotope peaks used for i-FIT = 3

Monoisotopic Mass, Even Electron Ions
735 formula(e) evaluated with 3 results within limits (all results (up to 1000) for each mass)
Elements Used:
C: 0-60 H: 0-80 N: 0-10 O: 0-10 Na: 0-1
PMI-7
EH9281 384 (7.045) AM (Cen,4, 50.00, Ar,5000.0,303.21,1.00); Sm (SG, 1x1.00); Sb (1,40.00); Cm (358:403)

16-Sep-2015
TOF MS ES+
1.28e+005

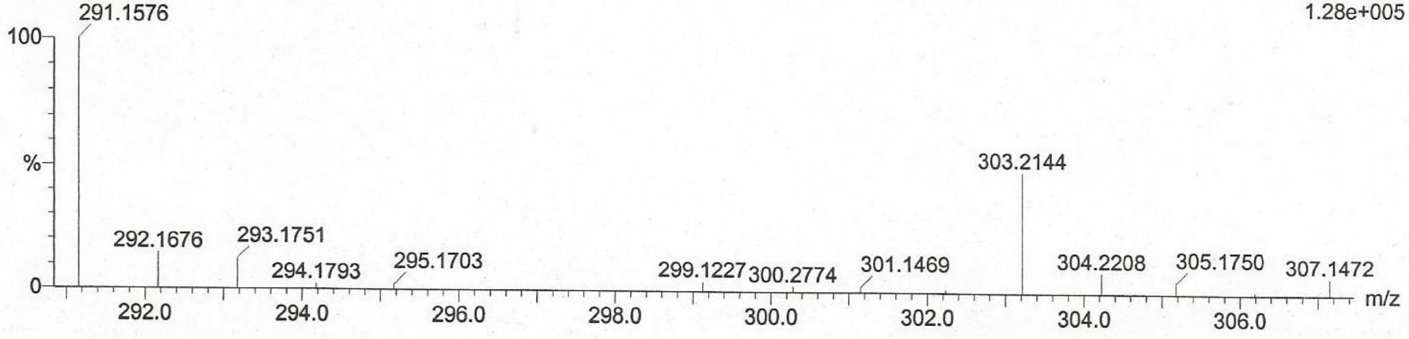

Minimum:				-3.0		
Maximum:		5.0	5.0	100.0		
Mass	Calc. Mass	mDa	PPM	DBE	i-FIT	Formula
291.1576	291.1572	0.4	1.4	3.5	5498.7	C15 H24 O4 Na
	291.1569	0.7	2.4	7.5	5927.3	C13 H19 N6 O2
	291.1586	-1.0	-3.4	8.5	6602.4	C16 H20 N4 Na

Figure S1 - High resolution ESI-TOF-MS spectrum of compound **1**(Waters/Micromass LCT)

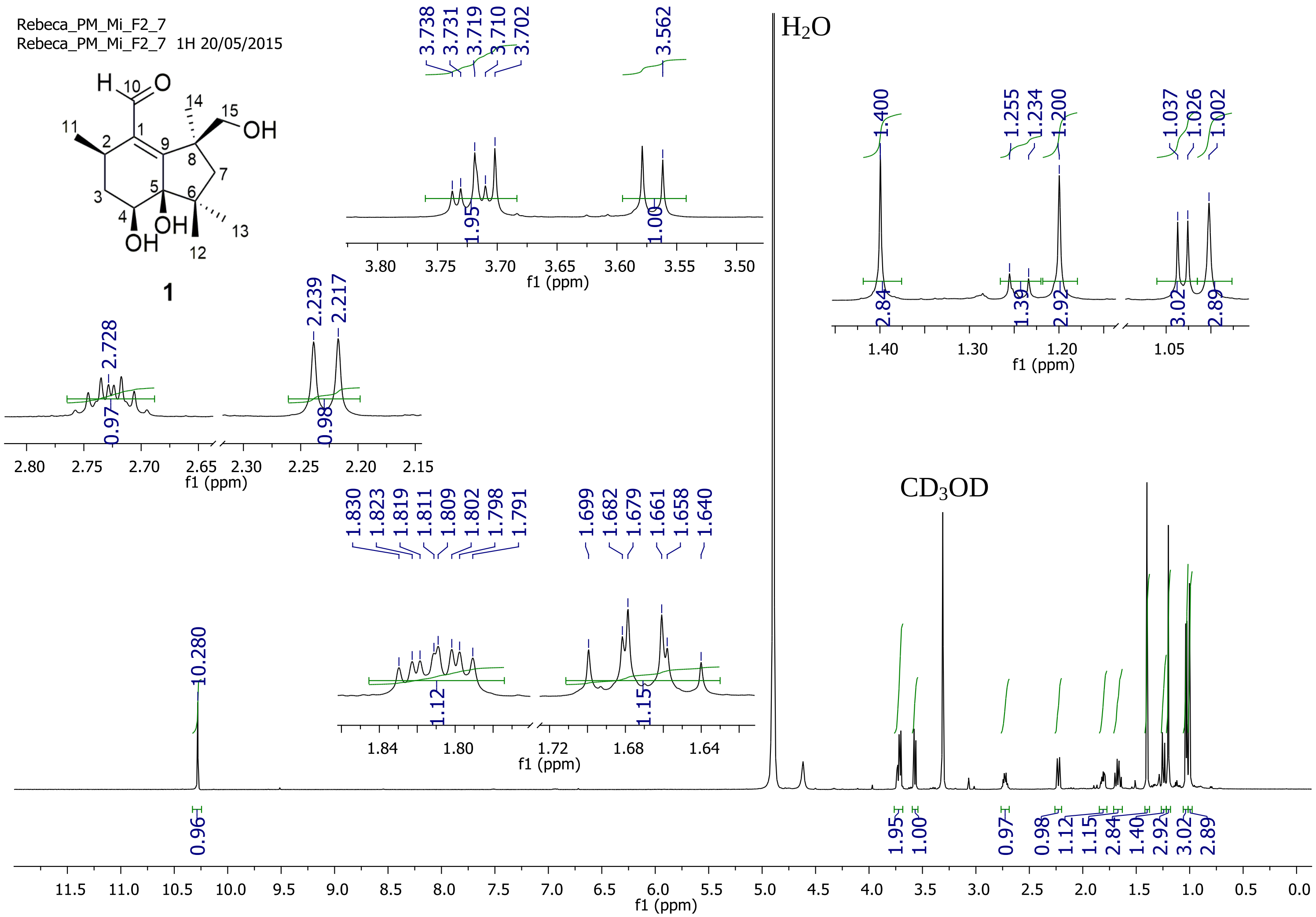

Figure S2 - ^{1}H NMR spectrum of compound **1** (CD_3OD; 600 MHz)

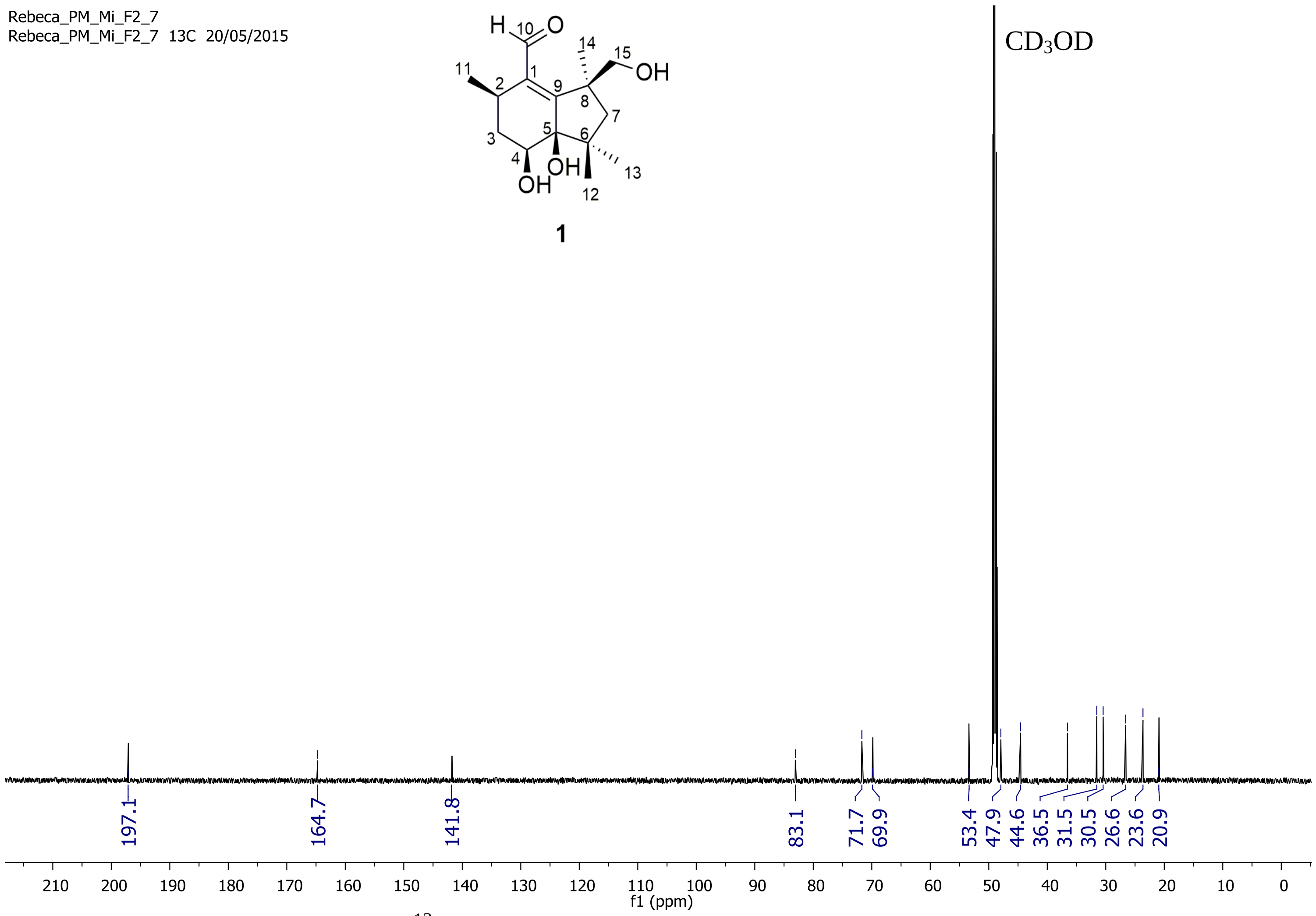

Figura S3 - ^{13}C NMR spectrum of compound **1** (CD_3OD; 150 MHz)

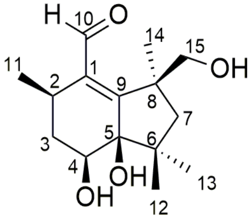

1

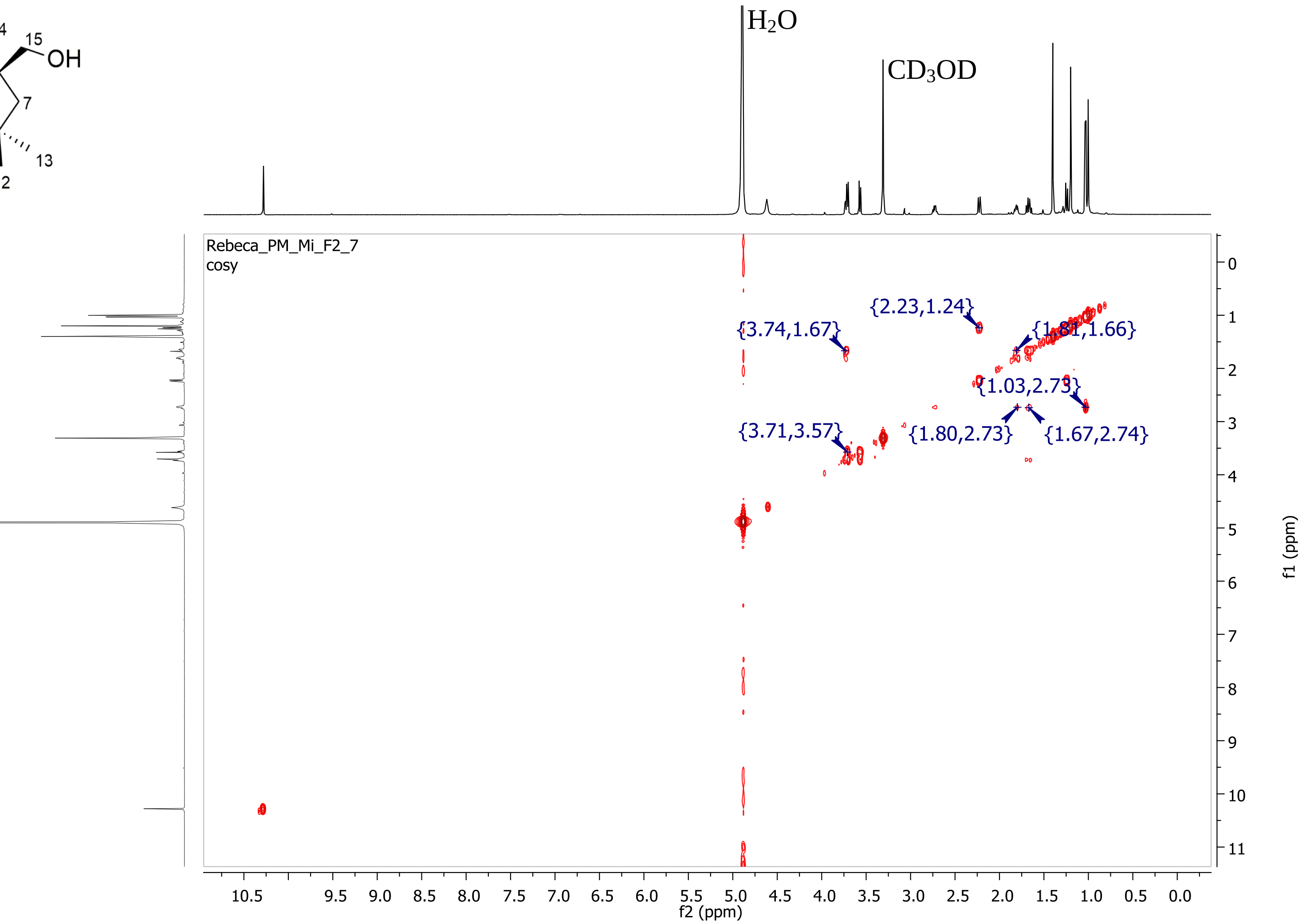

Figure S4 - ^{1}H–^{1}HCOSY spectrum of compound **1** (CD_3OD; 600 MHz)

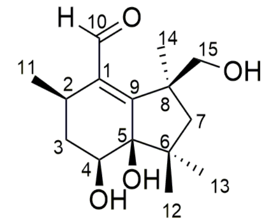

1

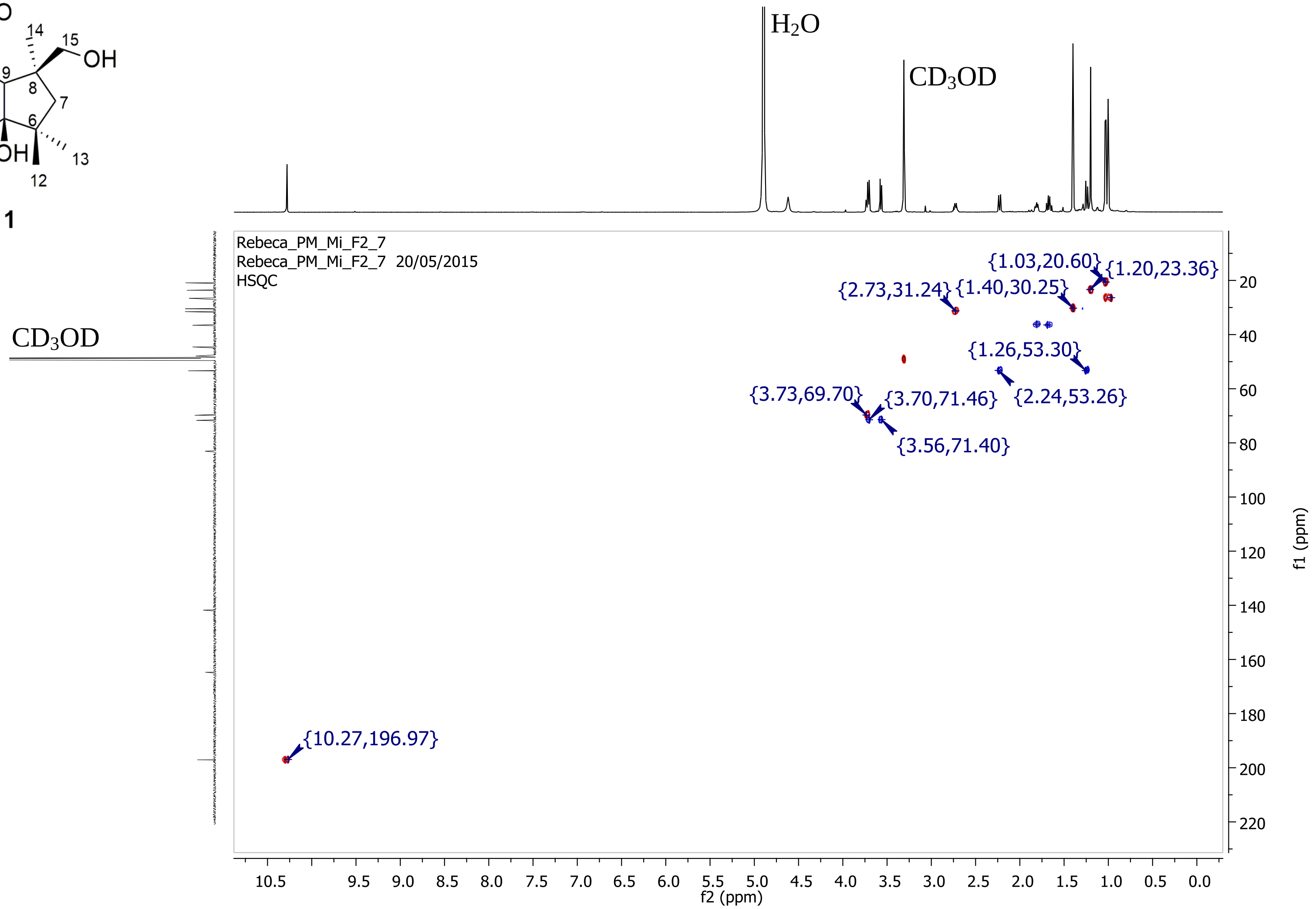

Figure S5 - HSQC spectrum of compound **1** (CD_3OD; 600 and 150 MHz)

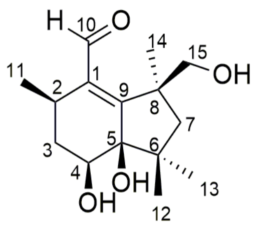

1

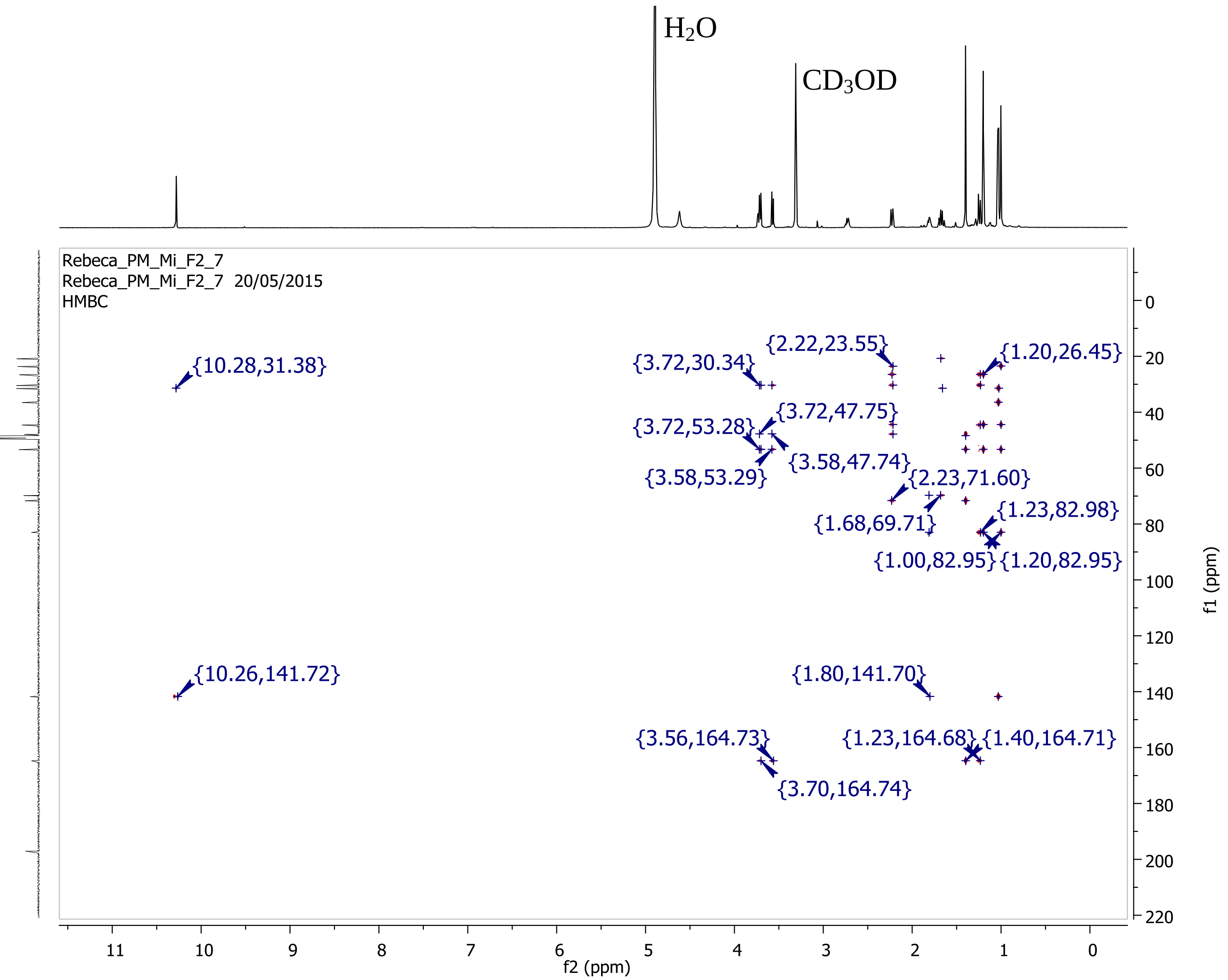

Figure S6 - HMBC spectrum of compound **1** (CD_3OD; 600 and 150 MHz)

Table S1. ^{1}H and ^{13}C NMR data for Compound **1** in DMSO - d_6.

**Position**	**δ_H (J in Hz)**	**δ_C**
1		139.2
2	2.60 (m)	29.6
3	1.66 (ddd, 4.2, 6.9, 11.7)	35.3
	1.54 (m)	
4	3.53 (ddd, 4.5, 7.5, 12.3)	67.7
5		81.6
6		42.9
7	2.03 (d, 13.0)	52.5
	1.11 (d, 13.0)	
8		46.6
9		164.5
10	10.28 (s)	196.2
11	0.94 (d, 6.6)	20.5
12	1.10 (s)	23.4
13	0.90 (s)	25.9
14	1.33 (s)	30.1
15	3.71 (d, 10.2)	70.1
	3.57 (d, 10.2)	
4-OH	4.23 (d, 7.2)	
5-OH	4.33 (s)	
15-OH	5.40 (t, 4.8)	

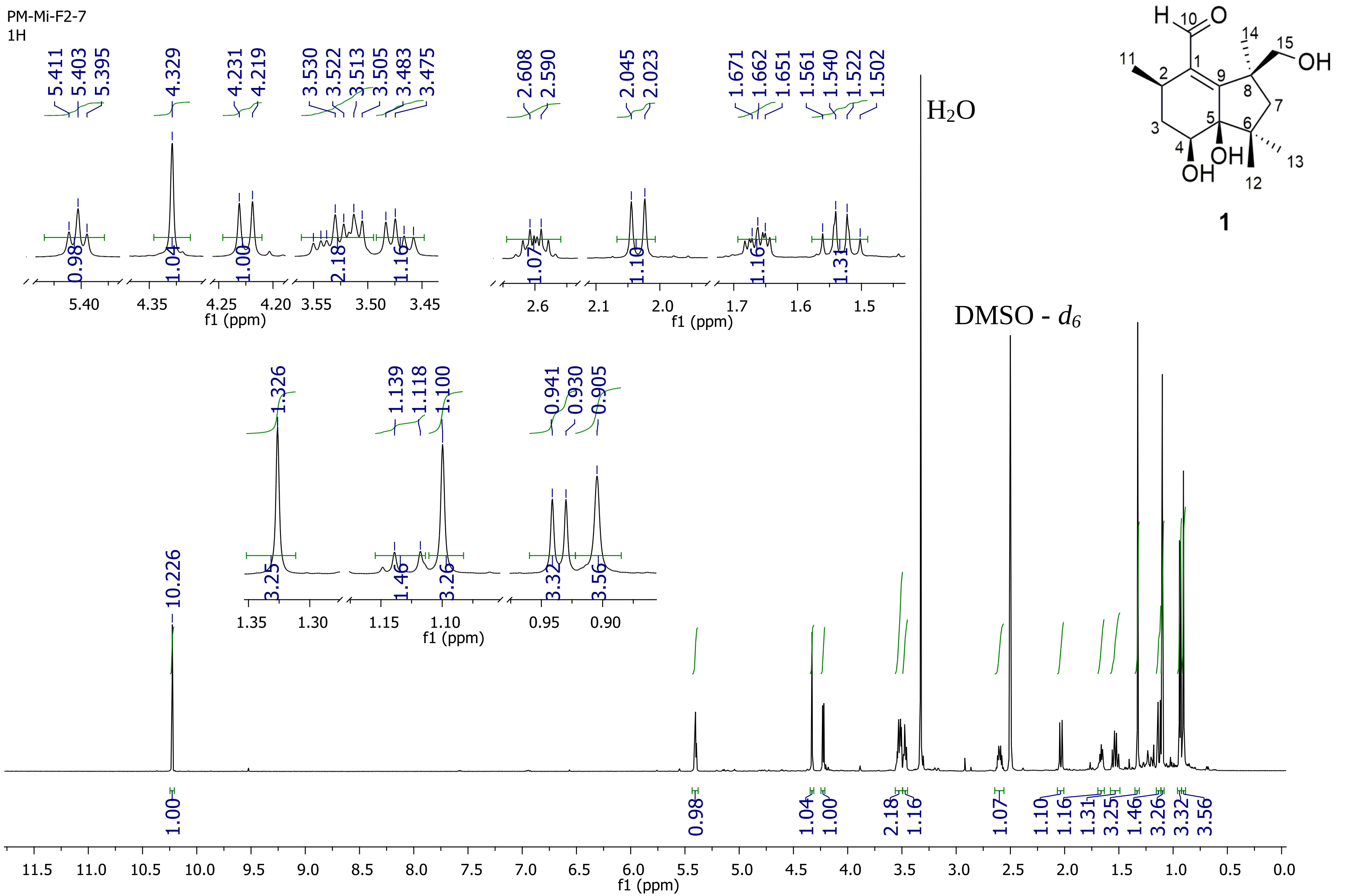

Figure S7 - ^{1}H NMR spectrum of compound **1** (DMSO-d_6; 600 MHz)

PM-Mi-F2-7

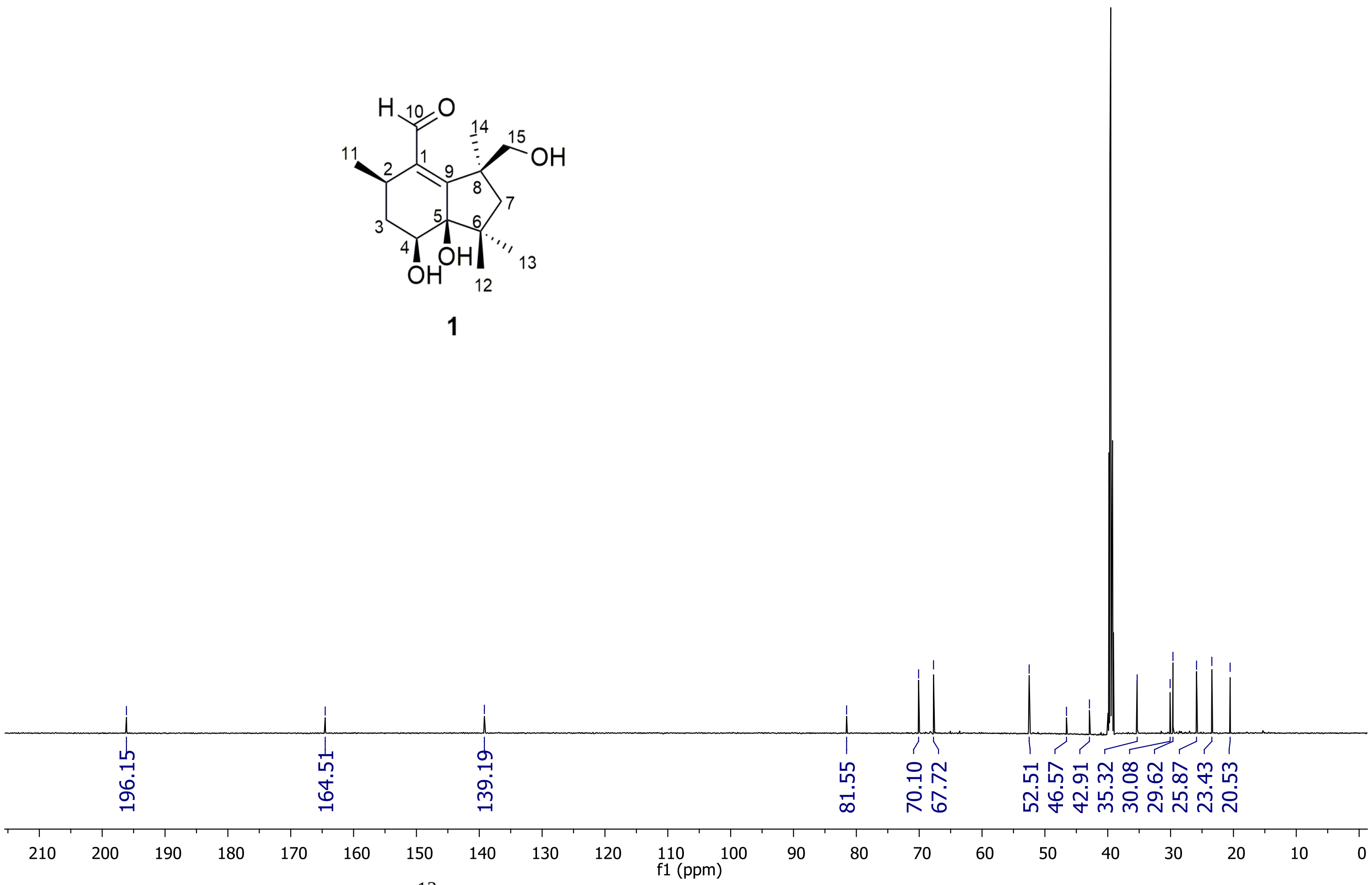

Figura S8 - ^{13}C NMR spectrum of compound **1** (DMSO-d_6; 150 MHz)

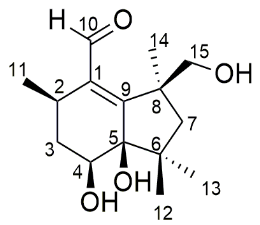

1

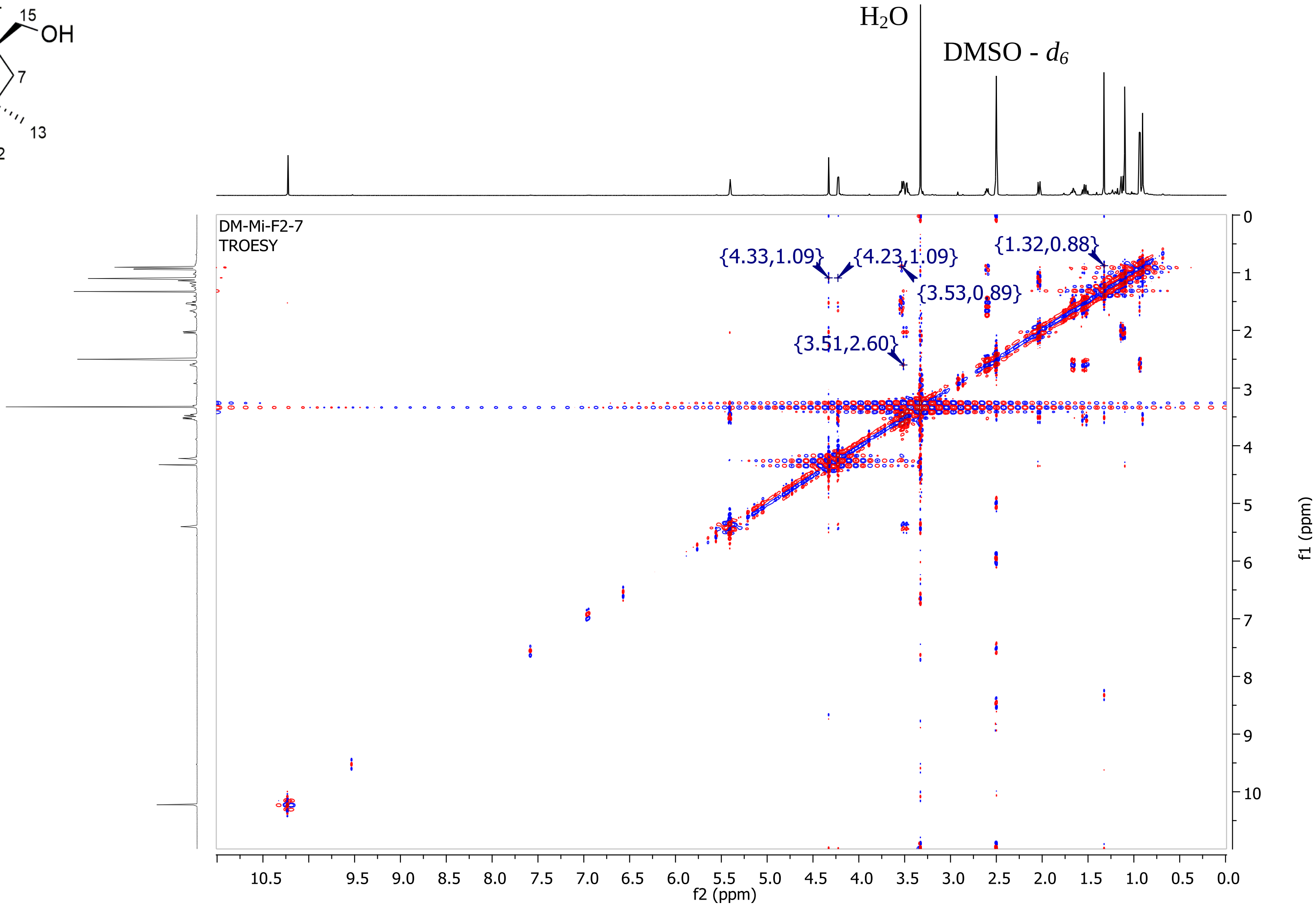

Figure S9 - ROESY spectrum of compound **1** (DMSO-d_6; 600 MHz)

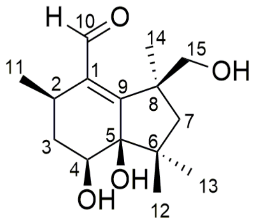

1

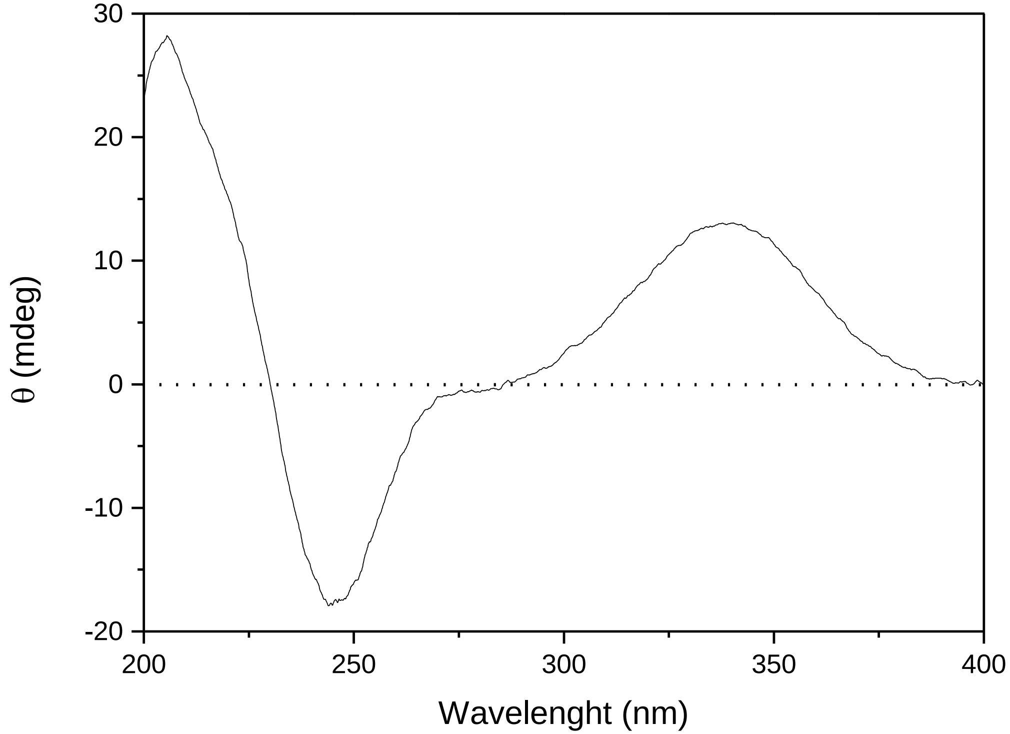

Figure S10 - CD spectrum of compound **1**

Acquisition Parameter

Source Type	ESI	Ion Polarity	Positive	Set Nebulizer	0.3 Bar
Focus	Active	Set Capillary	4500 V	Set Dry Heater	180 °C
Scan Begin	50 m/z	Set End Plate Offset	-500 V	Set Dry Gas	4.0 l/min
Scan End	1500 m/z	Set Charging Voltage	2000 V	Set Divert Valve	Source
		Set Corona	0 nA	Set APCI Heater	0 °C

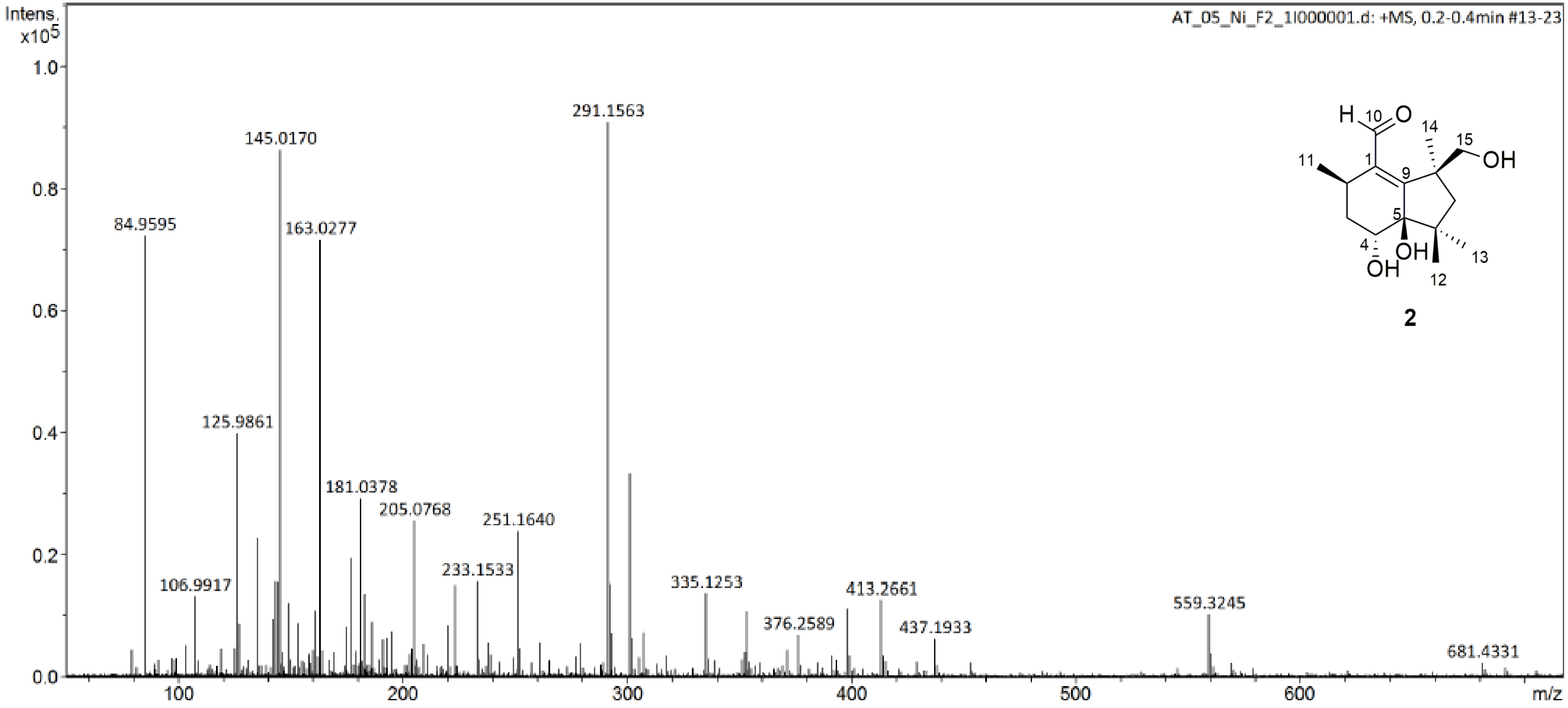

Figure S11 - High resolution ESI-Q-TOF-MS spectrum of compound **2**(Bruker - MaXix Impact)

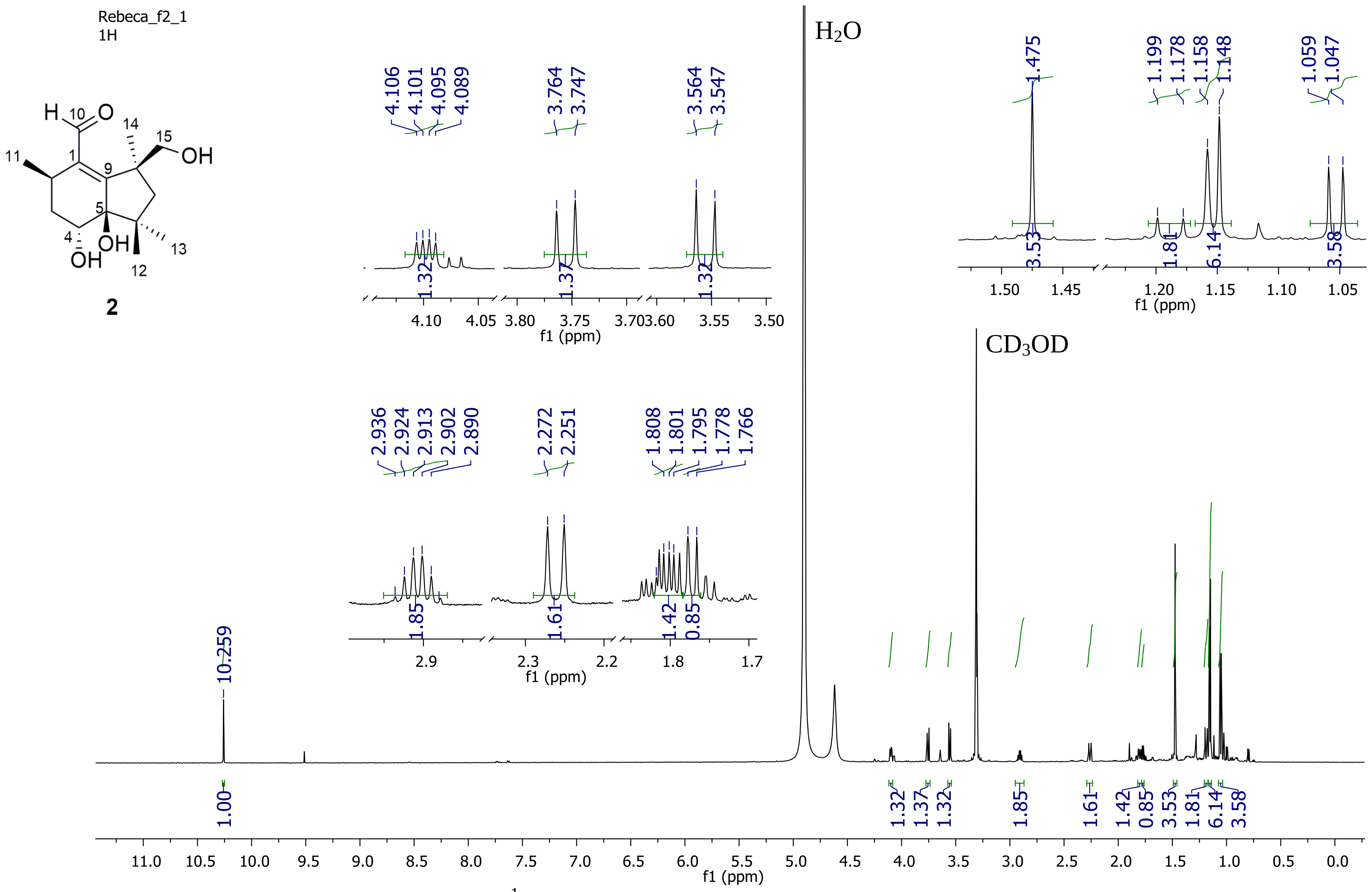

Figure S12 - ^{1}H NMR spectrum of compound **2** (CD_3OD; 600 MHz)

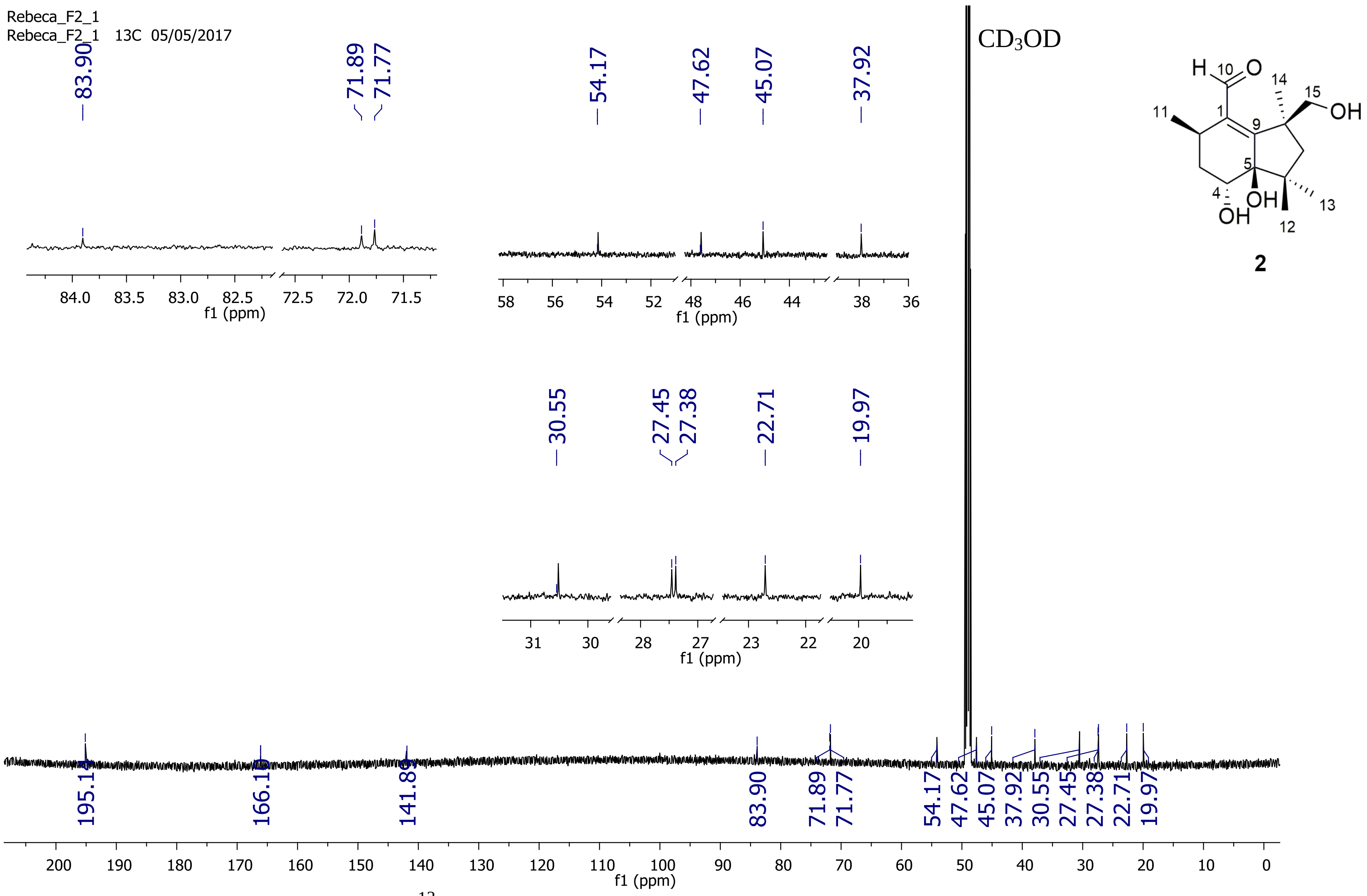

2

Figura S13 - ^{13}C NMR spectrum of compound **2** (CD_3OD; 150 MHz)

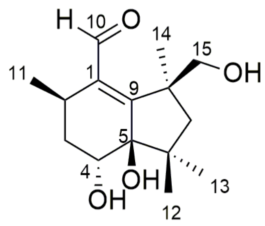

2

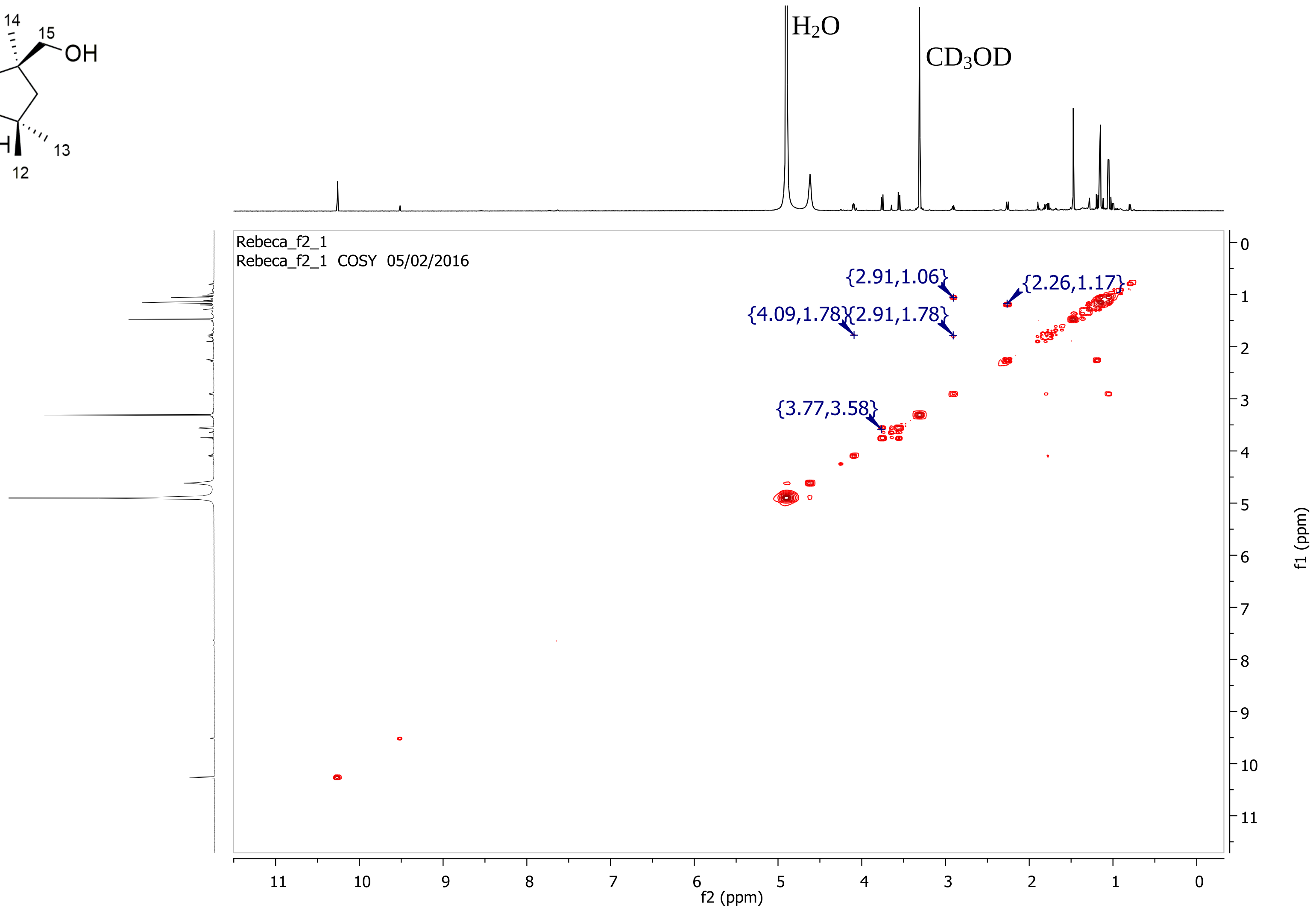

Figure S14 - ^{1}H–^{1}HCOSY spectrum of compound **2** (CD_3OD; 600 MHz)

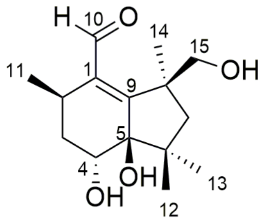

2

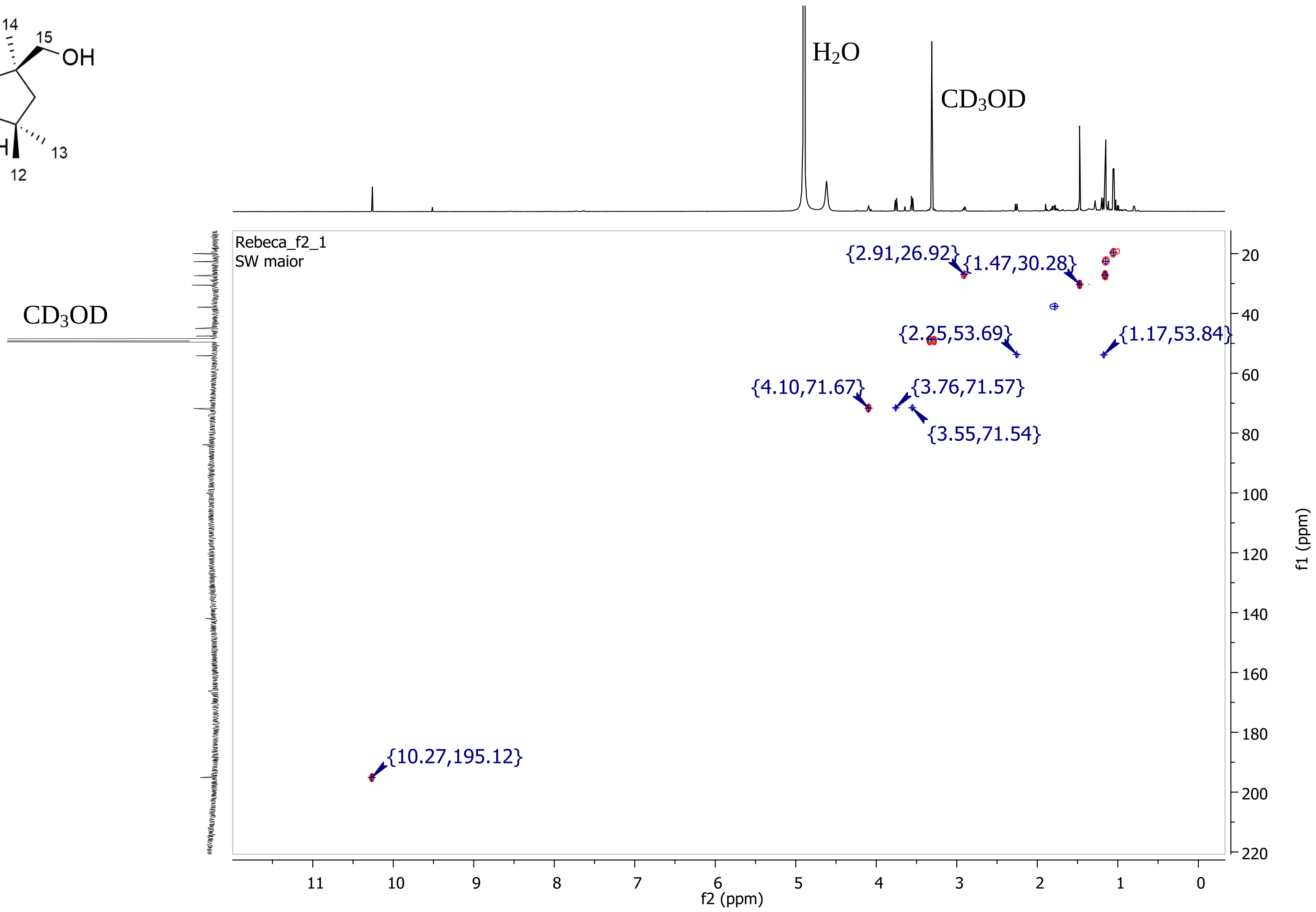

Figure S15 - HSQC spectrum of compound **2** (CD_3OD; 600 and 150 MHz)

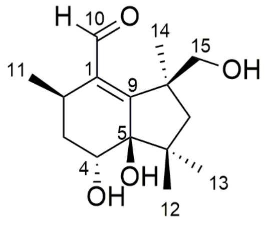

2

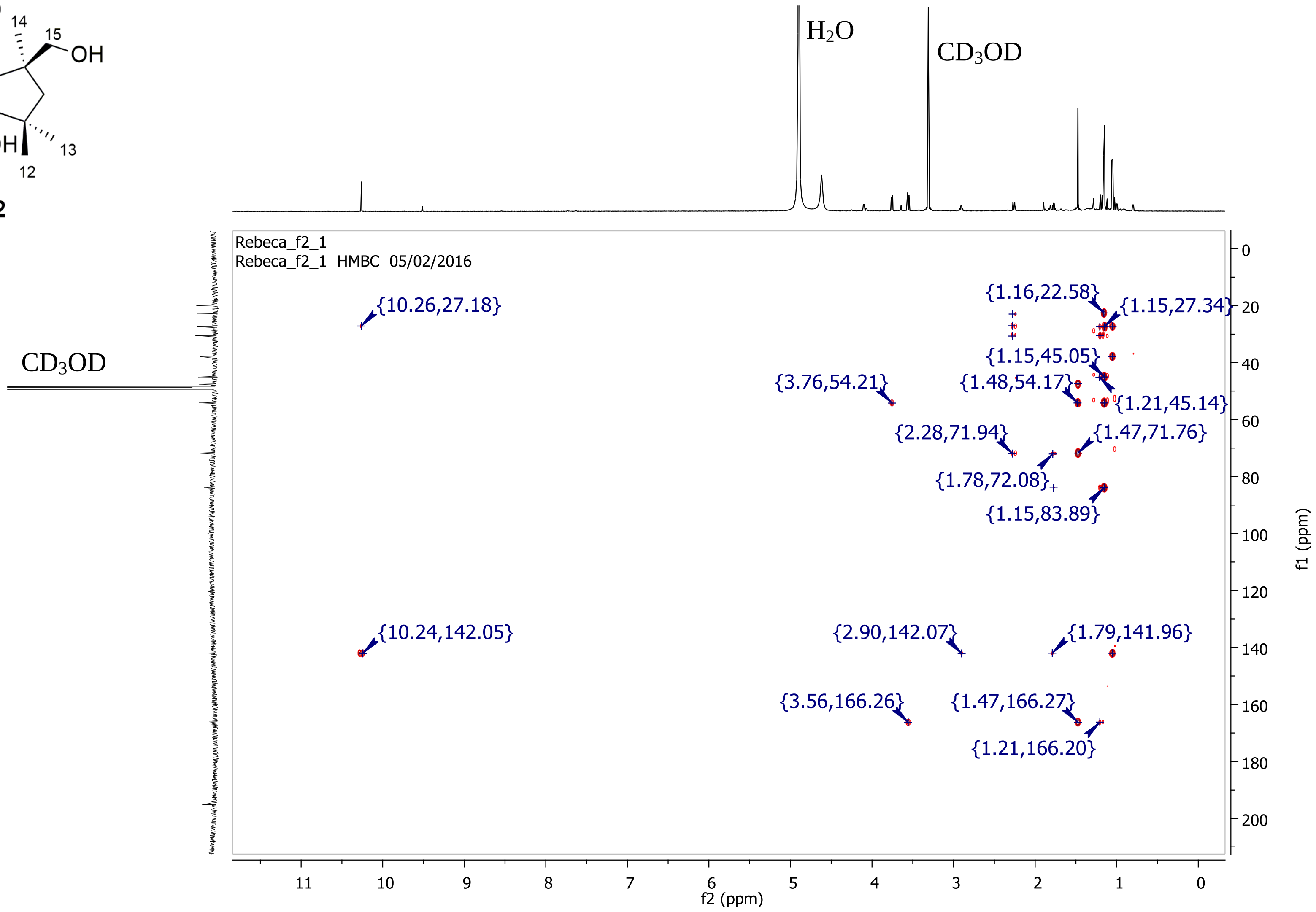

Figure S16 - HMBC spectrum of compound **2** (CD_3OD; 600 and 150 MHz)

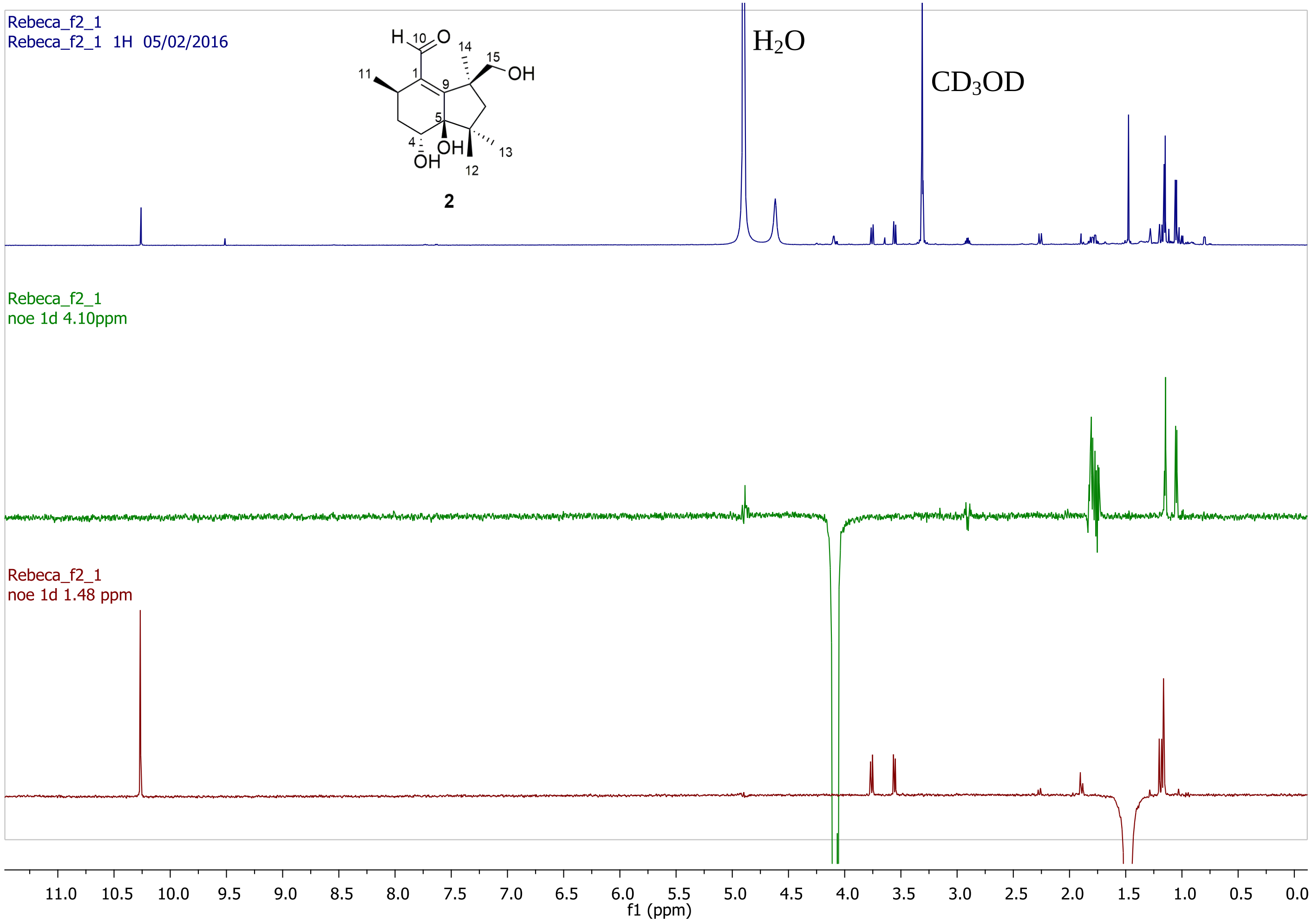

Figure S17 - ^{1}H NMR (blue) and NOESY 1D (green - selected signal: δ_H 4.10; red - selected signal: δ_H1.48) spectra of compound **2** - (CD_3OD; 600 MHz)

Acquisition Parameter

Source Type	ESI	Ion Polarity	Positive	Set Nebulizer	0.3 Bar
Focus	Active	Set Capillary	4500 V	Set Dry Heater	180 °C
Scan Begin	50 m/z	Set End Plate Offset	-500 V	Set Dry Gas	4.0 l/min
Scan End	1500 m/z	Set Charging Voltage	2000 V	Set Divert Valve	Source
		Set Corona	0 nA	Set APCI Heater	0 °C

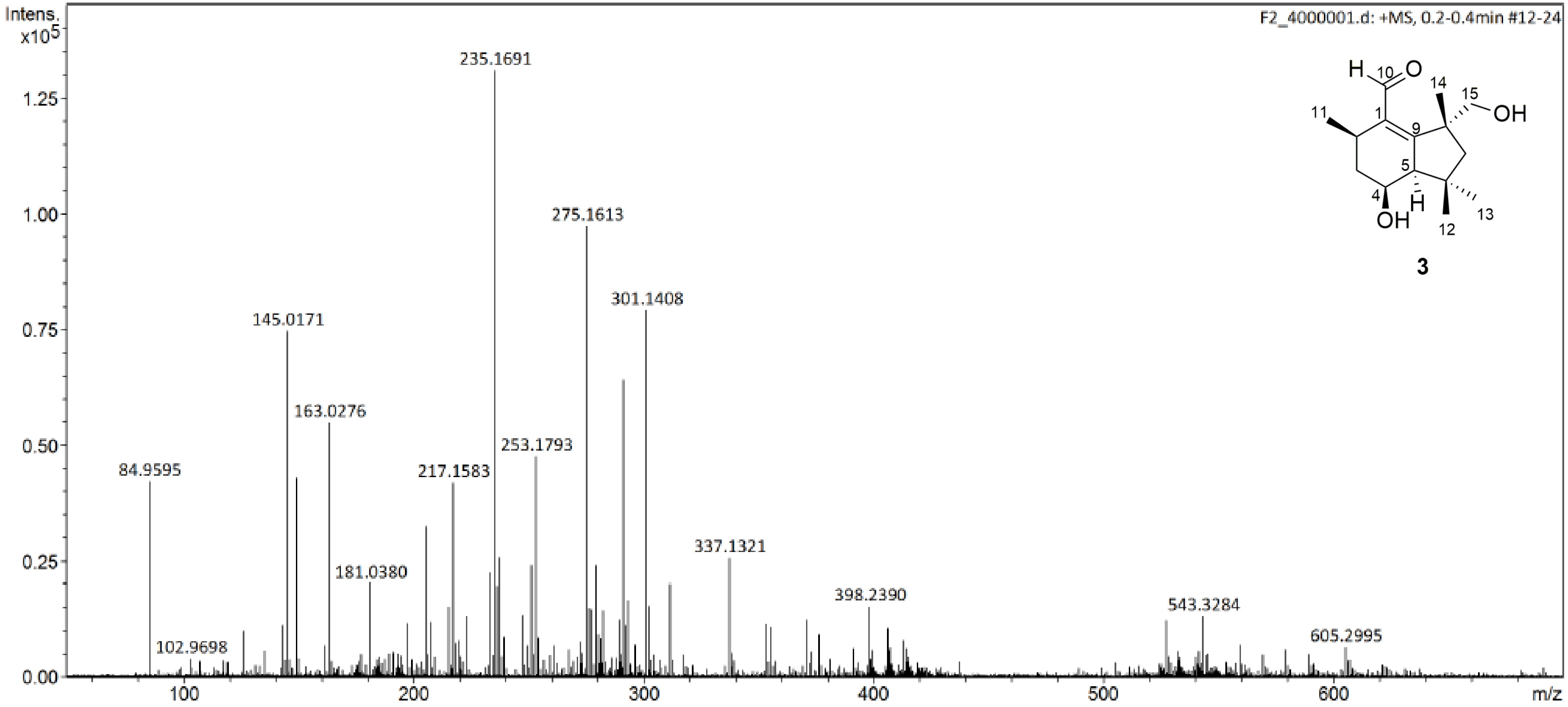

Figure S18 - High resolution ESI-Q-TOF-MS spectrum of compound **3**(Bruker - MaXix Impact)

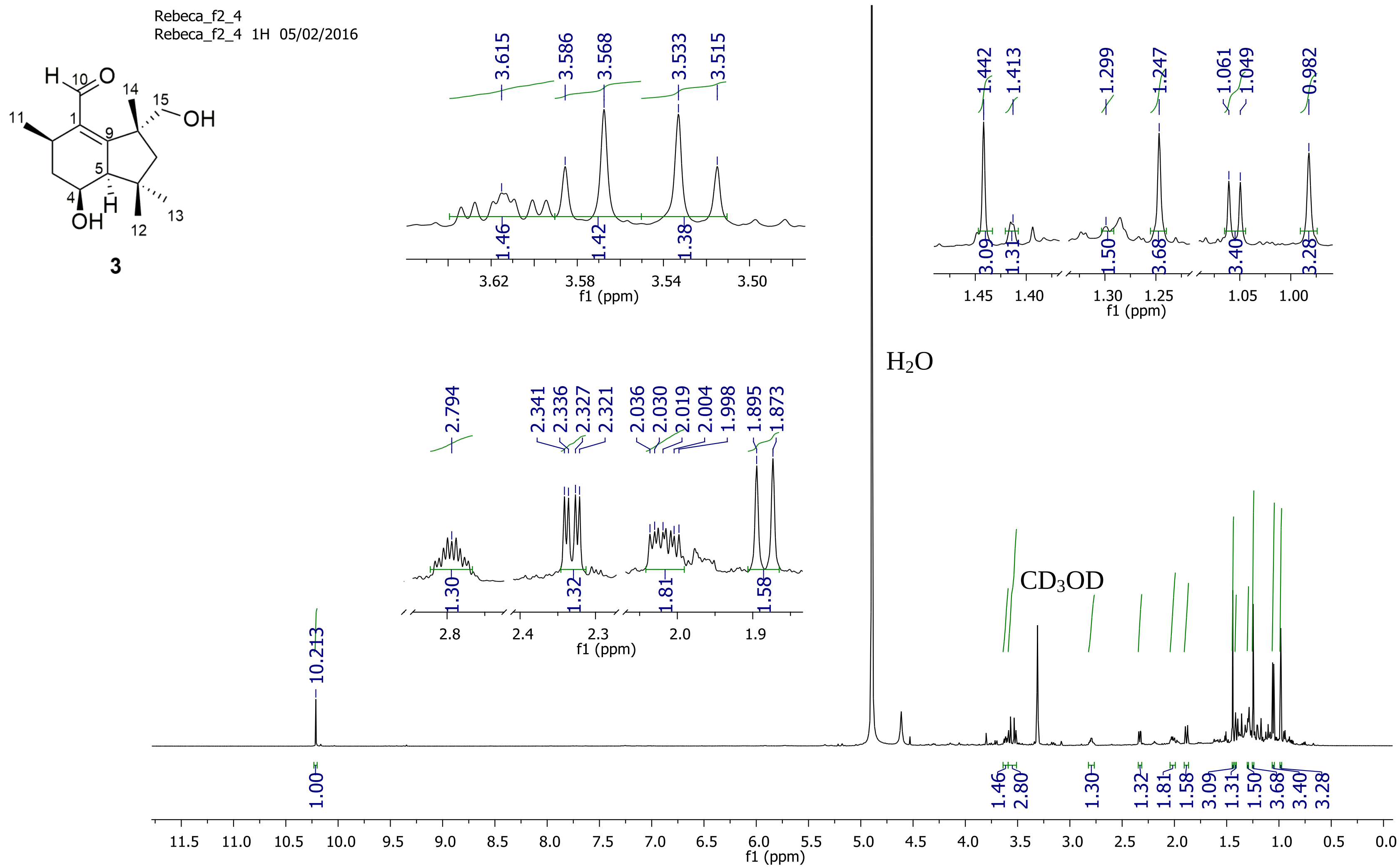

Figure S19 - ^{1}H NMR spectrum of compound **3** (CD_3OD; 600 MHz)

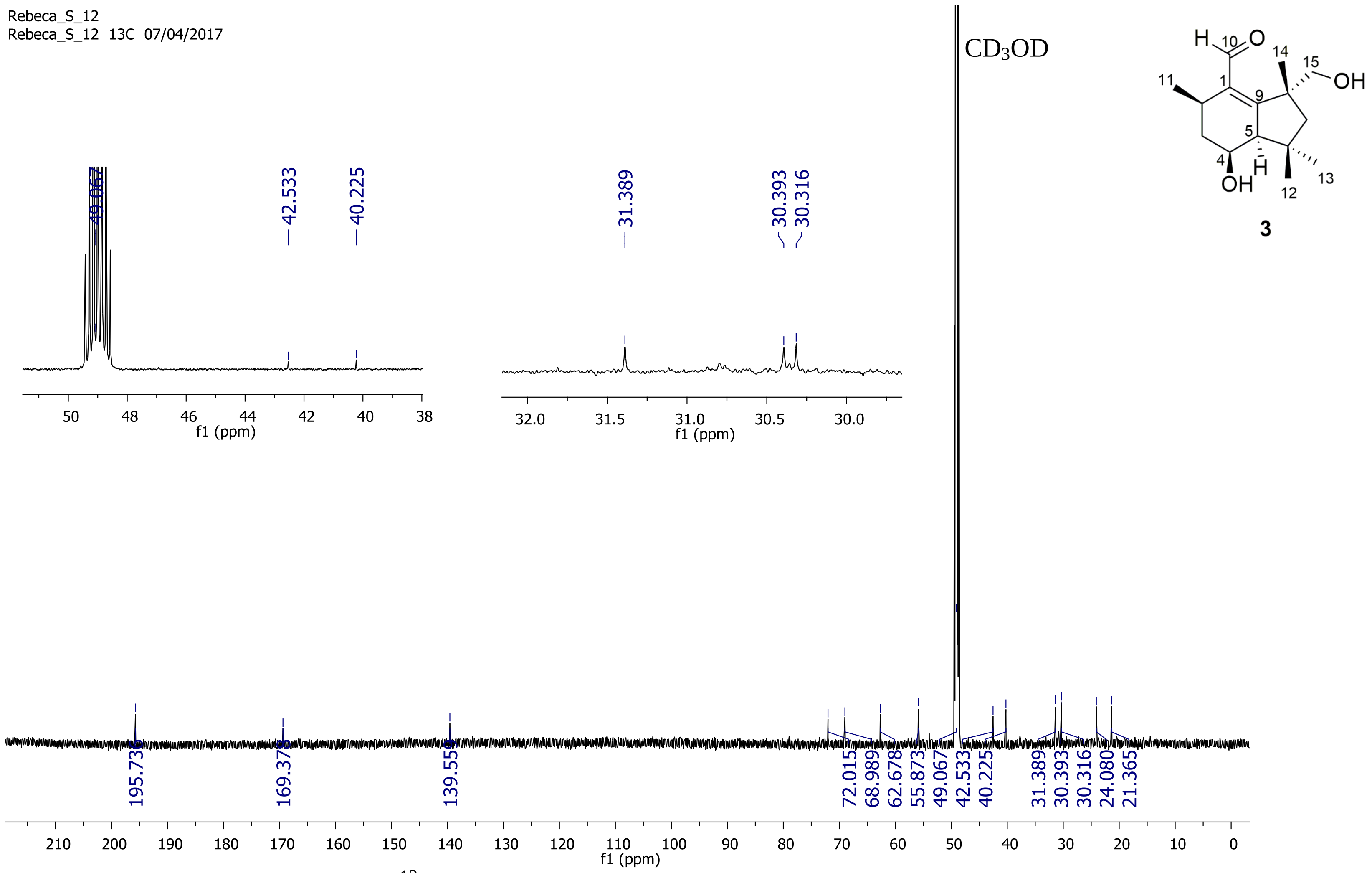

Figure S20 - ^{13}C NMR spectrum of compound **3** (CD_3OD; 150 MHz)

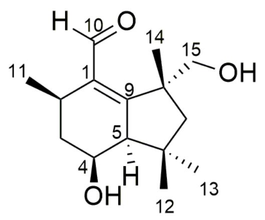

3

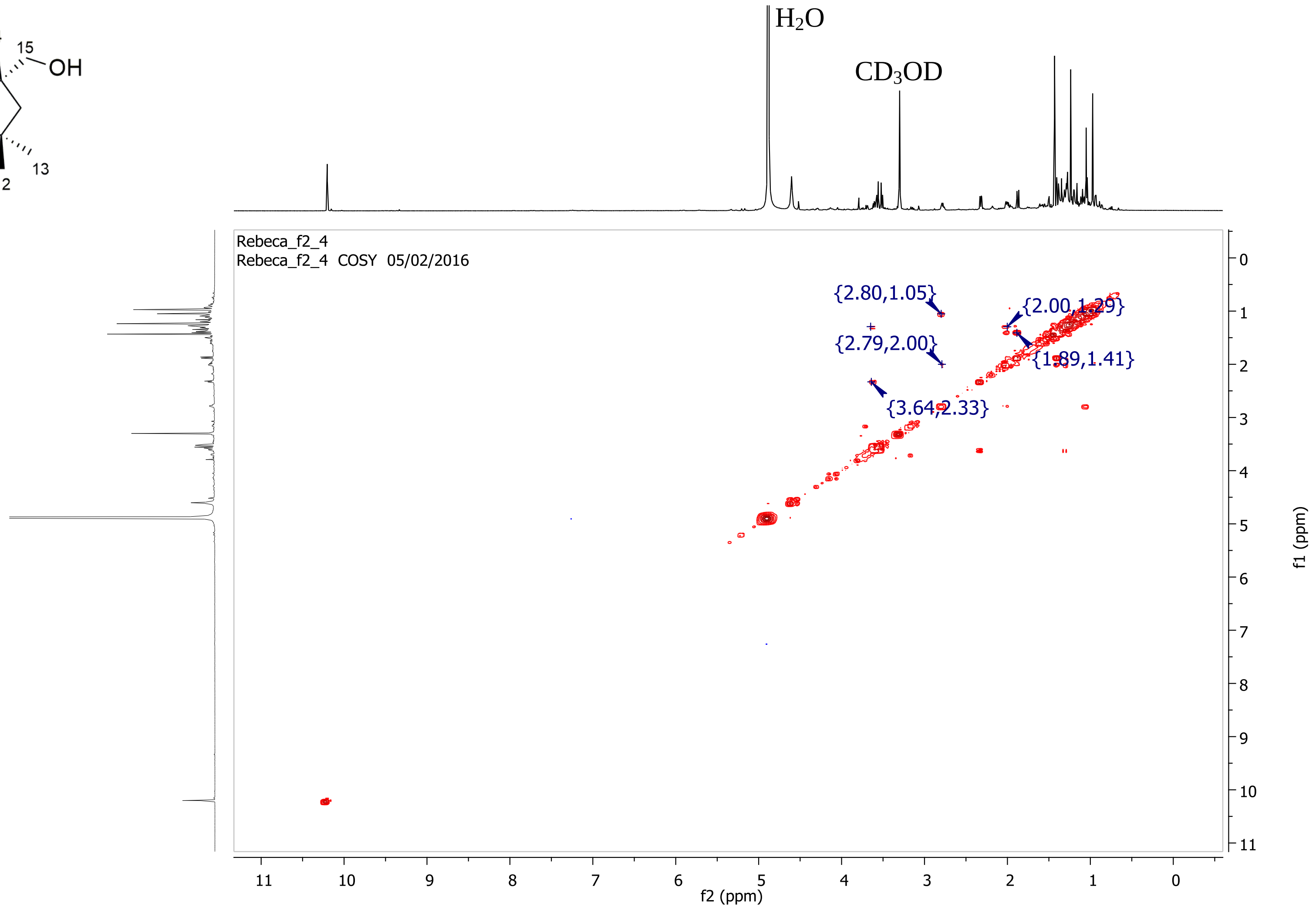

Figure S21 - ^{1}H–^{1}HCOSY spectrum of compound **3** (CD_3OD; 600 MHz)

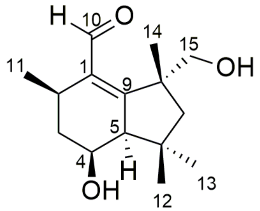

3

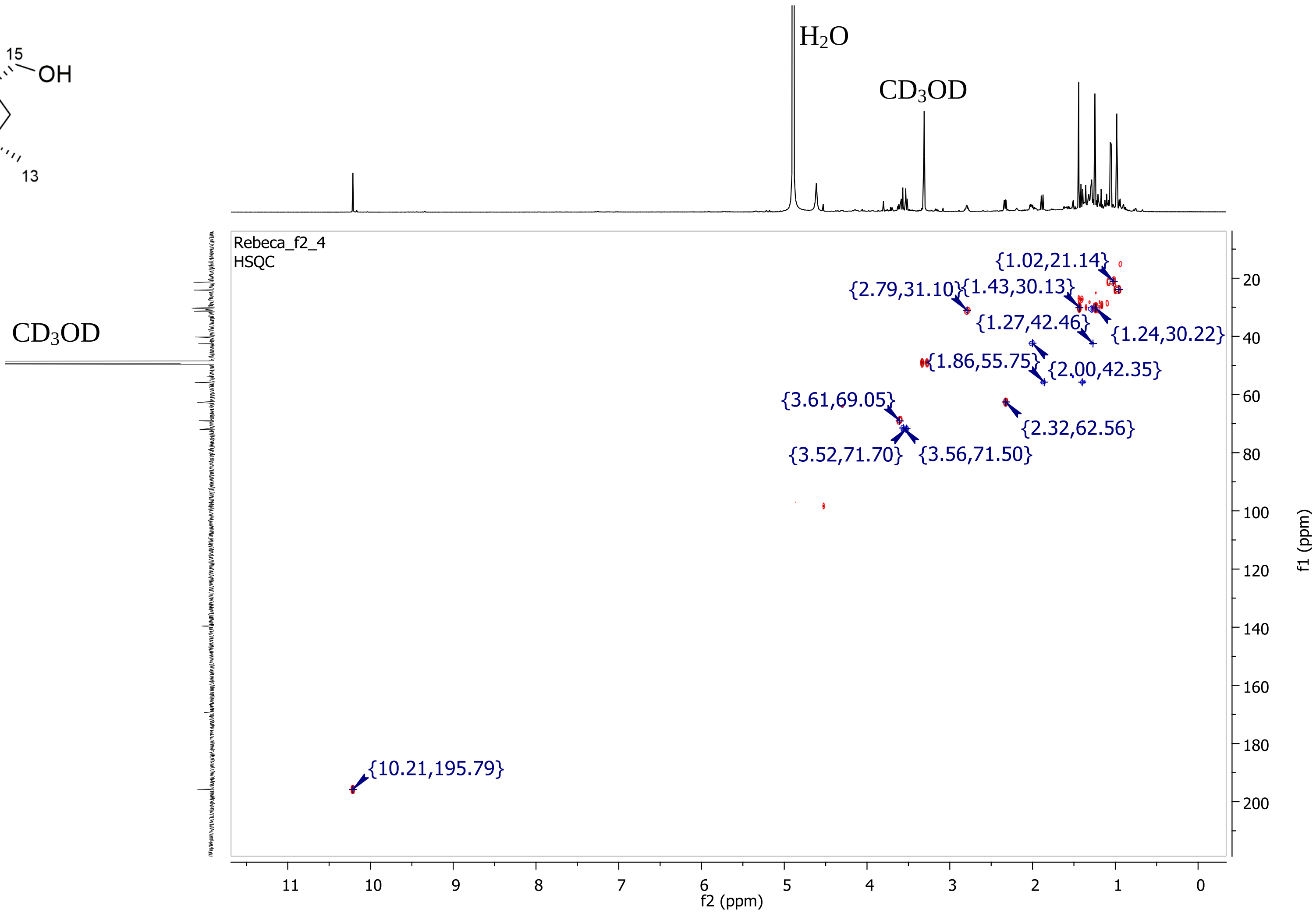

Figure S22 - HSQC spectrum of compound **3** (CD_3OD; 600 and 150 MHz)

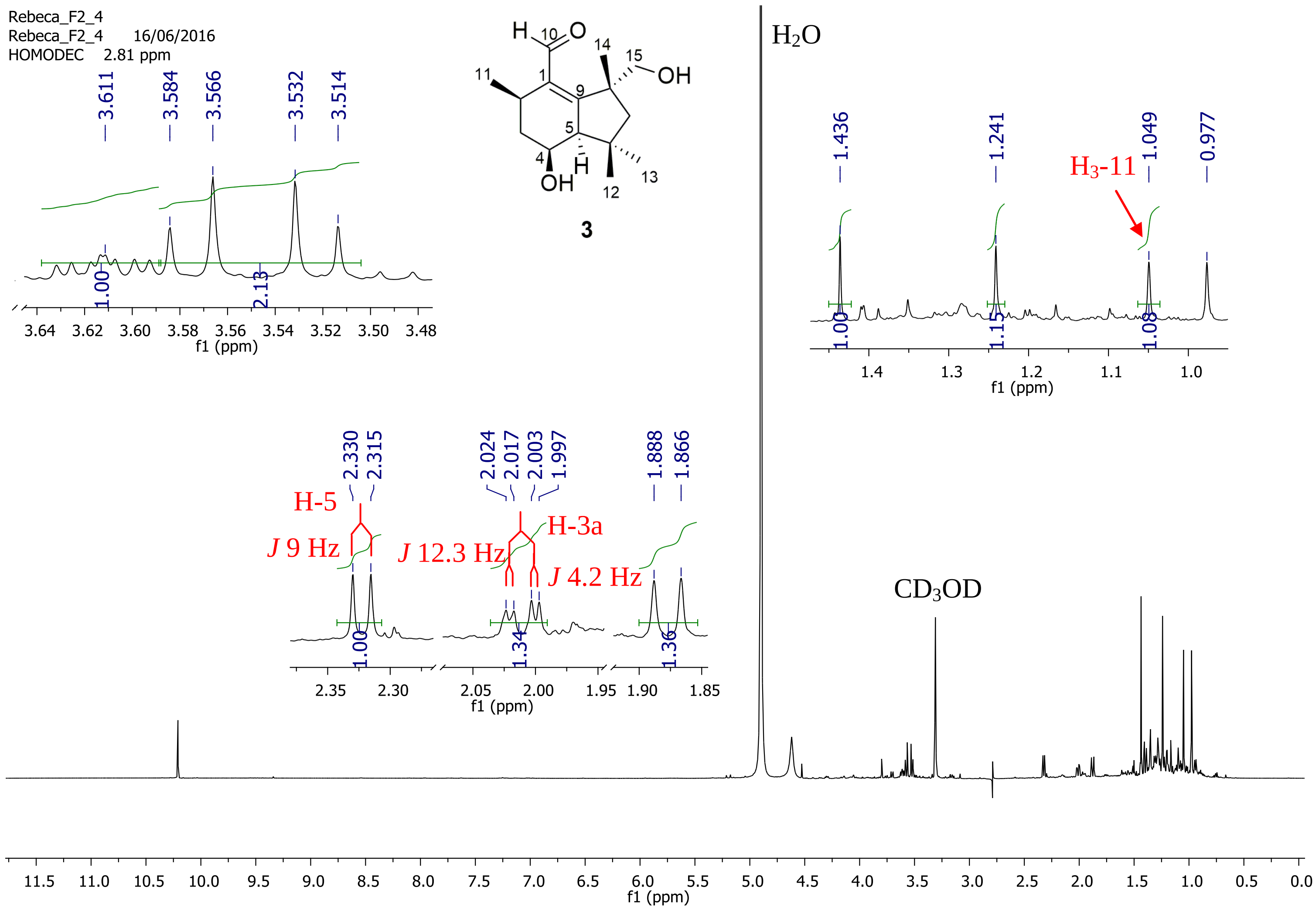

Figure S23 - HOMODEC experiment upon irradiation of signal δ_H 2.79 (H-2) of compound **3** - (CD_3OD; 600 MHz)

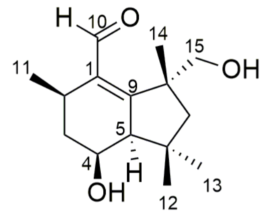

3

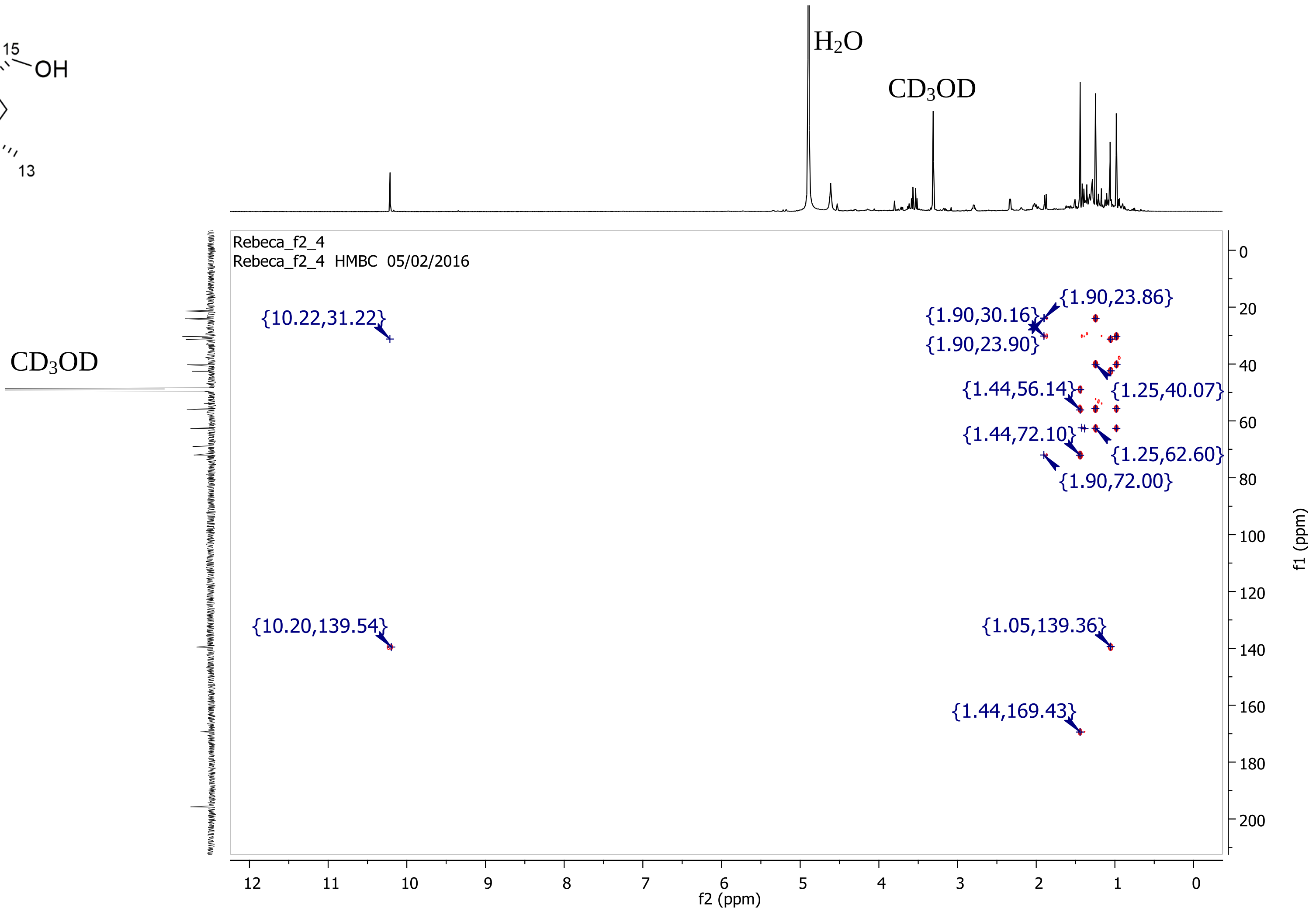

Figure S24 - HMBC spectrum of compound **3** (CD_3OD; 600 and 150 MHz)

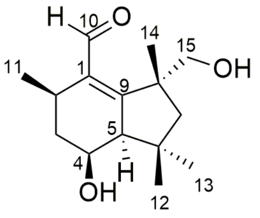

3

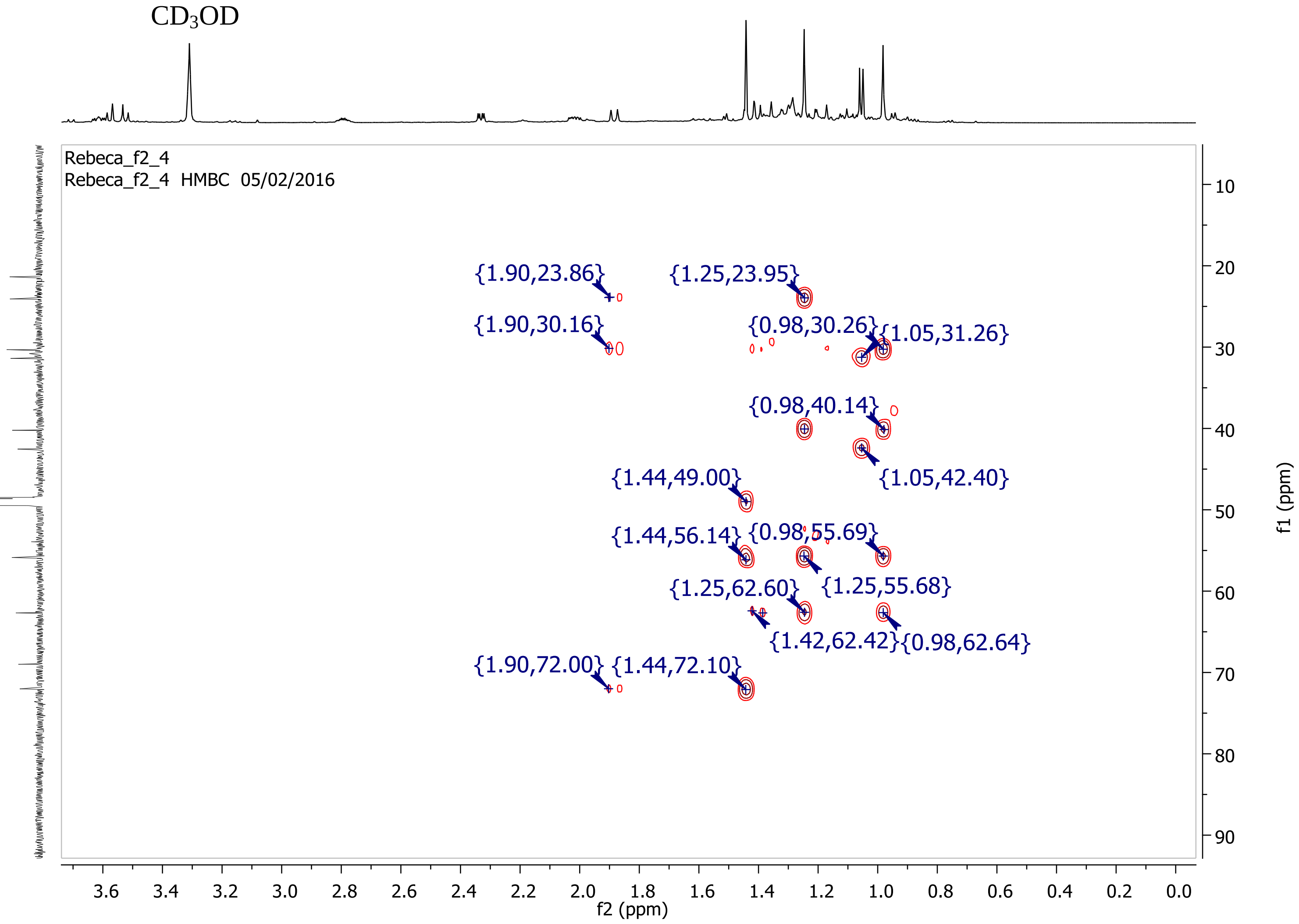

Figure S25 - HMBC spectrum expansion of compound **3** (CD_3OD; 600 and 150 MHz)

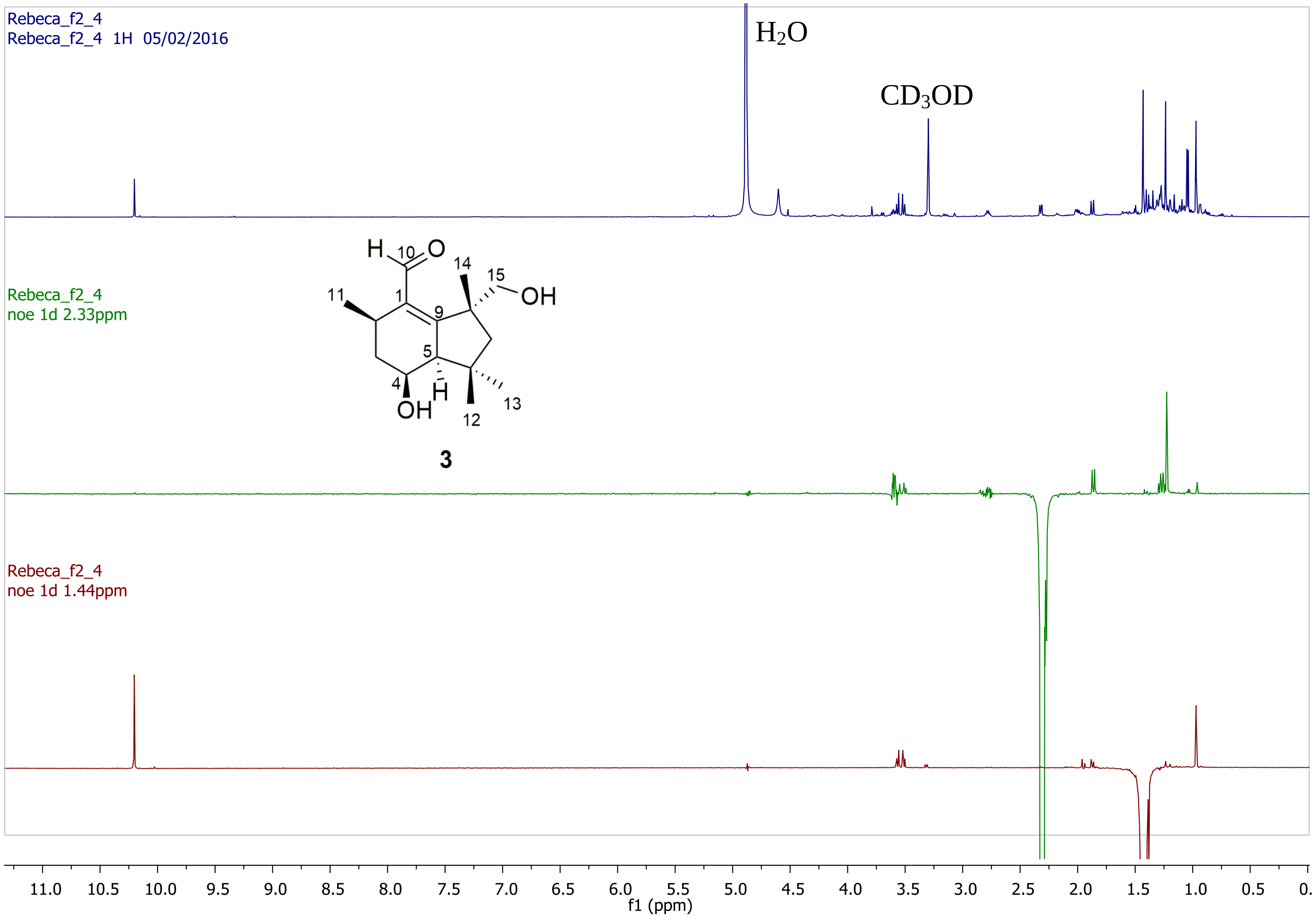

Figure S26 - ^{1}H NMR (blue) and NOESY 1D (green - selected signal: δ_H2.33; red - selected signal: δ_H 1.44) spectra of compound **3** (CD_3OD; 600 MHz)

Elemental Composition Report

Page 1

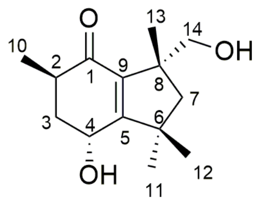

4

Single Mass Analysis

Tolerance = 5.0 PPM / DBE: min = -3.0, max = 100.0
Element prediction: Off
Number of isotope peaks used for i-FIT = 3

Monoisotopic Mass, Even Electron Ions
623 formula(e) evaluated with 2 results within limits (all results (up to 1000) for each mass)
Elements Used:
C: 0-60 H: 0-80 N: 0-10 O: 0-10 Na: 0-1
PMi-6
EH9297-2 236 (4.332) AM (Cen,4, 50.00, Ar,5000.0,275.21,1.00); Sm (SG, 1x1.00); Sb (1,40.00); Cm (235:239)

21-Sep-2015
TOF MS ES+
5.04e+003

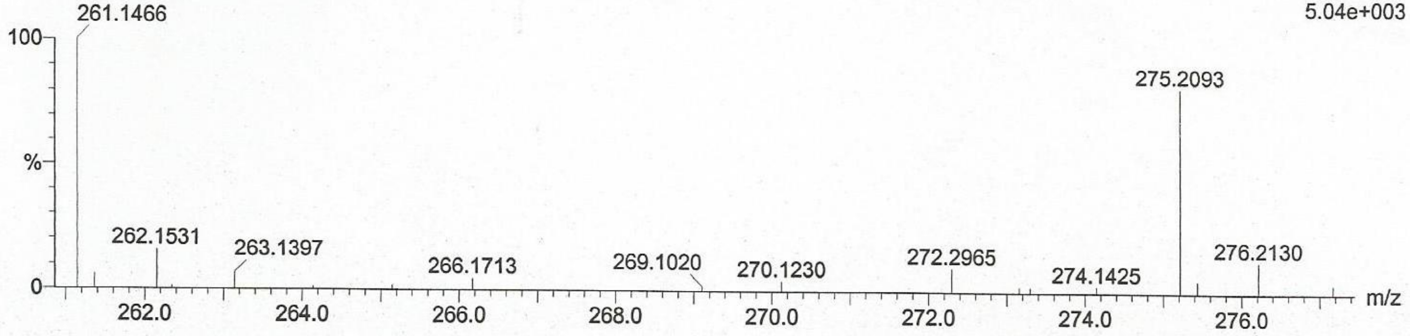

Minimum:				-3.0		
Maximum:		5.0	5.0	100.0		
Mass	Calc. Mass	mDa	PPM	DBE	i-FIT	Formula
261.1466	261.1467	-0.1	-0.4	3.5	85.5	C14 H22 O3 Na
	261.1464	0.2	0.8	7.5	100.9	C12 H17 N6 O

Figure S27 - High resolution ESI-TOF-MS spectrum of compound **4** (Waters/Micromass LCT)

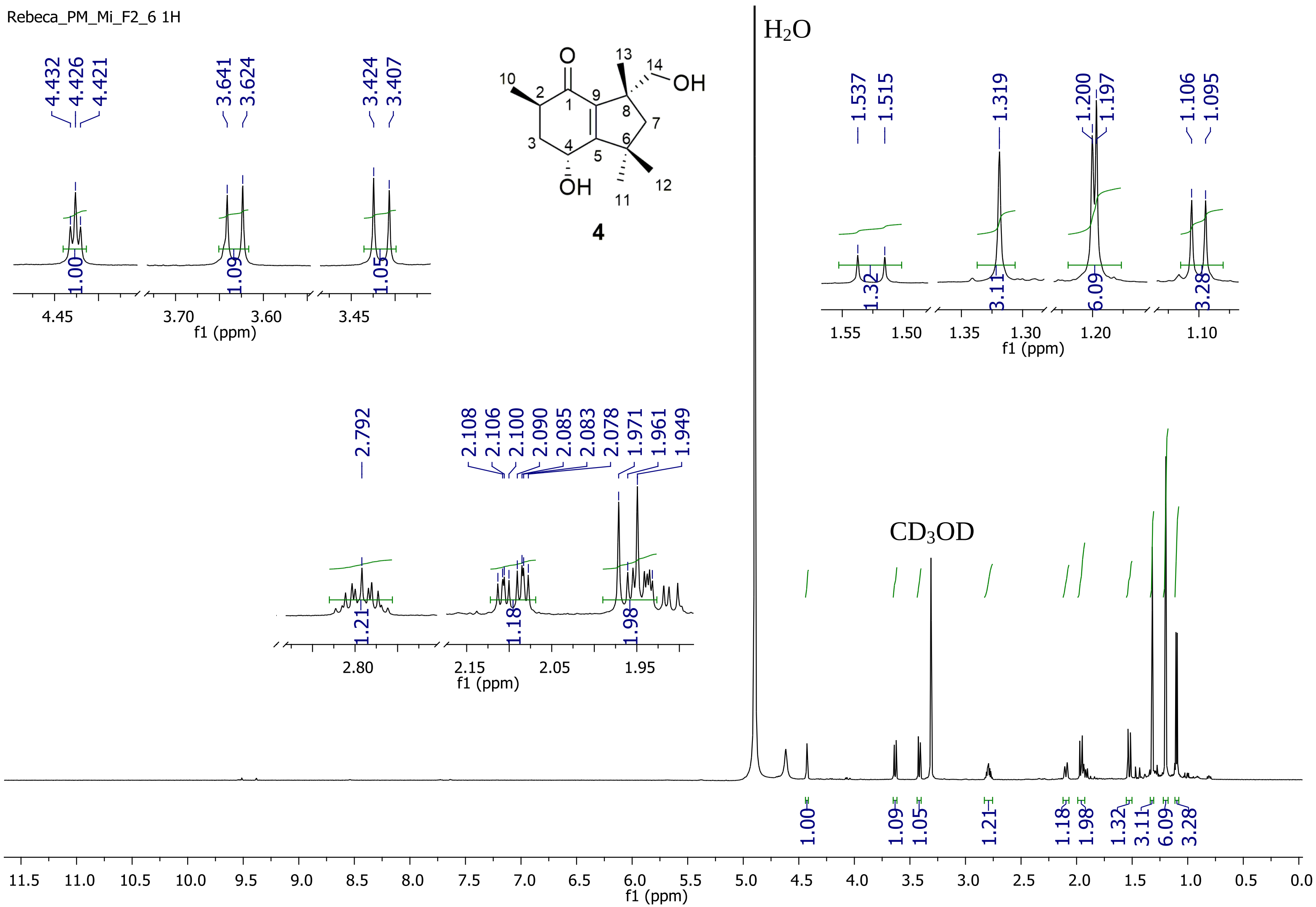

Figure S28 - ^{1}H NMR spectrum of compound **4** (CD_3OD; 600 MHz)

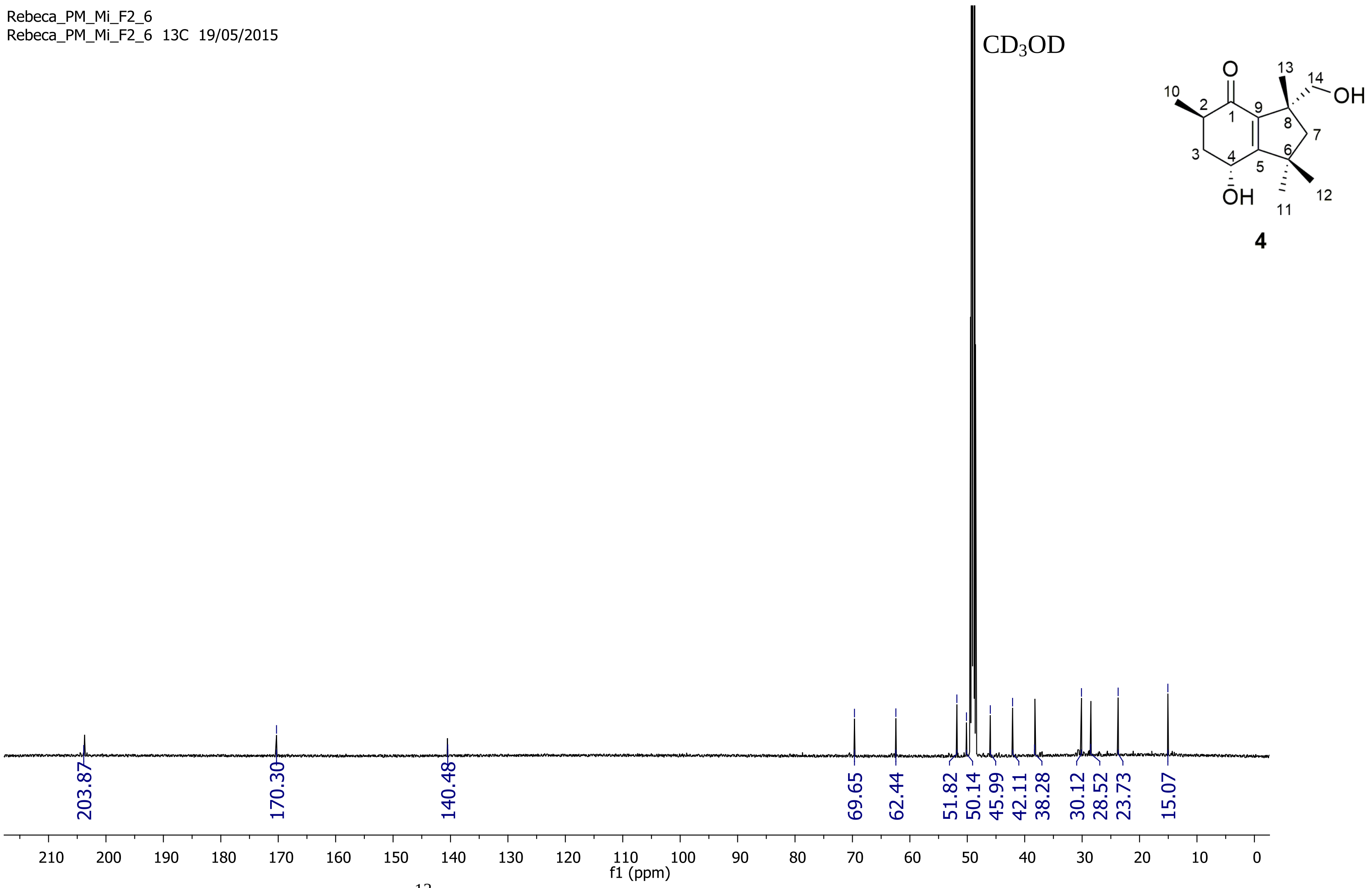

Figura S29 - ^{13}C NMR spectrum of compound **4** (CD_3OD; 150 MHz)

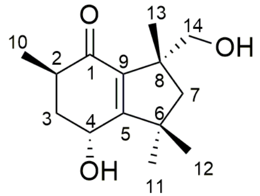

4

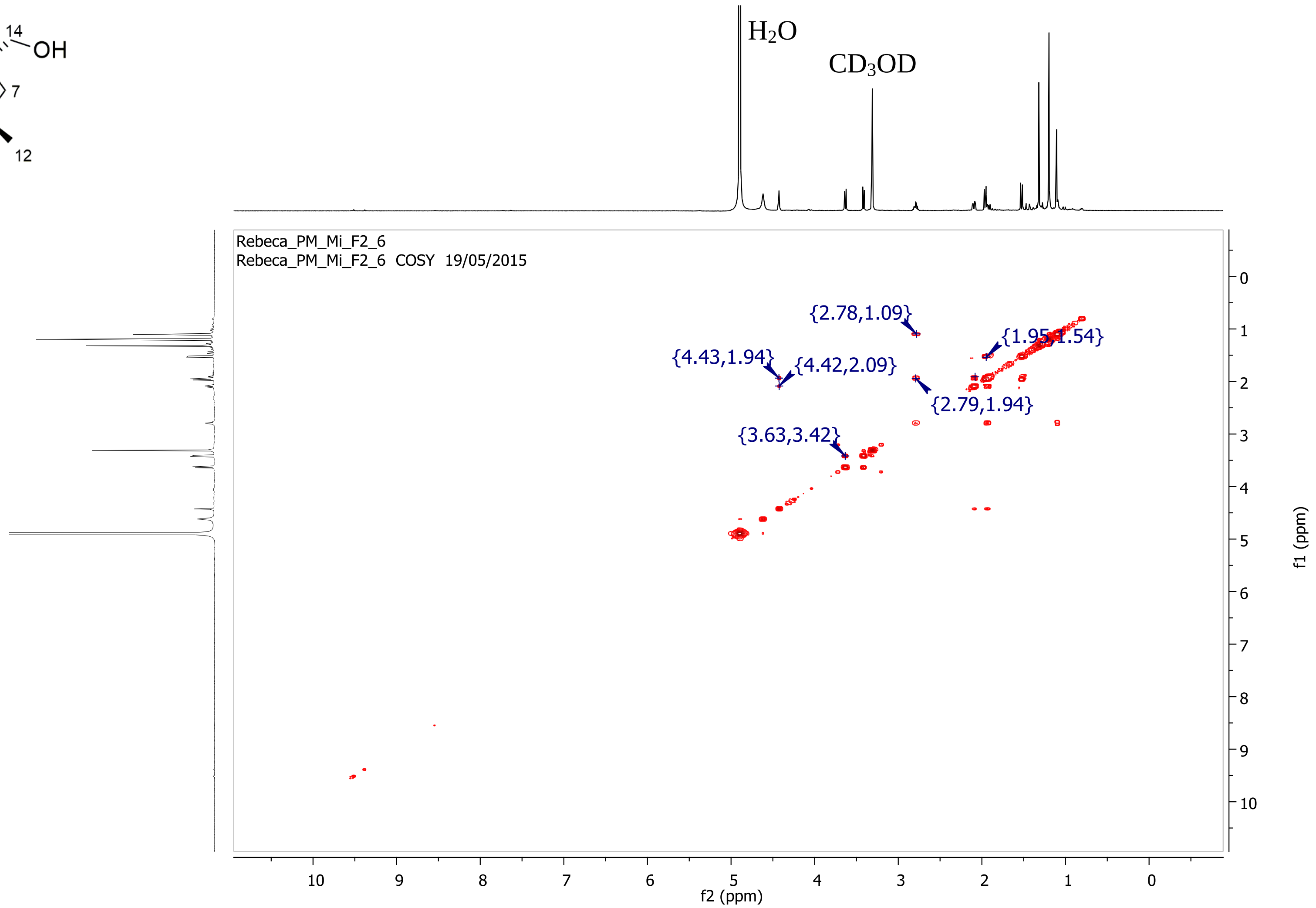

Figure S30 - ^{1}H–^{1}HCOSY spectrum of compound **4** (CD_3OD; 600 MHz)

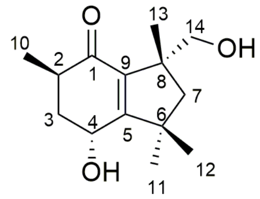

4

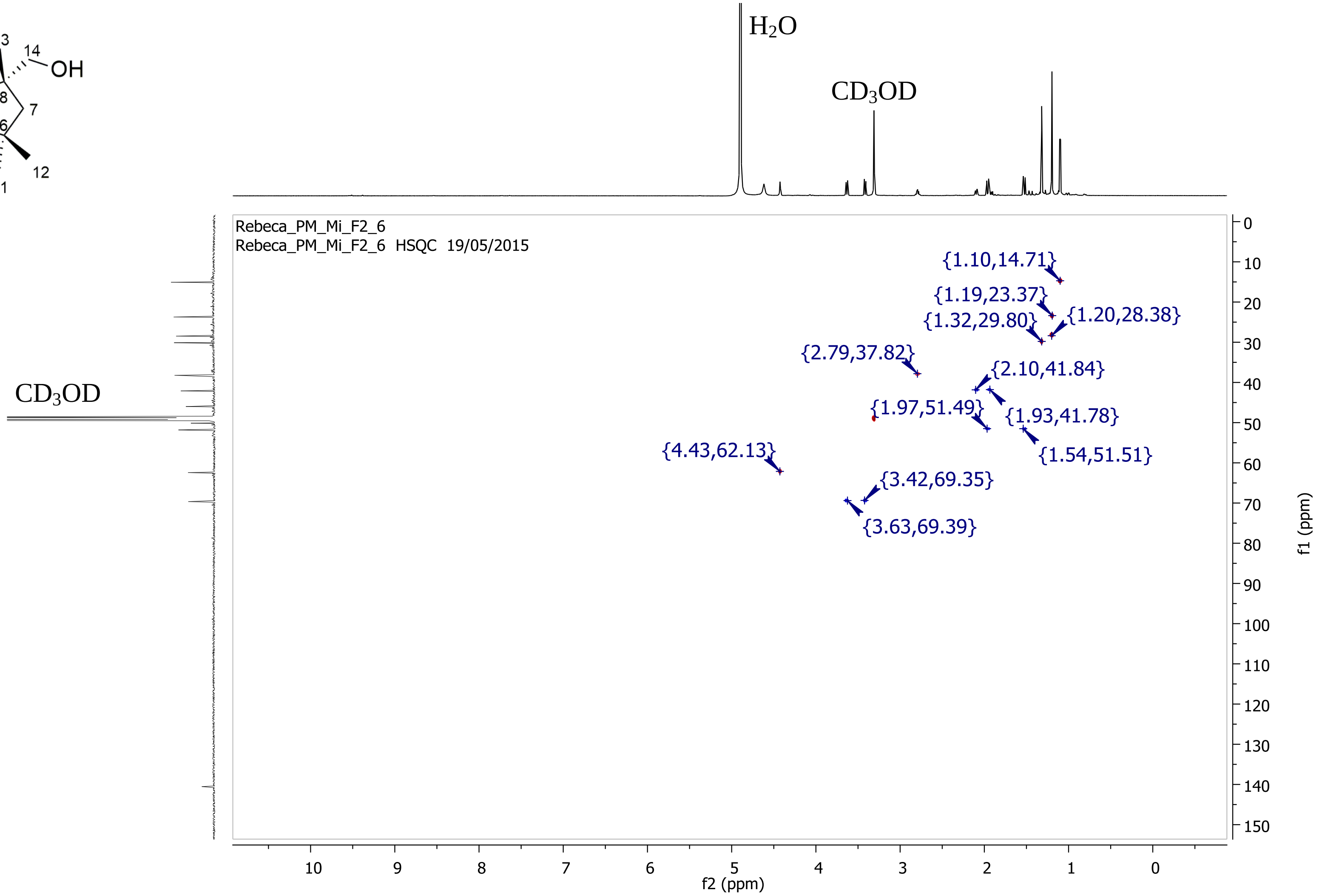

Figure S31 - HSQC spectrum of compound **4** (CD_3OD; 600 and 150 MHz)

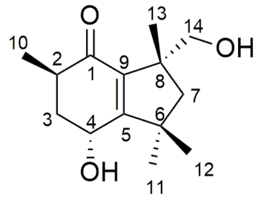

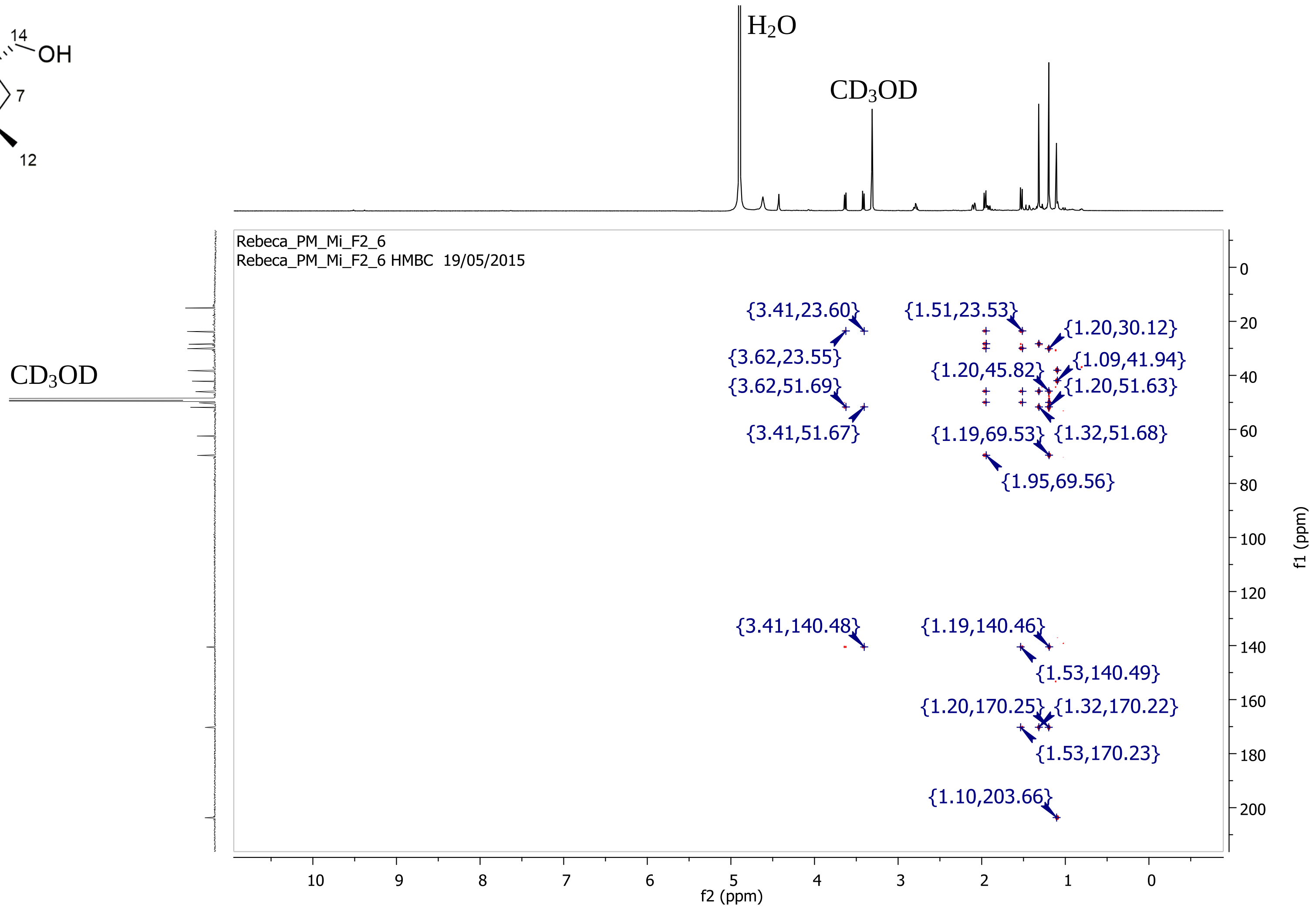

Figure S32 - HMBC spectrum of compound **4** (CD_3OD; 600 and 150 MHz)

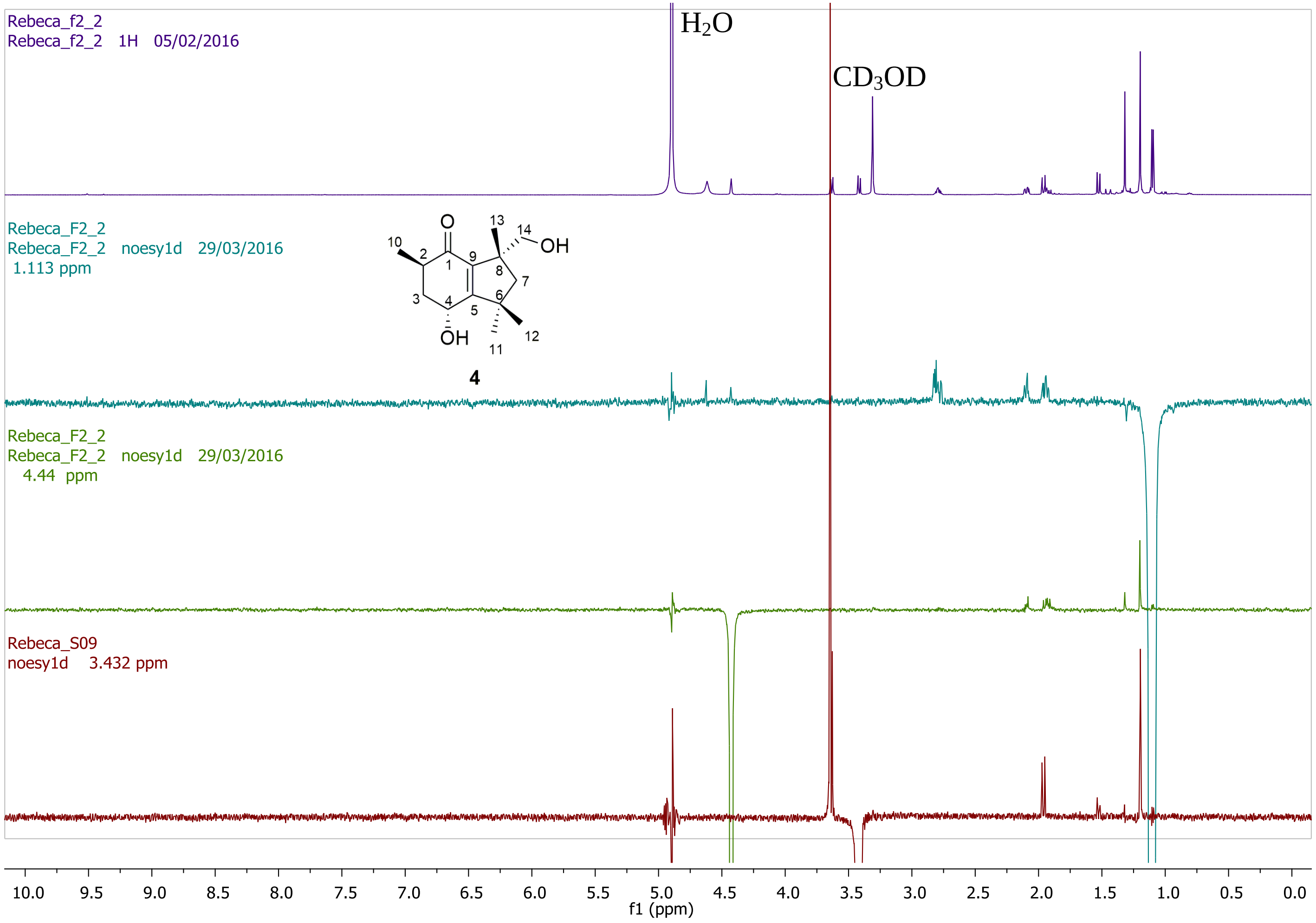

Figure S33- ^{1}H NMR (purple) and NOESY 1D (light blue - selected signal: δ_H 1.10; green - selected signal: δ_H 4.43; red - selected signal: δ_H 3.42) spectra of compound **4** - (CD_3OD; 600 MHz).

Acquisition Parameter

Source Type	ESI	Ion Polarity	Positive	Set Nebulizer	0.3 Bar
Focus	Active	Set Capillary	4500 V	Set Dry Heater	180 °C
Scan Begin	50 m/z	Set End Plate Offset	-500 V	Set Dry Gas	4.0 l/min
Scan End	1500 m/z	Set Charging Voltage	2000 V	Set Divert Valve	Source
		Set Corona	0 nA	Set APCI Heater	0 °C

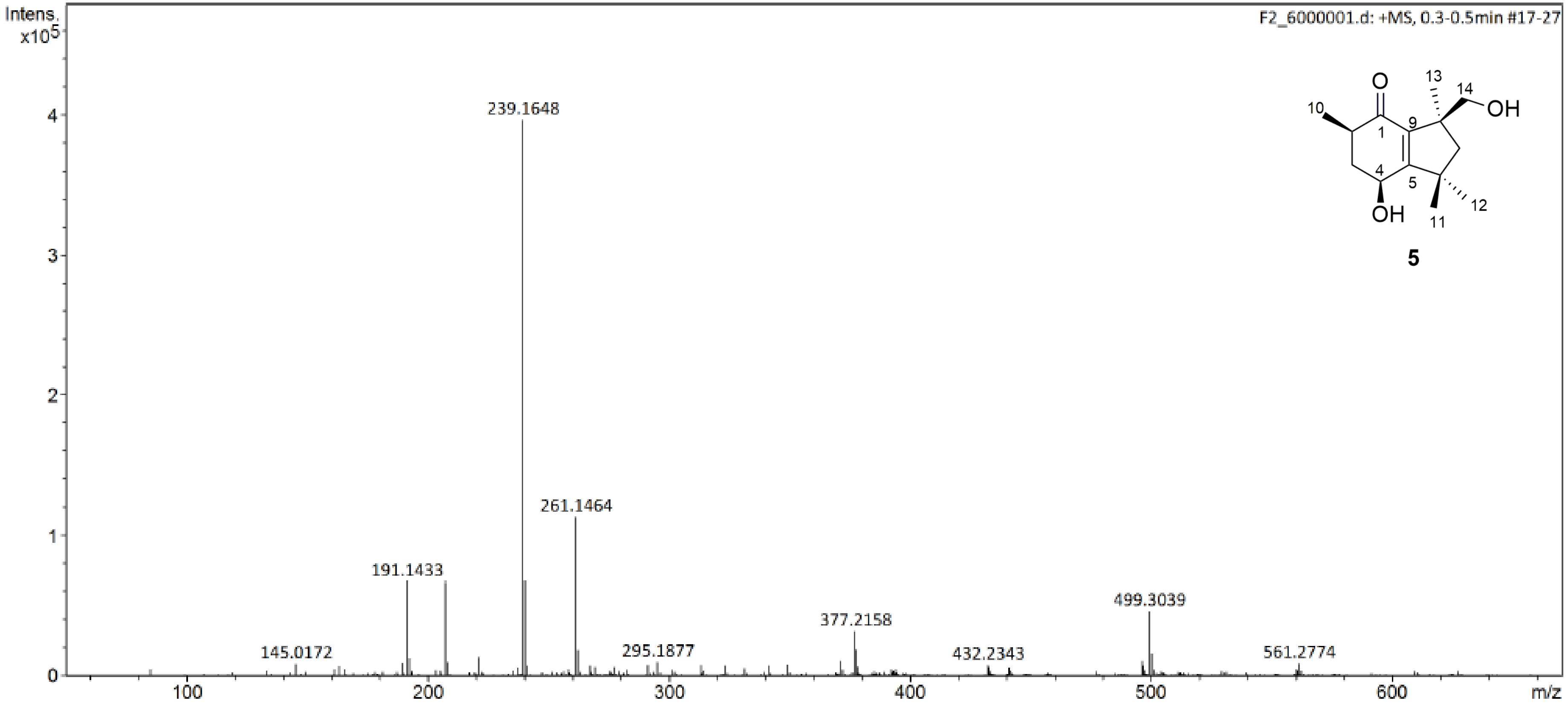

Figure S34 - High resolution ESI-Q-TOF-MS spectrum of compound **5**(Bruker - MaXix Impact)

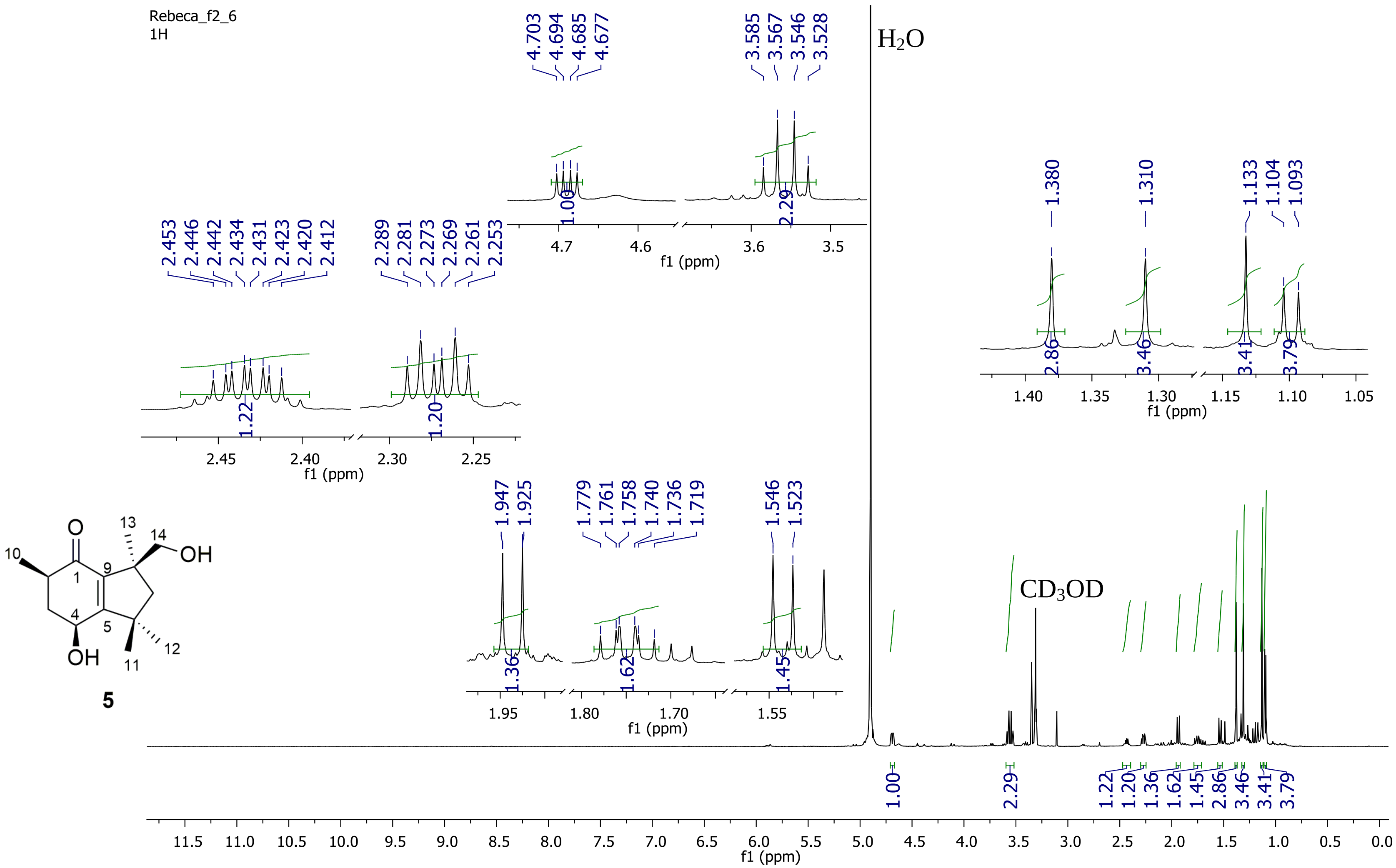

Figure S35 - ^{1}H NMR spectrum of compound **5** (CD_3OD; 600 MHz)

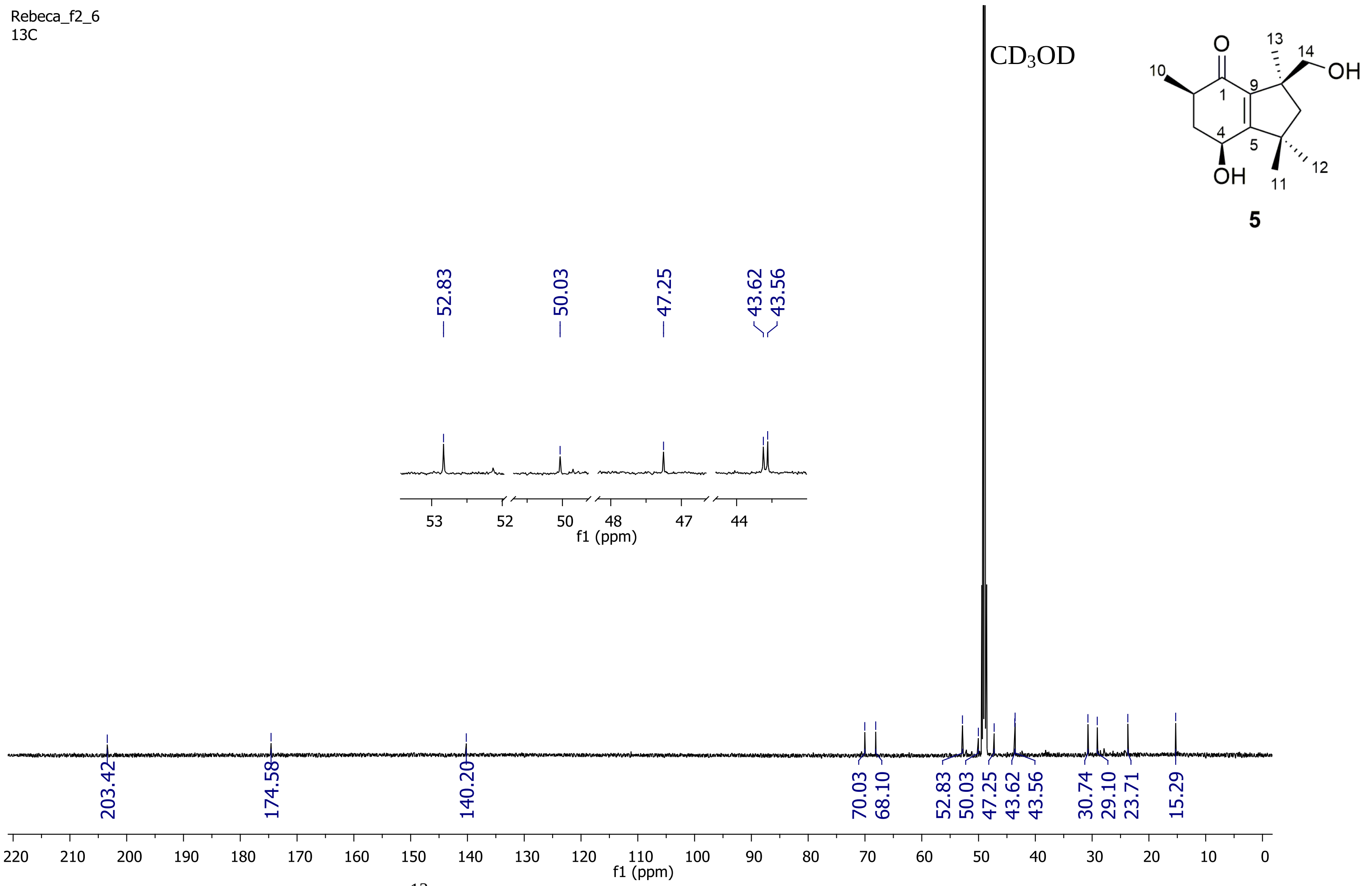

Figura S36 - ^{13}C NMR spectrum of compound **5** (CD_3OD; 150 MHz)

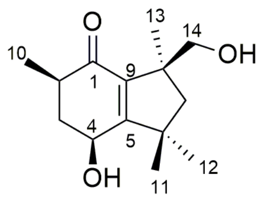

5

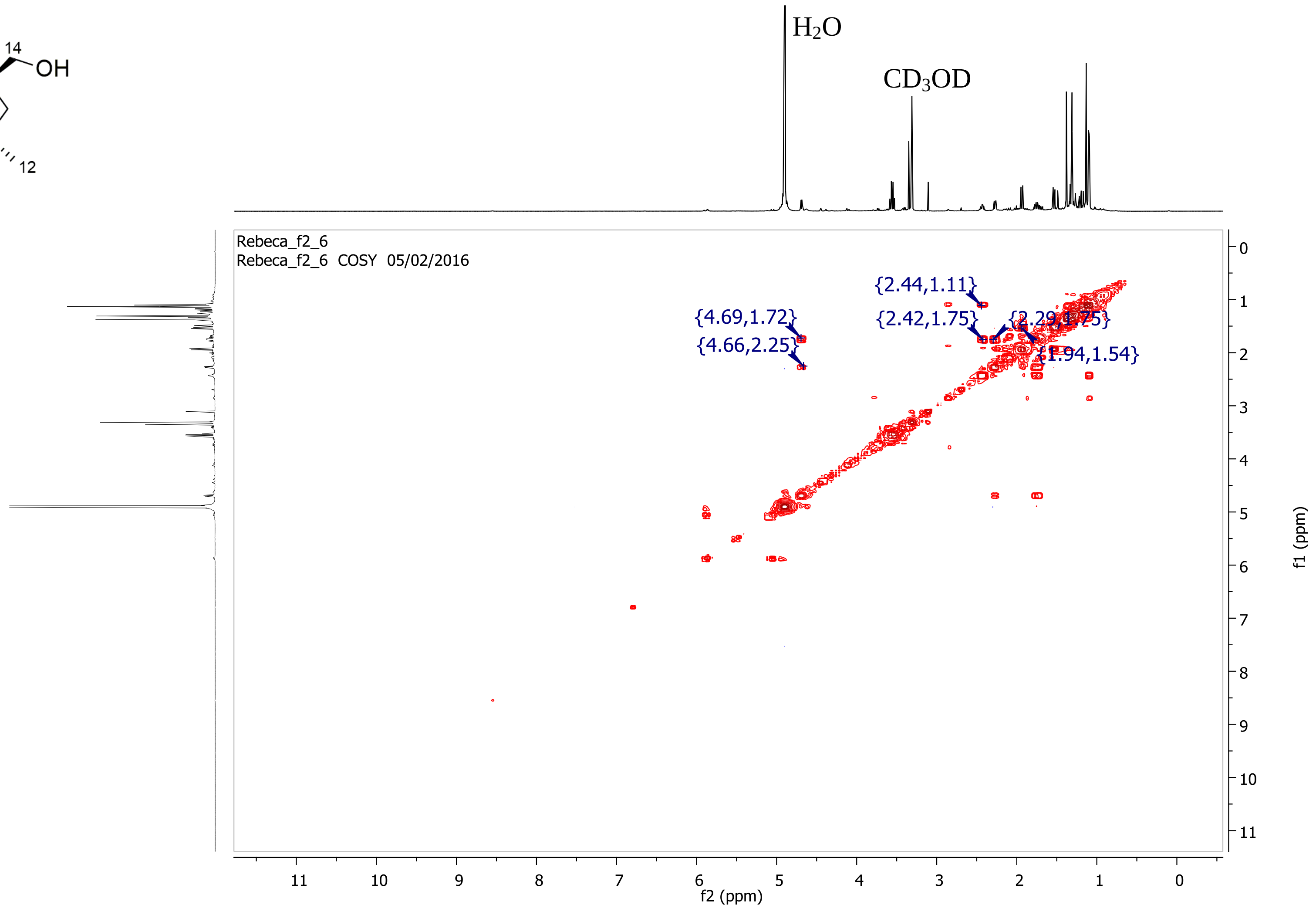

Figure S37 - ^{1}H–^{1}HCOSY spectrum of compound **5** (CD_3OD; 600 MHz)

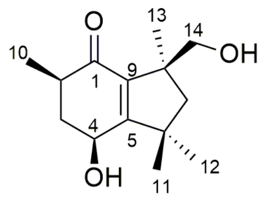

5

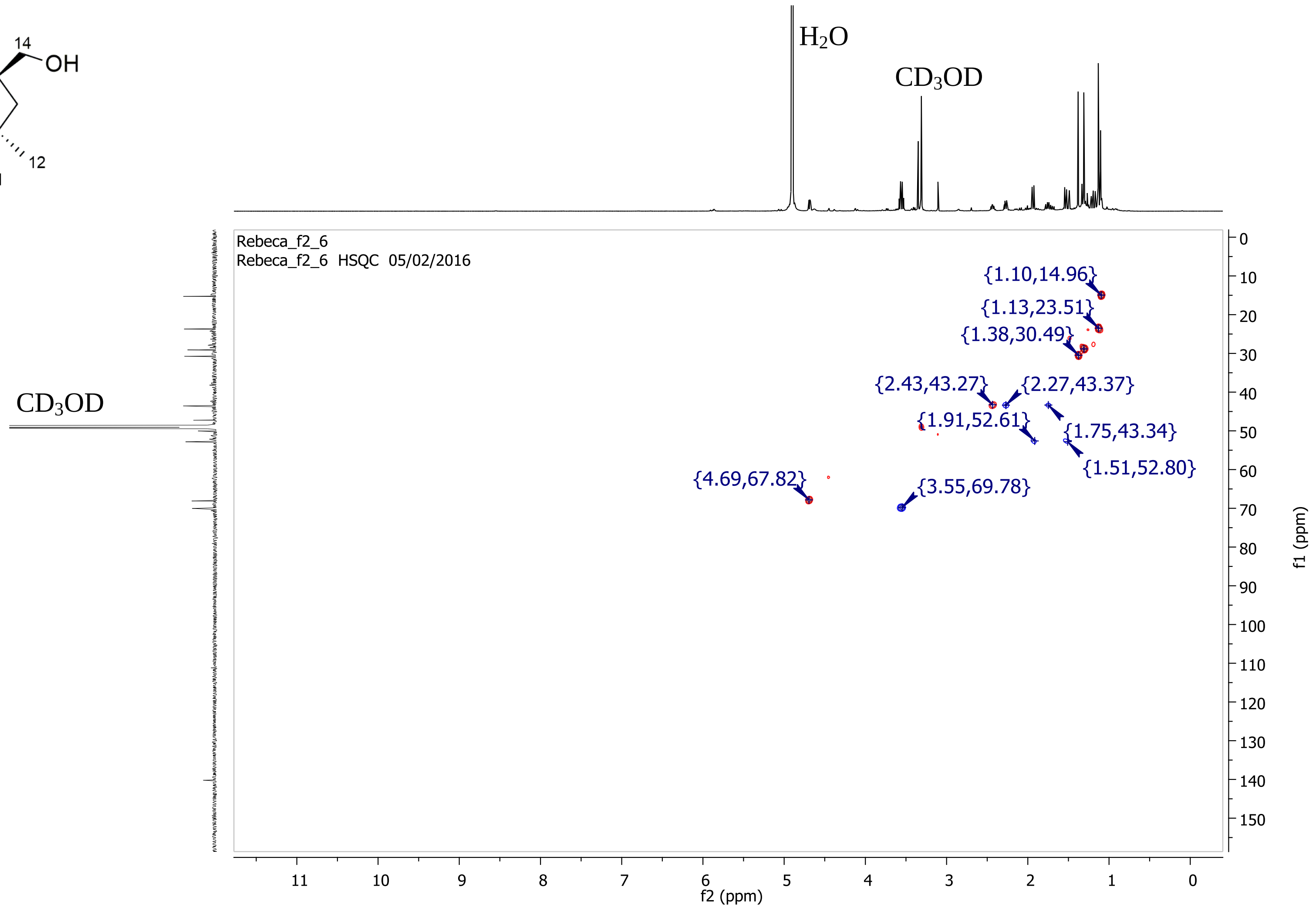

Figure S38 - HSQC spectrum of compound **5** (CD_3OD; 600 and 150 MHz)

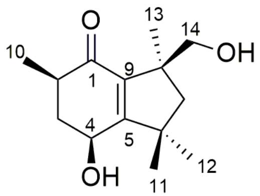

5

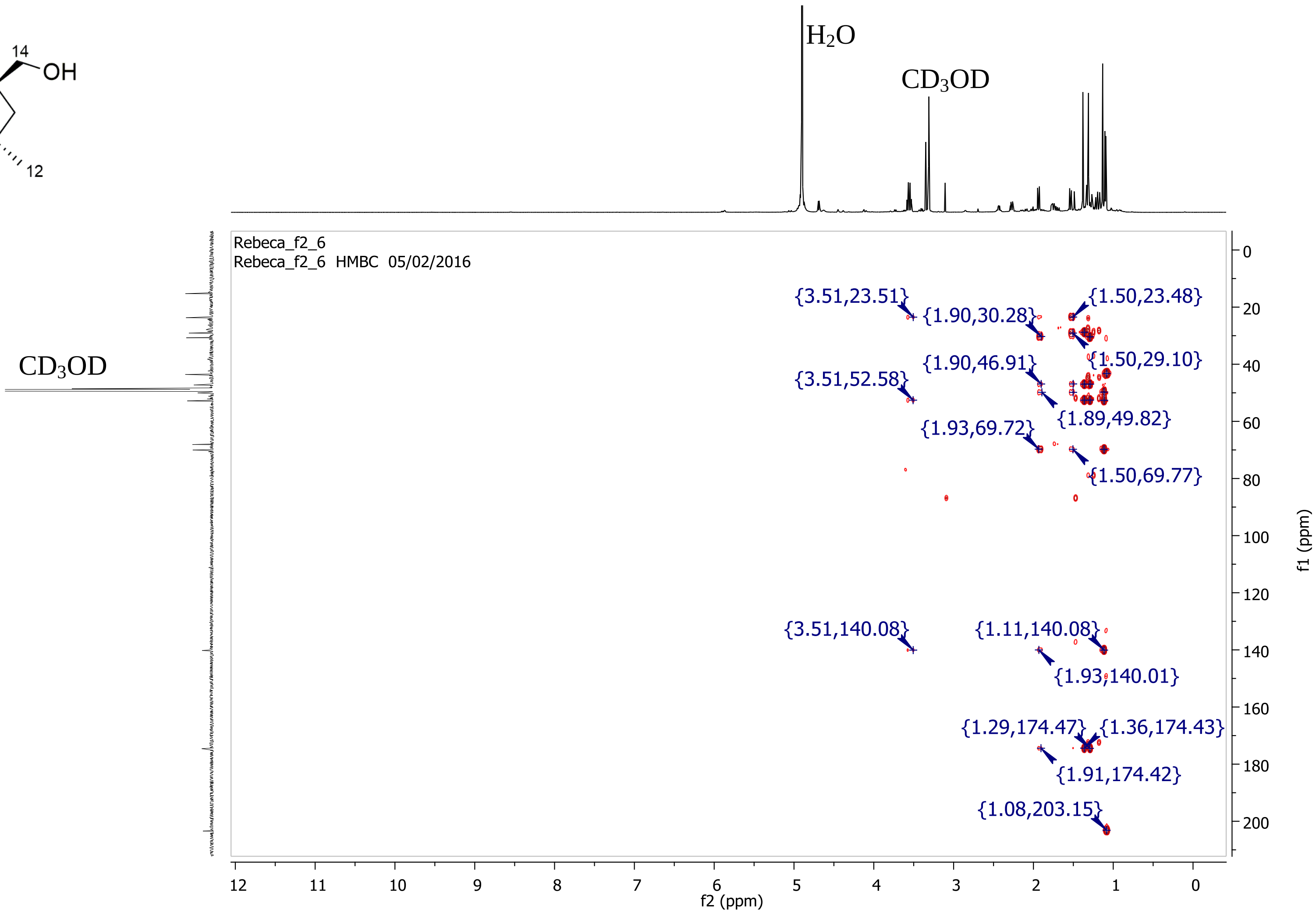

Figure S39 - HMBC spectrum of compound **5** (CD_3OD; 600 and 150 MHz)

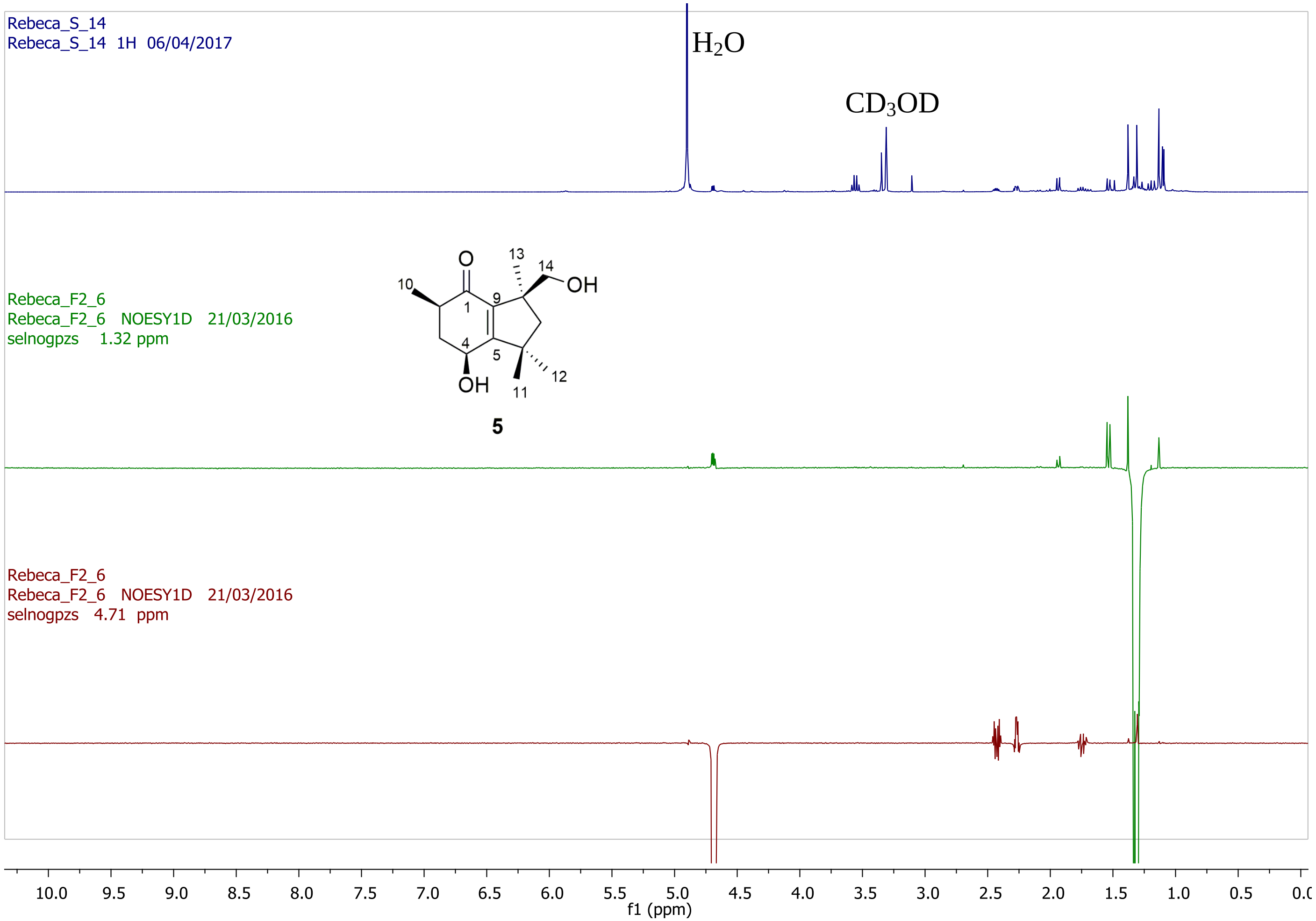

Figure S40 - ^{1}H NMR (blue) and NOESY 1D (green - selected signal: δ_H1.31; red - selected signal: δ_H 4.69) spectra of compound **5** - (CD_3OD; 600 MHz)

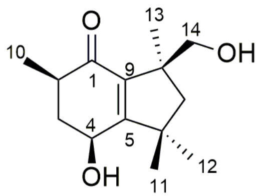

5

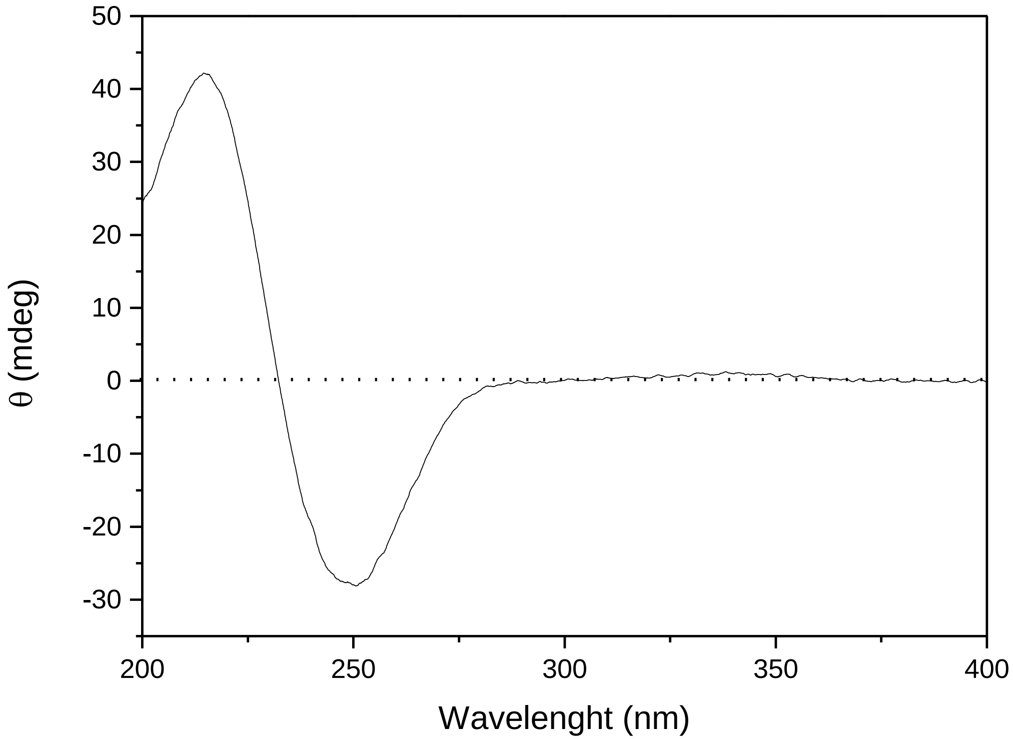

Figure S41 - CD spectrum of compound **5**

Acquisition Parameter

Source Type	ESI	Ion Polarity	Positive	Set Nebulizer	0.3 Bar
Focus	Active	Set Capillary	4500 V	Set Dry Heater	180 °C
Scan Begin	50 m/z	Set End Plate Offset	-500 V	Set Dry Gas	4.0 l/min
Scan End	1500 m/z	Set Charging Voltage	2000 V	Set Divert Valve	Source
		Set Corona	0 nA	Set APCI Heater	0 °C

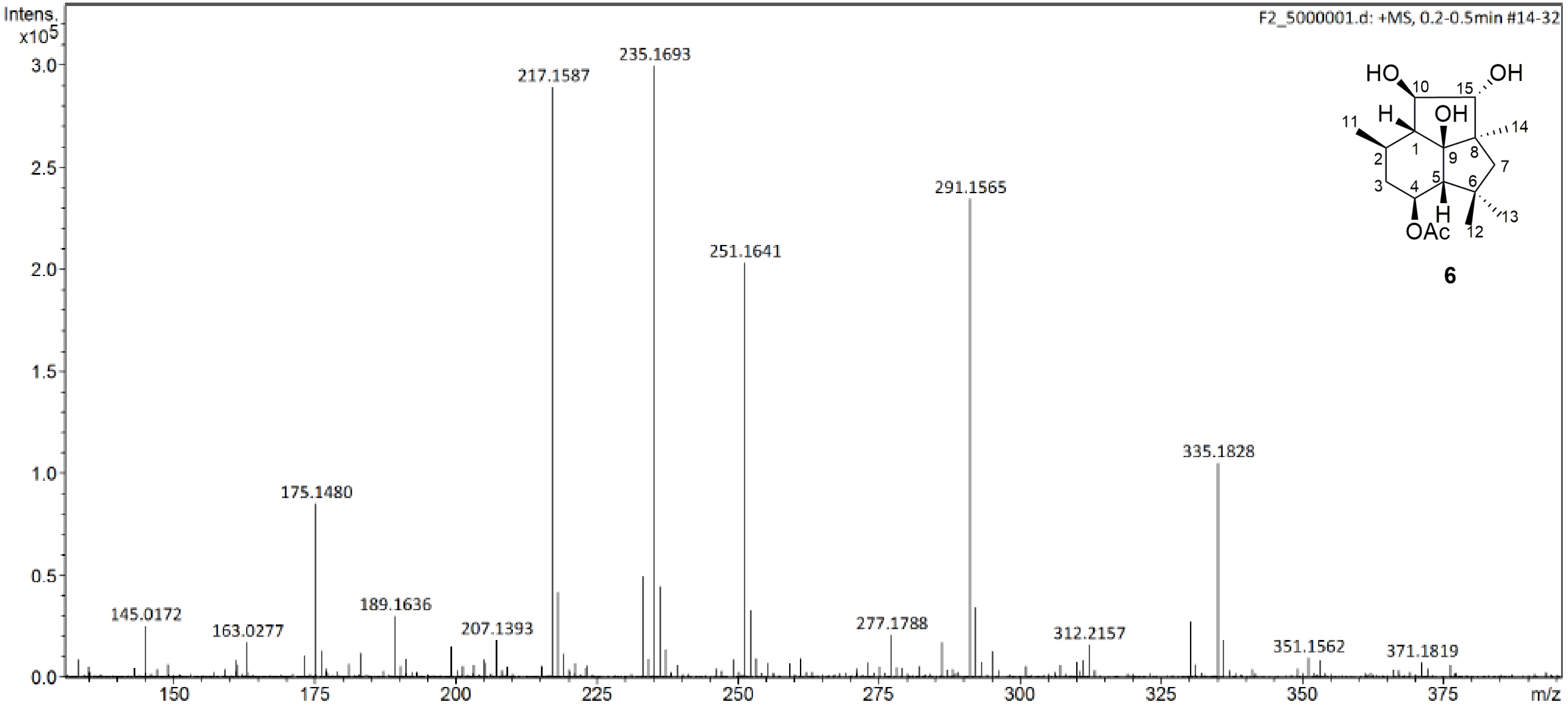

6

Figure S42 - High resolution ESI-Q-TOF-MS spectrum of compound **6** (Bruker - MaXix Impact)

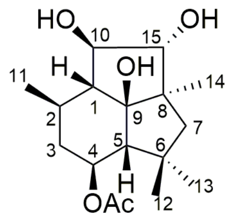

6

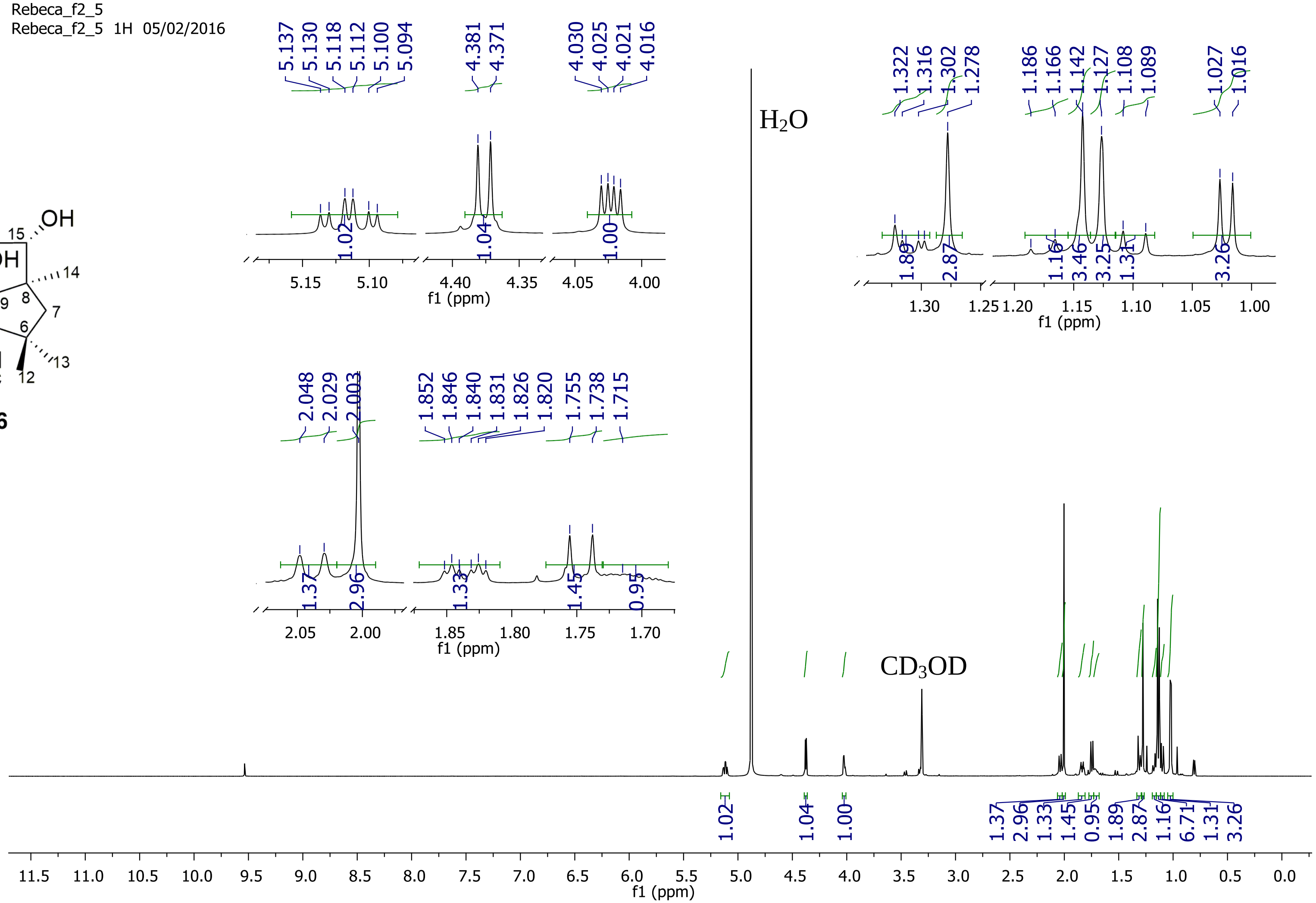

Figure S43 - ^{1}H NMR spectrum of compound **6** (CD_3OD; 600 MHz)

Rebeca_S_13
Rebeca_S_13 1H 06/04/2017

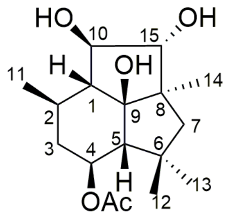

6

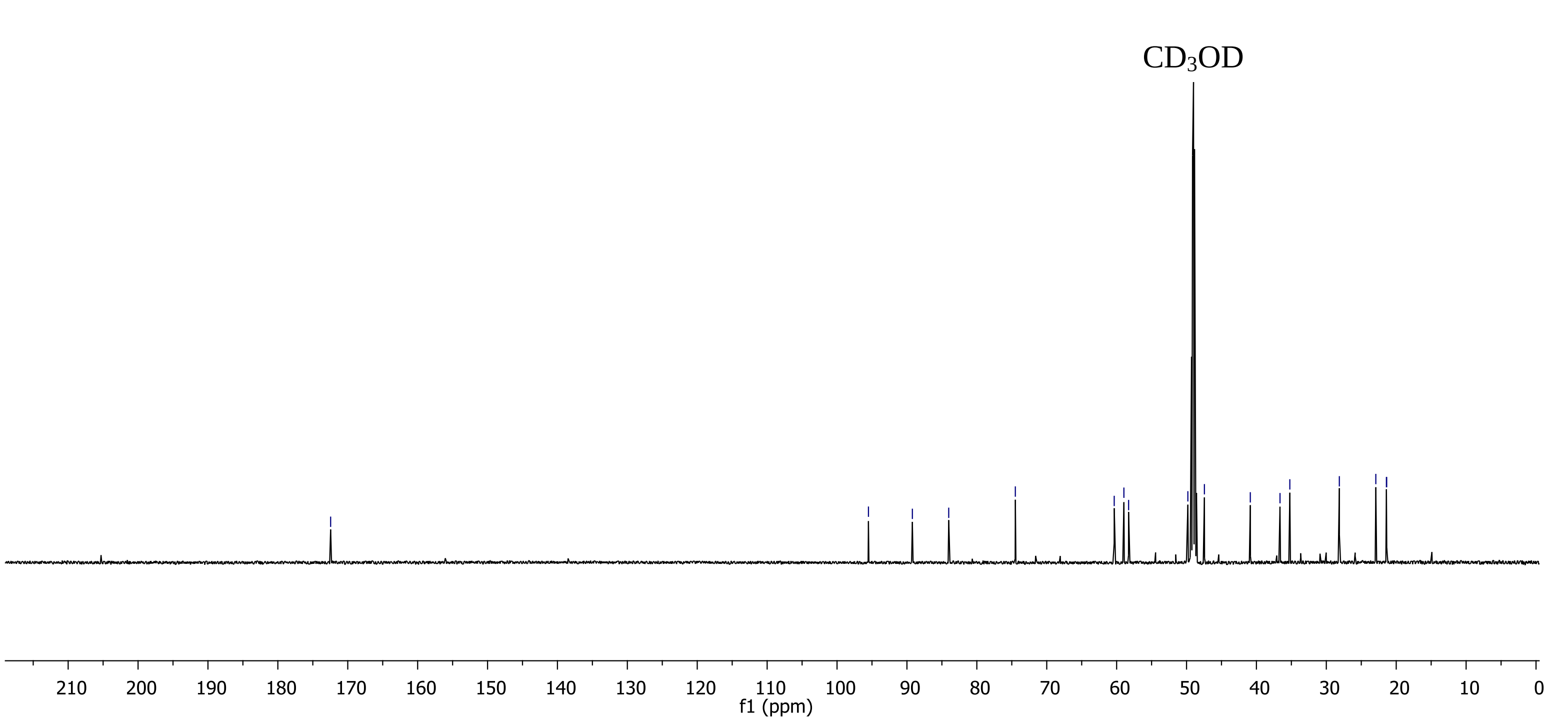

Figure S44 - ^{13}C NMR spectrum of compound **6** (CD_3OD; 600 MHz)

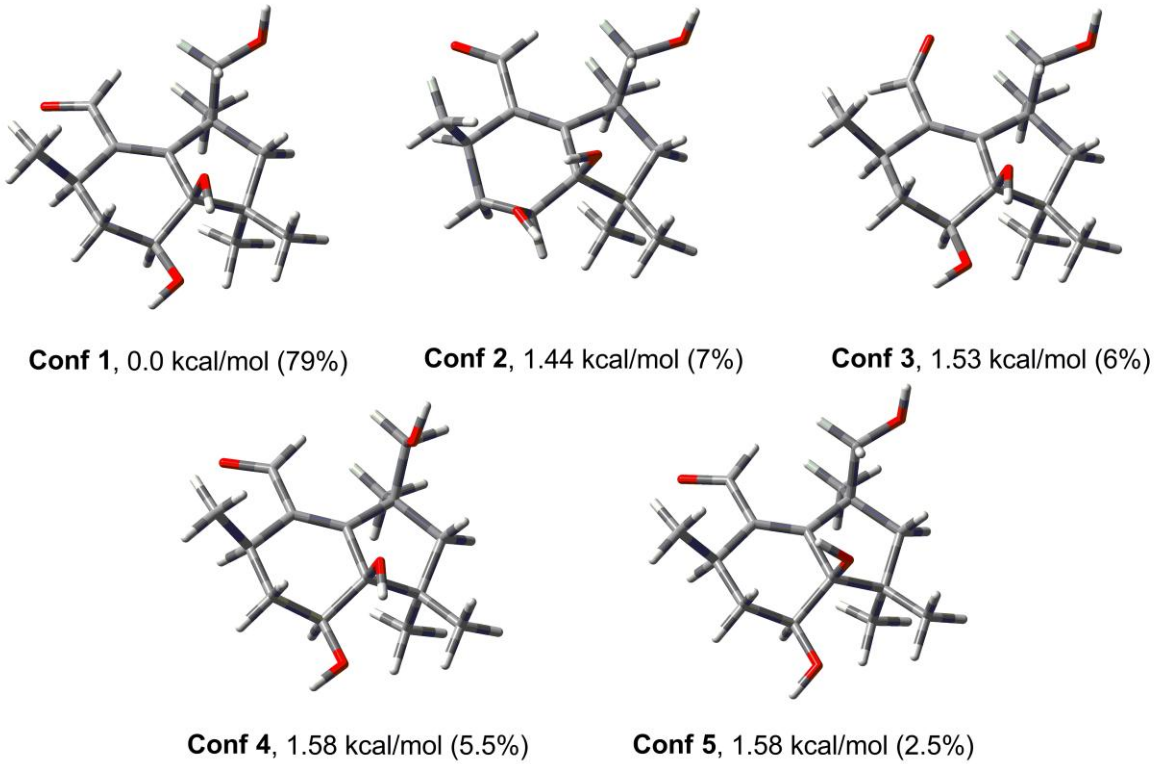

Figure S45–Structures, relative energies and Boltzmann factors of the lowest-energy conformers identified for (2*R*,4*S*,5*R*,8*S*)-**1** at the B3LYP/PCM(MeOH)/6-31G(d) level.

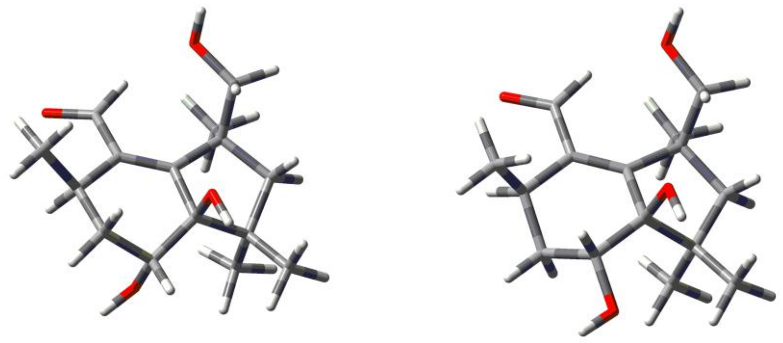

Figure S46–Structures of the lowest-energy conformers identified for (2*R*,4*R*,5*R*,8*S*)-**2** at the B3LYP/PCM(MeOH)/6-31G(d) level and used as simple average.

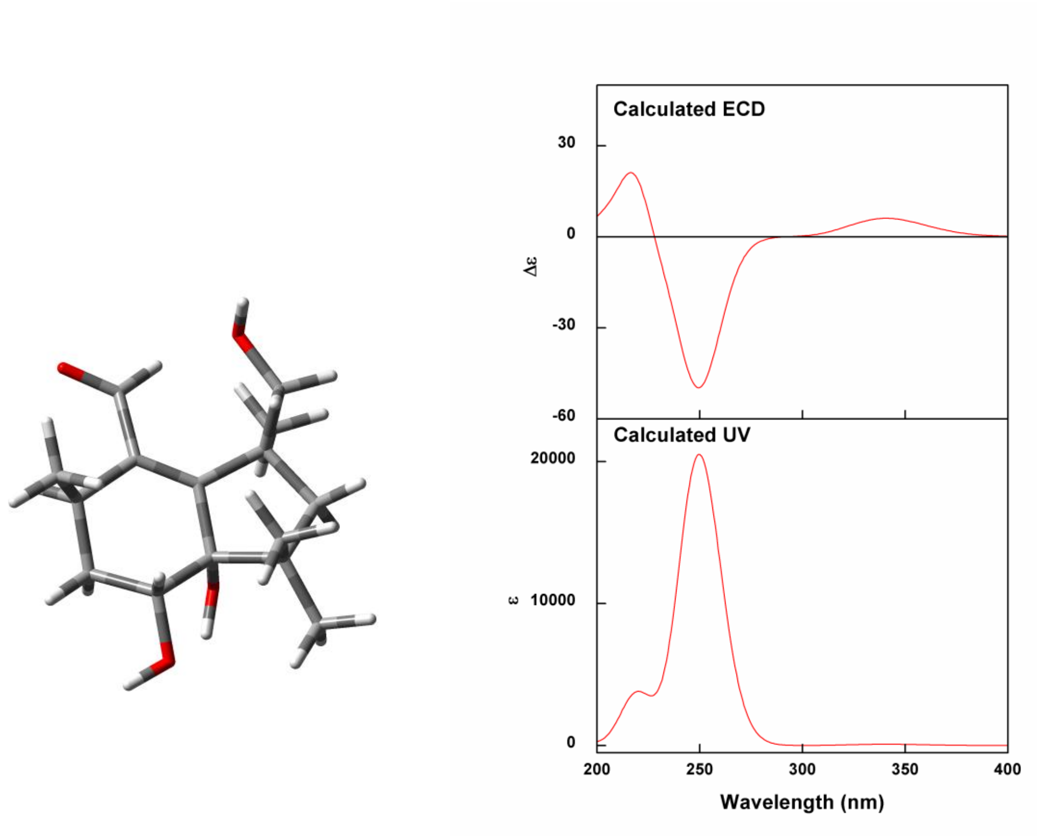

Figure S47 - Structure of the lowest-energy conformer identified for (2*R*,4*R*,5*S*,8*S*)-**2** at the B3LYP/PCM(MeOH)/6-31G(d) and its UV and ECD spectra calculated at the CAM-B3LYP/PCM(MeOH)/TZVP level

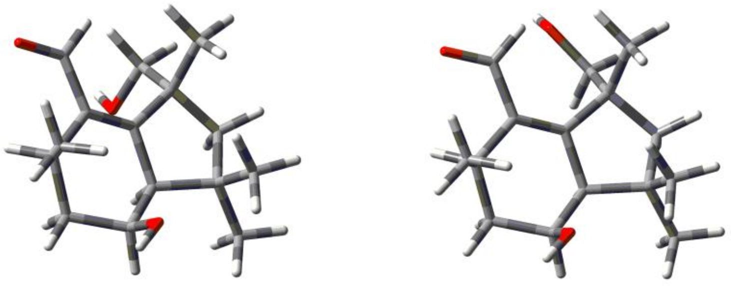

Figure S48–Structures of the lowest-energy conformers identified for (2*R*,4*S*,5*R*,8*R*)-**3** at the B3LYP/PCM(MeOH)/6-31G(d) level and used as simple average.

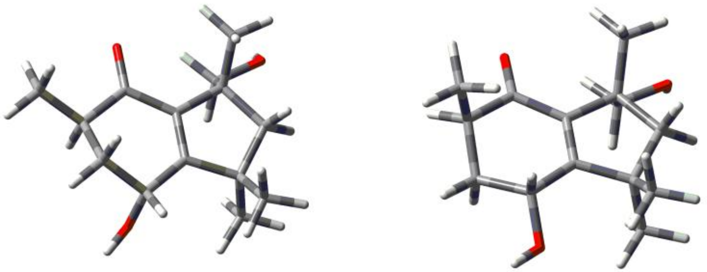

Figure S49–Structures of the lowest-energy conformers identified for (2*R*,4*R*,8*R*)-**4** at the B3LYP/PCM(MeOH)/6-31G(d) level and used as simple average.

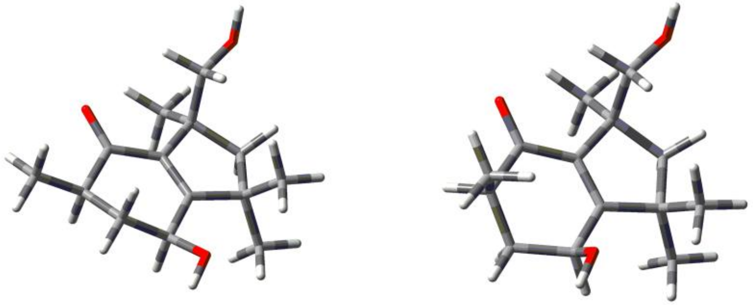

Conf 1, 0.0 kcal/mol (93%) **Conf 2**, 2.0 kcal/mol (7%)

Figure S50–Structures, relative energies and Boltzmann factors of the lowest-energy conformers identified for (2*R*,4*S*,8*S*)-**5** at the B3LYP/PCM(MeOH)/6-31G(d) level.

Table S2 - Cartesian coordinates of lowest-energy conformers

B3LYP/PCM(MeOH)/6-31G(d) opt

Total energy = -556336.5853 kcal mol^{-1}

Comp. 1 conformer 1

C	2.52814900	-0.56876000	0.11649100
C	1.89424600	-1.91828600	-0.26061900
C	1.50978500	0.57122800	0.19067100
C	0.17215100	0.39767900	0.03782600
C	-0.42541000	-0.99126000	-0.21737200
C	0.49294600	-2.10607300	0.30433600
C	-0.97705700	1.43016200	0.08384900
C	-2.25716800	0.53800800	0.02193200
C	-1.85740000	-0.90381700	0.41366000
C	2.07431100	1.89100000	0.55454400
O	3.18776500	2.04686900	1.03946700
C	-0.90345400	2.31512600	-1.18128400
O	-2.06958200	3.13441600	-1.22750700
C	-1.02736500	2.31795300	1.35169300
C	-2.85894800	-1.91712300	-0.16365300
C	-1.80979900	-1.07908900	1.94623900
O	-0.52544600	-1.12518600	-1.64945700
O	-0.08649700	-3.34123300	-0.14177200
C	3.67555400	-0.26619700	-0.87121500
H	2.97188000	-0.65144600	1.11876100
H	2.54843500	-2.73114000	0.08124600
H	1.82286200	-1.99493500	-1.35142000
H	0.52247500	-2.07938800	1.40109400
H	-2.65090300	0.53562300	-0.99925400
H	-3.05134900	0.92680100	0.66712200
H	1.43929500	2.77243800	0.37322400
H	0.00281800	2.93810400	-1.16247200
H	-0.84337400	1.66532200	-2.06548700
H	-2.06129700	3.60421300	-2.07590800
H	-0.19944100	3.02848100	1.41184100
H	-1.95358600	2.89939400	1.34055300
H	-1.01781100	1.71381900	2.26314700
H	-2.91429300	-1.84246100	-1.25399000
H	-2.58833300	-2.94324100	0.10123600
H	-3.86045300	-1.71404200	0.23526400
H	-2.77348600	-0.79409900	2.38395200
H	-1.61931300	-2.12362400	2.21609700
H	-1.03606700	-0.46811300	2.42343000
H	-0.65198100	-2.08166100	-1.79600000
H	0.59235900	-4.03171800	-0.07270200
H	3.28077200	-0.10355400	-1.88130100
H	4.24739200	0.61425200	-0.57390400
H	4.36023700	-1.12127000	-0.91296800

Table S3 - Cartesian coordinates of lowest-energy conformers

B3LYP/PCM(MeOH)/6-31G(d) opt

Total energy = -556335.1388 kcal mol^{-1}

Comp. 1 conformer 2

C	2.47245200	0.44595600	0.36867700
C	1.12124000	1.13992100	0.26099100
C	-0.02447400	0.43688400	0.08024300
C	-0.02112800	-1.06922700	-0.22733200
C	1.31176900	-1.83683200	0.09689000
C	-1.49685200	0.89815700	0.10785900
C	-2.29658100	-0.44991500	0.15603500
C	-1.29743200	-1.58183100	0.50188900
C	1.15703100	2.60898200	0.36462200
O	2.17769000	3.25072500	0.59000000
C	-1.83681600	1.66179000	-1.19191500
O	-3.24011800	1.92315000	-1.20920900
C	-1.86659100	1.76343700	1.33681900
C	-1.77846200	-2.94504100	-0.01250900
C	-1.07243500	-1.66545100	2.02635900
O	-0.27484300	-1.15664200	-1.64101000
O	1.96166700	-2.24356900	-1.12715500
C	3.27847400	0.53973900	-0.94376200
H	3.04160600	1.00376600	1.12219000
H	1.07517800	-2.74041900	0.66665400
H	-2.74807000	-0.64588000	-0.82059600
H	-3.11491600	-0.39941800	0.88162700
H	0.20944800	3.14674500	0.19674500
H	-1.27251500	2.60515700	-1.23603300
H	-1.53879200	1.04800800	-2.05071500
H	-3.45513600	2.28948300	-2.08094600
H	-1.37398800	2.73929400	1.33425700
H	-2.94589800	1.94106300	1.33536000
H	-1.60708700	1.25889500	2.27222500
H	-1.91928800	-2.92980200	-1.09582200
H	-1.06645800	-3.74425000	0.23105300
H	-2.73375500	-3.20764400	0.45778000
H	-2.02573100	-1.86296500	2.52962700
H	-0.38949300	-2.48042900	2.29285500
H	-0.66264000	-0.74030500	2.44466200
H	0.59743900	-1.37418800	-2.02919800
H	1.64065300	-3.13089700	-1.35407100
H	2.75527500	0.06182300	-1.77614700
H	3.45508000	1.58862400	-1.20221000
H	4.25212200	0.04832700	-0.83111800
C	2.29257500	-0.98493800	0.89942800
H	1.91987200	-0.92692300	1.92993200
H	3.25530600	-1.50717000	0.93511100

Table S4 - Cartesian coordinates of lowest-energy conformers

B3LYP/PCM(MeOH)/6-31G(d) opt

Total energy = -556335.0522 kcal mol^{-1}

Comp. 1 conformer 3

C	1.45137900	2.20159700	0.28295600
C	2.48144000	1.19765500	-0.24950600
C	0.02630200	1.63260500	0.30534500
C	-0.26245800	0.32883000	0.08039100
C	0.84288200	-0.69060000	-0.23168500
C	2.22283400	-0.21696600	0.25062900
C	-1.61501600	-0.41233000	0.11414000
C	-1.20245300	-1.91618300	0.03401200
C	0.29874700	-2.02247600	0.39024400
C	-0.96287500	2.66174400	0.72291000
O	-2.11996700	2.78765400	0.35414300
C	-2.42248400	-0.02681900	-1.14559600
O	-3.57586300	-0.87067700	-1.21841300
C	-2.46346200	-0.16264400	1.38142400
C	0.91205400	-3.29411000	-0.21725900
C	0.50792400	-2.05365200	1.91885700
O	0.88956700	-0.80087900	-1.66874600
O	3.17484100	-1.14586000	-0.28836900
C	1.53690300	3.50609300	-0.53736100
H	1.71184500	2.44836300	1.32470400
H	3.48836500	1.52365400	0.04286100
H	2.44639300	1.18142500	-1.34485600
H	2.26195000	-0.23530800	1.34734400
H	-1.35794100	-2.28301300	-0.98506000
H	-1.81674700	-2.53875500	0.69275400
H	-0.54657800	3.39570900	1.44556000
H	-2.70968900	1.02818500	-1.08285100
H	-1.78629500	-0.16538800	-2.03162400
H	-4.05828700	-0.62686100	-2.02343900
H	-2.77720100	0.87752200	1.47440800
H	-3.36494800	-0.78020600	1.32805700
H	-1.91611100	-0.44240000	2.28678500
H	0.79887500	-3.30632700	-1.30563700
H	1.97584500	-3.38200200	0.02157800
H	0.39877600	-4.17809000	0.18106500
H	-0.07303100	-2.87299100	2.35794800
H	1.56047100	-2.22364900	2.17129500
H	0.19435000	-1.12551900	2.40924200
H	1.75036000	-1.22272900	-1.85217700
H	4.05467400	-0.73929300	-0.23403200
H	1.24808900	3.32604100	-1.57929100
H	0.89297600	4.29769200	-0.13979100
H	2.56494000	3.88559700	-0.53075400

Table S5 - Cartesian coordinates of lowest-energy conformers

B3LYP/PCM(MeOH)/6-31G(d) opt

Total energy = -556334.9989 kcal mol^{-1}

Comp. 1 conformer 4

C	-2.07738000	-1.42275000	-0.18129100
C	-0.96193000	-2.43733500	0.12374100
C	-1.58298500	0.02275700	-0.24219400
C	-0.28777800	0.39078600	-0.06737600
C	0.79858100	-0.66408300	0.19851000
C	0.40193000	-2.01970600	-0.41150800
C	0.36636200	1.78812900	-0.18350700
C	1.87999000	1.47686400	0.00288900
C	2.11995500	-0.01038900	-0.34758300
C	-2.61458700	1.01175200	-0.62337500
O	-3.70973700	0.71546000	-1.08562800
C	-0.09353300	2.78437000	0.90531500
O	0.04698700	2.20960800	2.19842800
C	0.13033900	2.47665700	-1.55351800
C	3.39399100	-0.52255700	0.34595000
C	2.28064800	-0.23011400	-1.86669400
O	0.88230600	-0.83535400	1.62509900
O	1.41948100	-2.95109800	-0.01377700
C	-3.21925100	-1.60744400	0.84099900
H	-2.49232300	-1.63994400	-1.17556700
H	-1.24116700	-3.41618200	-0.28769000
H	-0.86470700	-2.55650000	1.20820200
H	0.37218100	-1.94185400	-1.50564100
H	2.14009700	1.63301500	1.05407400
H	2.50577600	2.14637500	-0.59787600
H	-2.36482600	2.07483200	-0.47986700
H	0.52935300	3.68738000	0.80801500
H	-1.13443900	3.09242600	0.73206800
H	-0.13333600	2.90916800	2.84549100
H	-0.90611800	2.79552300	-1.69310000
H	0.76146700	3.36990000	-1.62409200
H	0.38927600	1.82125400	-2.38766800
H	3.33588100	-0.39268800	1.43042700
H	3.56863500	-1.58091500	0.13077200
H	4.26148800	0.04475800	-0.01321200
H	3.08893000	0.40236500	-2.25200000
H	2.54103200	-1.27253600	-2.07965800
H	1.37655300	0.00528600	-2.43644200
H	1.37620300	-1.66948500	1.73590300
H	1.07220000	-3.84780800	-0.14767400
H	-2.87878700	-1.34115000	1.84871900
H	-4.08843400	-0.99601800	0.59226400
H	-3.53333800	-2.65773400	0.85943700

Table S6 - Cartesian coordinates of lowest-energy conformers

B3LYP/PCM(MeOH)/6-31G(d) opt

Total energy = -556334.5302 kcal mol^{-1}

Comp. 1 conformer 5

C	2.53408800	-0.55690200	0.10058100
C	1.90801100	-1.91566100	-0.26525600
C	1.50775200	0.57804700	0.18787600
C	0.16680200	0.39354900	0.05551100
C	-0.41710200	-0.99406600	-0.24169900
C	0.50463400	-2.09598700	0.30199700
C	-0.99240300	1.41392900	0.11880200
C	-2.26731000	0.51720000	0.01236100
C	-1.86247800	-0.94098900	0.33962200
C	2.06536400	1.89946500	0.55389600
O	3.18547200	2.06261800	1.02108600
C	-0.92335000	2.35001000	-1.10951800
O	-2.09814200	3.15734400	-1.12625600
C	-1.05121700	2.24776300	1.42208200
C	-2.83383800	-1.93813800	-0.31377400
C	-1.85992400	-1.18985800	1.86231200
O	-0.56983400	-1.09639300	-1.67519000
O	-0.04332500	-3.36995000	-0.03705000
C	3.67834100	-0.24684500	-0.88810300
H	2.98127700	-0.63951000	1.10128400
H	2.56124000	-2.72049100	0.09410600
H	1.85853200	-2.03257600	-1.35666700
H	0.54947900	-1.98490200	1.39462000
H	-2.66360800	0.56254800	-1.00632700
H	-3.06193900	0.87156900	0.67669400
H	1.41610200	2.77544800	0.39754300
H	-0.02519700	2.98281300	-1.06683100
H	-0.85612900	1.73412100	-2.01737500
H	-2.08656100	3.67089500	-1.94888300
H	-0.20598100	2.93182000	1.53105700
H	-1.96292800	2.85182300	1.41576200
H	-1.07677900	1.60458400	2.30616900
H	-2.86617300	-1.80880900	-1.39838700
H	-2.54272300	-2.96930100	-0.09710800
H	-3.84498900	-1.77392900	0.07956500
H	-2.85124800	-0.96522900	2.27195800
H	-1.63459200	-2.23756100	2.08683900
H	-1.13291700	-0.56941300	2.39712800
H	0.27630500	-0.85227800	-2.08278800
H	0.53336000	-4.04197800	0.36086300
H	3.28572300	-0.09195900	-1.90059500
H	4.24145400	0.64011500	-0.59402000
H	4.37068700	-1.09585600	-0.92546600

Table S7 - Cartesian coordinates of lowest-energy conformers

B3LYP/PCM(MeOH)/6-31G(d) opt

Total energy = -556332.4845 kcal mol^{-1}

Comp. 2 conformer 1

C	-1.92881500	-1.52786700	0.02756200
C	-0.85548600	-2.45457000	0.62518300
C	-1.43642500	-0.09779700	-0.20808100
C	-0.15343500	0.29441300	-0.00189400
C	0.91443600	-0.72024600	0.41409000
C	0.53498200	-2.17412500	0.06496100
C	0.50977500	1.69001200	-0.07981800
C	2.02585500	1.37684200	0.14618800
C	2.25158100	-0.12430200	-0.15105400
C	-2.43689900	0.81953800	-0.80204900
O	-3.47630600	0.43278900	-1.32838000
C	0.06378600	2.58053400	1.10342000
O	-1.27438900	3.03469600	0.93269700
C	0.32477700	2.45416300	-1.41207700
C	2.44874900	-0.34267800	-1.66713600
C	3.50048100	-0.66148700	0.56890700
O	0.93645900	-0.63453500	1.86176500
O	0.55530200	-2.37058600	-1.34987900
C	-3.17306000	-1.56292500	0.94103200
H	-2.22911000	-1.92014300	-0.95296400
H	-0.81976200	-2.32879600	1.71190900
H	-1.13134500	-3.50071600	0.43525200
H	1.27387400	-2.83880100	0.54363700
H	2.66204400	2.01333500	-0.47718100
H	2.29163800	1.57944100	1.18960300
H	-2.21143600	1.89175600	-0.76022100
H	0.75396500	3.43791400	1.15462100
H	0.16836600	2.00642600	2.03333700
H	-1.54007200	3.48342100	1.75023000
H	0.59227700	1.83302400	-2.27116000
H	0.98215800	3.33161300	-1.41642700
H	-0.69495100	2.81270900	-1.55631100
H	2.71637600	-1.37770300	-1.88774700
H	3.25967400	0.30819900	-2.01644100
H	1.55262400	-0.11372200	-2.24834700
H	3.49357800	-0.44021800	1.64161000
H	4.39937500	-0.19280700	0.15193000
H	3.60889000	-1.74510500	0.43463700
H	1.63873400	-1.22705100	2.18023800
H	0.20378000	-3.26050400	-1.51453900
H	-3.47922600	-2.60244800	1.10906600
H	-4.01510700	-1.02586600	0.50159300
H	-2.94668900	-1.11990200	1.91863400

Table S8 - Cartesian coordinates of lowest-energy conformers

B3LYP/PCM(MeOH)/6-31G(d) opt

Total energy = -556331.9160 kcal mol^{-1}

Comp. 2 conformer 2

C	1.05026500	2.21855200	-0.27669300
C	1.35035400	0.72516200	-0.25110100
C	0.39687900	-0.20157900	0.01160200
C	-1.02508600	0.18548800	0.45676500
C	-1.32656900	1.70619000	0.38534600
C	0.43254200	-1.73712500	-0.15926900
C	-1.08494800	-2.11792000	-0.20468600
C	-1.91678400	-0.81794700	-0.35611100
C	2.76014800	0.37610800	-0.54265500
O	3.59200500	1.20561400	-0.89895700
C	1.05355800	-2.45837100	1.05777800
O	2.45975500	-2.23096900	1.11724100
C	1.11838900	-2.21870000	-1.45923500
C	-2.02739900	-0.40639700	-1.83832500
C	-3.33154800	-1.03295200	0.20907800
O	-1.05066500	-0.19801800	1.85806700
O	-2.71933600	1.91064300	0.12160800
C	1.52134000	2.92761100	1.01135600
H	1.64792300	2.63253600	-1.09668400
H	-1.10342900	2.08312000	1.39223900
H	-1.29411300	-2.81772600	-1.02064500
H	-1.36929000	-2.61832900	0.72689500
H	3.06199000	-0.66404900	-0.36565700
H	0.84569600	-3.53487900	0.94902400
H	0.55935400	-2.10536700	1.97000200
H	2.78437200	-2.60951600	1.94889400
H	0.70429700	-1.71185000	-2.33624700
H	0.94013500	-3.29364000	-1.57982800
H	2.19842800	-2.06806900	-1.45534400
H	-2.61009700	0.51245500	-1.94214900
H	-2.53022200	-1.19899700	-2.40518400
H	-1.04826900	-0.24228800	-2.30044300
H	-3.30672000	-1.32984900	1.26391100
H	-3.81964400	-1.84932700	-0.33762000
H	-3.94587300	-0.13613600	0.11056600
H	-1.94569100	-0.00156300	2.18239100
H	-2.88587700	2.86310800	0.20885400
H	1.30955600	4.00217400	0.95766600
H	2.60108200	2.80267100	1.13880000
H	1.03065400	2.52722500	1.90488400
C	-0.43242000	2.45357600	-0.59862200
H	-0.66295800	3.52644100	-0.54794200
H	-0.64770100	2.12352100	-1.62143000

Table S9 - Cartesian coordinates of lowest-energy conformers

B3LYP/PCM(MeOH)/6-31G(d) opt

Total energy = -509144.9869 kcal mol^{-1}

Comp. 3 conformer 1

C	-1.46730000	-1.58190000	0.32400000
C	-1.53460000	-0.05860000	0.36630000
C	-0.42370000	0.70950000	0.32310000
C	0.95530000	0.09840000	0.16140000
C	1.05740000	-1.38840000	0.50490000
C	-0.24710000	2.23450000	0.31930000
C	1.29810000	2.40720000	0.43760000
C	1.89760000	1.06580000	0.90260000
C	-2.77360000	0.49080000	0.44860000
O	-3.80110000	-0.12740000	0.60000000
C	-0.66660000	2.86600000	-1.02740000
O	0.26540000	2.60760000	-2.04010000
C	-0.88230000	2.98960000	1.50660000
C	1.85700000	0.96570000	2.43920000
C	3.35750000	0.91620000	0.43640000
O	1.01090000	-1.61370000	1.88620000
C	-1.87190000	-2.18520000	1.68410000
H	-2.21310000	-1.94330000	-0.42700000
H	2.03790000	-1.79510000	0.16100000
H	1.61330000	3.26000000	1.08190000
H	1.72490000	2.63460000	-0.56690000
H	-2.88490000	1.58510000	0.36670000
H	-1.63260000	2.44090000	-1.38550000
H	-0.75370000	3.97500000	-0.95730000
H	-0.05180000	3.00820000	-2.83090000
H	-0.62150000	2.52470000	2.48250000
H	-0.53040000	4.04600000	1.53670000
H	-1.98800000	3.07040000	1.46950000
H	0.82060000	0.87350000	2.83160000
H	2.43630000	0.09210000	2.81290000
H	2.31860000	1.86280000	2.91100000
H	3.99810000	1.71350000	0.87790000
H	3.77950000	-0.06760000	0.74320000
H	3.45290000	0.98270000	-0.67130000
H	1.16180000	-2.53180000	2.03150000
H	-2.94000000	-1.99030000	1.93080000
H	-1.74660000	-3.29220000	1.69170000
H	-1.27440000	-1.76550000	2.52340000
C	-0.10890000	-2.11760000	-0.16120000
H	-0.02520000	-1.96520000	-1.26430000
H	-0.03170000	-3.21790000	0.00750000
H	1.18640000	0.17950000	-0.90860000

Table S10 - Cartesian coordinates of lowest-energy conformers

B3LYP/PCM(MeOH)/6-31G(d) opt

Total energy = -509144.3029 kcal mol^{-1}

Comp. 3 conformer 2

C	-1.46730000	-1.58190000	0.32400000
C	-1.53460000	-0.05860000	0.36630000
C	-0.42370000	0.70950000	0.32310000
C	0.95530000	0.09840000	0.16140000
C	1.05740000	-1.38840000	0.50490000
C	-0.24710000	2.23450000	0.31930000
C	1.29810000	2.40720000	0.43760000
C	1.89760000	1.06580000	0.90260000
C	-2.77360000	0.49080000	0.44860000
O	-3.81060000	-0.12970000	0.47280000
C	-0.66660000	2.86600000	-1.02740000
O	-2.05480000	2.83950000	-1.20880000
C	-0.88230000	2.98960000	1.50660000
C	1.85700000	0.96570000	2.43920000
C	3.35750000	0.91620000	0.43640000
O	1.01090000	-1.61370000	1.88620000
C	-1.87190000	-2.18520000	1.68410000
H	-2.21310000	-1.94330000	-0.42700000
H	2.03790000	-1.79510000	0.16100000
H	1.61330000	3.26000000	1.08190000
H	1.72490000	2.63460000	-0.56690000
H	-2.87530000	1.58830000	0.49070000
H	-0.37260000	3.94000000	-1.07510000
H	-0.22680000	2.32310000	-1.89590000
H	-2.24370000	3.24490000	-2.03730000
H	-0.62150000	2.52470000	2.48250000
H	-0.53040000	4.04600000	1.53670000
H	-1.98800000	3.07040000	1.46950000
H	0.82060000	0.87350000	2.83160000
H	2.43630000	0.09210000	2.81290000
H	2.31860000	1.86280000	2.91100000
H	3.99810000	1.71350000	0.87790000
H	3.77950000	-0.06760000	0.74320000
H	3.45290000	0.98270000	-0.67130000
H	1.16180000	-2.53180000	2.03150000
H	-2.94000000	-1.99030000	1.93080000
H	-1.74660000	-3.29220000	1.69170000
H	-1.27440000	-1.76550000	2.52340000
C	-0.10890000	-2.11760000	-0.16120000
H	-0.02520000	-1.96520000	-1.26430000
H	-0.03170000	-3.21790000	0.00750000
H	1.18640000	0.17950000	-0.90860000

Table S11 - Cartesian coordinates of lowest-energy conformers

B3LYP/PCM(MeOH)/6-31G(d) opt

Total energy = -484502.8022 kcal mol^{-1}

Comp. 4 conformer 1

C	-2.56735900	-0.65909000	0.24047300
C	-2.63451100	0.69855700	-0.47462900
C	-1.19992500	-1.32125800	0.03792600
C	-0.04106500	-0.42187600	-0.14754700
C	-0.16256000	0.92323500	-0.15317900
C	-1.49369000	1.62893600	-0.04718300
C	1.40639400	-0.88649600	-0.29853400
C	2.14721400	0.45687800	-0.58977100
C	1.18012800	1.63462700	-0.25205900
O	-1.07516100	-2.54477900	0.07921400
C	1.59039800	-1.88772100	-1.45671300
C	1.88470100	-1.52205800	1.02258100
O	3.26800400	-1.86010400	0.90119800
C	1.54116200	2.30223100	1.09697400
C	1.19758300	2.70718600	-1.35972200
O	-1.63813500	2.05424800	1.32162000
C	-3.71970000	-1.59306500	-0.13895400
H	-2.62155400	-0.45463200	1.32166000
H	-2.58183800	0.55037900	-1.56184800
H	-3.59615100	1.18534700	-0.26773200
H	-1.48647400	2.51315300	-0.69944200
H	3.08503900	0.52237100	-0.03013900
H	2.40996300	0.49742700	-1.65314100
H	1.21273000	-1.46323600	-2.39407300
H	1.05259800	-2.81884000	-1.25966600
H	2.65212300	-2.11795100	-1.58893800
H	1.28138100	-2.41553000	1.23186600
H	1.73371900	-0.81193300	1.84977900
H	3.54008600	-2.26591200	1.73882000
H	1.57995300	1.56813900	1.90891600
H	0.80554900	3.06406800	1.37081700
H	2.52627000	2.77840000	1.02124400
H	2.21341700	3.10257600	-1.47786000
H	0.54106800	3.55191700	-1.12117900
H	0.88463400	2.29078400	-2.32432000
H	-2.40411400	2.65128500	1.35321500
H	-3.71776400	-1.80223200	-1.21544900
H	-4.68104300	-1.13380700	0.11511700
H	-3.63987600	-2.54719300	0.38811900

Table S12 - Cartesian coordinates of lowest-energy conformers

B3LYP/PCM(MeOH)/6-31G(d) opt

Total energy = -484501.6570 kcal mol^{-1}

Comp. 4 conformer 2

C	-2.23366800	-1.60259600	0.31687400
C	-0.71243600	-1.75155100	0.26858300
C	0.07056800	-0.53584100	-0.01544200
C	-0.48251000	0.70054700	-0.04480100
C	-1.96486500	0.93755100	0.09979900
C	1.57431900	-0.51904800	-0.29005000
C	1.79914900	0.95854200	-0.73506900
C	0.54698500	1.79120800	-0.31910800
O	-0.19093900	-2.85600500	0.42688000
C	1.98537100	-1.50356400	-1.40302000
C	2.33937200	-0.85360600	1.00637300
O	3.74340500	-0.75411300	0.75839800
C	0.83038300	2.62002200	0.95849600
C	0.09701400	2.74091300	-1.44756000
O	-2.19858100	2.18240100	0.76797100
C	-2.81955300	-1.91402300	-1.07771000
H	-2.59380100	-2.37274200	1.00869300
H	-2.37729000	1.00332000	-0.92124600
H	2.72237400	1.36791000	-0.31381100
H	1.90641000	0.98962200	-1.82558200
H	1.81328800	-2.53876200	-1.09609800
H	3.04727100	-1.38168000	-1.63767300
H	1.40898000	-1.31426400	-2.31598800
H	2.07011300	-1.86877800	1.32702900
H	2.03879900	-0.15746600	1.80362700
H	4.19871100	-0.98590700	1.58256700
H	1.16569400	1.98208700	1.78364700
H	-0.06154900	3.15909800	1.28465700
H	1.62530700	3.34701900	0.75150400
H	0.89610100	3.45738900	-1.67309900
H	-0.79507000	3.30356800	-1.15614600
H	-0.12554000	2.18734200	-2.36751800
H	-3.14802800	2.37053700	0.69165400
H	-3.91291300	-1.84978200	-1.05209100
H	-2.54256400	-2.92672400	-1.38824600
H	-2.45615100	-1.21770200	-1.84169000
C	-2.63695700	-0.21826000	0.85185000
H	-3.72758700	-0.10223300	0.80928900
H	-2.34266500	-0.13187600	1.90523700

Table S13 - Cartesian coordinates of lowest-energy conformers

B3LYP/PCM(MeOH)/6-31G(d) opt

Total energy = -484503.2026 kcal mol^{-1}

Comp. 5 conformer 1

C	-2.56860000	-0.81730000	0.30350000
C	-1.16770000	-1.41670000	0.14890000
C	-0.03950000	-0.46750000	0.20170000
C	-0.20990000	0.87430000	0.14960000
C	-1.57250000	1.52030000	0.13640000
C	1.43050000	-0.87460000	0.29900000
C	2.12280000	0.49990000	0.55210000
C	1.11270000	1.63190000	0.18520000
O	-1.00100000	-2.62840000	0.01090000
C	1.69870000	-1.85660000	1.45670000
C	1.88300000	-1.50690000	-1.03290000
O	0.79320000	-2.24400000	-1.59210000
C	1.44180000	2.24460000	-1.19850000
C	1.10820000	2.75190000	1.24510000
O	-1.53510000	2.72390000	-0.63620000
C	-3.66450000	-1.77670000	-0.16470000
H	-2.69420000	-0.64350000	1.38600000
H	-1.81530000	1.77360000	1.18410000
H	3.05780000	0.58480000	-0.00960000
H	2.38170000	0.57760000	1.61460000
H	1.19240000	-2.81110000	1.28830000
H	2.77290000	-2.04450000	1.54860000
H	1.34140000	-1.43960000	2.40540000
H	2.20730000	-0.70990000	-1.71510000
H	2.74370000	-2.16890000	-0.85490000
H	1.09910000	-2.61610000	-2.43380000
H	1.46480000	1.47830000	-1.98110000
H	0.70340000	2.99810000	-1.48090000
H	2.43100000	2.71730000	-1.16220000
H	2.10140000	3.21310000	1.30500000
H	0.38340000	3.53150000	0.99140000
H	0.86160000	2.35800000	2.23820000
H	-2.37800000	3.18210000	-0.48820000
H	-3.58830000	-2.73820000	0.34980000
H	-4.65370000	-1.35170000	0.03550000
H	-3.58570000	-1.96580000	-1.24160000
C	-2.63470000	0.55260000	-0.39480000
H	-3.63190000	0.99090000	-0.26190000
H	-2.47360000	0.42940000	-1.47390000

Table S14 - Cartesian coordinates of lowest-energy conformers

B3LYP/PCM(MeOH)/6-31G(d) opt

Total energy = -484501.1839 kcal mol^{-1}

Comp. 5 conformer 2

C	2.38767300	-1.29634600	-0.35627300
C	2.68896200	0.11814800	-0.87972400
C	0.88775200	-1.60246500	-0.35109500
C	-0.05015800	-0.46365900	-0.31987100
C	0.36729400	0.81917800	-0.23828100
C	1.82283600	1.21959600	-0.25311600
C	-1.57263300	-0.59237000	-0.30684200
C	-2.02095500	0.89873500	-0.41538200
C	-0.78753500	1.80442400	-0.11016200
O	0.50078000	-2.77138600	-0.32394500
C	-2.10865200	-1.42502600	-1.48832000
C	-2.02516500	-1.22552000	1.02502800
O	-3.45355600	-1.24613200	1.06733300
C	-0.84979500	2.39353400	1.31975900
C	-0.68189700	2.96436700	-1.12124800
O	2.19170600	1.52347000	1.10578300
C	2.96586500	-1.57752100	1.04835500
H	2.83311100	-2.02632200	-1.04385300
H	2.51579600	0.14286500	-1.96355500
H	3.74915600	0.36037800	-0.72925300
H	1.93235100	2.13004700	-0.85977800
H	-2.86220300	1.11137500	0.25104300
H	-2.36789500	1.09457300	-1.43658800
H	-1.75436800	-1.01358600	-2.44047500
H	-1.77693100	-2.46446000	-1.41828700
H	-3.20292000	-1.40717900	-1.49401300
H	-1.61767000	-2.24244100	1.09601200
H	-1.62396800	-0.64161700	1.86725400
H	-3.71099300	-1.66683600	1.90220100
H	-0.95469900	1.60593400	2.07344700
H	0.05598300	2.96039300	1.55269900
H	-1.71408600	3.06328600	1.40503200
H	-1.59260600	3.57379800	-1.08426700
H	0.16525400	3.62294400	-0.89824300
H	-0.56777300	2.59219100	-2.14611400
H	3.09069700	1.89149500	1.07952300
H	2.55753200	-0.88535700	1.78890000
H	2.73835200	-2.60378400	1.35387500
H	4.05489600	-1.45577200	1.03411000
